# Polydactyly-derived allogeneic chondrocyte cell-sheet transplantation with high tibial osteotomy as regenerative therapy for knee osteoarthritis

**DOI:** 10.1038/s41536-022-00272-1

**Published:** 2022-12-16

**Authors:** Kosuke Hamahashi, Eriko Toyoda, Miya Ishihara, Genya Mitani, Tomonori Takagaki, Nagatoshi Kaneshiro, Miki Maehara, Takumi Takahashi, Eri Okada, Ayako Watanabe, Yoshihiko Nakamura, Reiko Kato, Ryo Matoba, Takehiko Takagi, Hidenori Akutsu, Akihiro Umezawa, Hiroyuki Kobayashi, Tadashi Akamatsu, Masayuki Yamato, Teruo Okano, Masahiko Watanabe, Masato Sato

**Affiliations:** 1 Department of Orthopaedic Surgery, Surgical Science, Tokai University School of Medicine, 143 Shimokasuya, Isehara, Kanagawa 259-1193 Japan; 2 Center for Musculoskeletal innovative Research and Advancement (C-MiRA), Tokai University Graduate School, Isehara, Kanagawa 259-1193 Japan; 3 Department of Medical Engineering, National Defense Medical College, 3-2 Namiki, Tokorozawa, Saitama 359-8513 Japan; 4 Cell Processing Center, Tokai University Hospital, 143 Shimokasuya, Isehara, Kanagawa 259-1193 Japan; 5 National Institute of Health Sciences, 3-25-26 Tonomachi, Kawasaki-ku, Kawasaki, Kanagawa 210-9501 Japan; 6 DNA Chip Research Inc., 1-15-1 Kaigan, Suzue Baydium 5F Minato-ku, Tokyo, 105-0022 Japan; 7 National Center for Child Health and Development, 2-10-1 Okura,Setagaya-ku, Tokyo, 157-8535 Japan; 8 Department of Clinical Pharmacology, Tokai University School of Medicine, 143 Shimokasuya, Isehara, Kanagawa 259-1193 Japan; 9 Department of Plastic Surgery, Surgical Science, Tokai University School of Medicine, 143 Shimokasuya, Isehara, Kanagawa 259-1193 Japan; 10 Institute of Advanced Biomedical Engineering and Science, Tokyo Women’s Medical University, 8-1 Kawada-cho, Shinjuku, Tokyo, 162-8666 Japan; 11 Cell Sheet Tissue Engineering Center, Department of Pharmaceutics and Pharmaceutical Chemistry, University of Utah, 30 South 2000, East Salt Lake, UT 84112 USA

**Keywords:** Translational research, Cell transplantation

## Abstract

Allogeneic cell therapies are not fully effective in treating osteoarthritis of the knee (OAK). We recently reported that transplantation of autologous chondrocyte cell-sheets along with open-wedge high tibial osteotomy promoted hyaline cartilage repair in humans. Here we describe our regenerative therapy for OAK using polydactyly-derived allogeneic chondrocyte cell-sheets (PD sheets) and temperature-responsive culture inserts. Ten patients with OAK and cartilage defects categorized arthroscopically as Outerbridge grade III or IV received the therapy. Cartilage viscoelasticity and thickness were assessed before and after transplantation. Arthroscopic biopsies obtained 12 months after transplantation were analyzed histologically. Gene expression was analyzed to evaluate the PD sheets. In this small initial longitudinal series, PD sheet transplantation was effective in treating OAK, as indicated by changes in cartilage properties. Gene marker sets in PD sheets may predict outcomes after therapy and provide markers for the selection of donor cells. This combined surgery may be an ideal regenerative therapy with disease-modifying effects in OAK patients.

## Introduction

Osteoarthritis (OA) is the most common cause of loss of mobility and is the most prevalent form of musculoskeletal disease worldwide^1,2^. OA adversely affects the quality of life, work productivity, and cost of health care. We recently reported on our clinical research on the effects of combined surgery and autologous chondrocyte cell-sheet transplantation in OA^3^. No serious adverse events related to the combined surgery were observed during the treatment and follow-up period, and excellent regeneration of the articular surface with hyaline cartilage was confirmed in second-look arthroscopic surgery. All biopsy samples of regenerated cartilage revealed strong staining for Safranin O and expression of type II collagen (COL2). The Knee Injury and Osteoarthritis Outcome Score (KOOS) and Lysholm Knee Score (LKS) improved significantly after surgery, and these improvements were maintained for more than 3 years. However, autologous chondrocyte cell-sheet transplantation has several limitations. For example, arthroscopic surgery, which requires the harvesting of cartilage and synovial tissues, is needed before transplantation; the harvested cells and their properties differ between individuals and are occasionally harvested in insufficient numbers to fabricate cell sheets, and chromosomal abnormalities are often found in cells obtained from elderly patients with osteoarthritis of the knee (OAK). Given these limitations and the fact that articular cartilage is immune tolerant, we consider the use of allogeneic cell sheets as a viable option for avoiding the need to harvest tissue.

Allogeneic mesenchymal stem cells (MSCs), adipose-derived stem cells, and umbilical cord stem cells are often used in clinical trials of treatments for OAK. However, most of these methods involve cell therapies using intra-articular injection. These therapies tend to achieve temporary improvement of symptoms not but structural reconstruction or the production of native hyaline cartilage. The recent increase in the use of cell-based strategies for cartilage repair has generated an urgent need for potent and safe off-the-shelf cell sources. We have focused on polydactyly surgery as a way of obtaining tissue samples as allogeneic cell sources and have succeeded in fabricating polydactyly-derived allogeneic chondrocyte cell-sheets (PD sheets) using temperature-responsive culture inserts^4,5^. Recently, Kondo et al. reported the preclinical safety and efficacy of polydactyly-sourced juvenile cartilage derived-chondrocyte sheets in vitro and in vivo using a rat focal osteochondral defect model^6^. PD chondrocytes proliferate rapidly, establish a layered structure with a sufficient extracellular matrix, and form sheets that can be easily manipulated. Although the number of autologous chondrocyte sheets that can be fabricated is limited^4^, theoretically, more than 600 PD sheets can be fabricated from passage 2 (P2) cells and more than 3000 PD sheets can be fabricated from P3 cells. Here, we describe our use of PD sheet transplantation combined with a high tibial osteotomy (HTO) and suggest that it is an ideal form of regenerative therapy for patients with OAK.

**Fig. 1:**
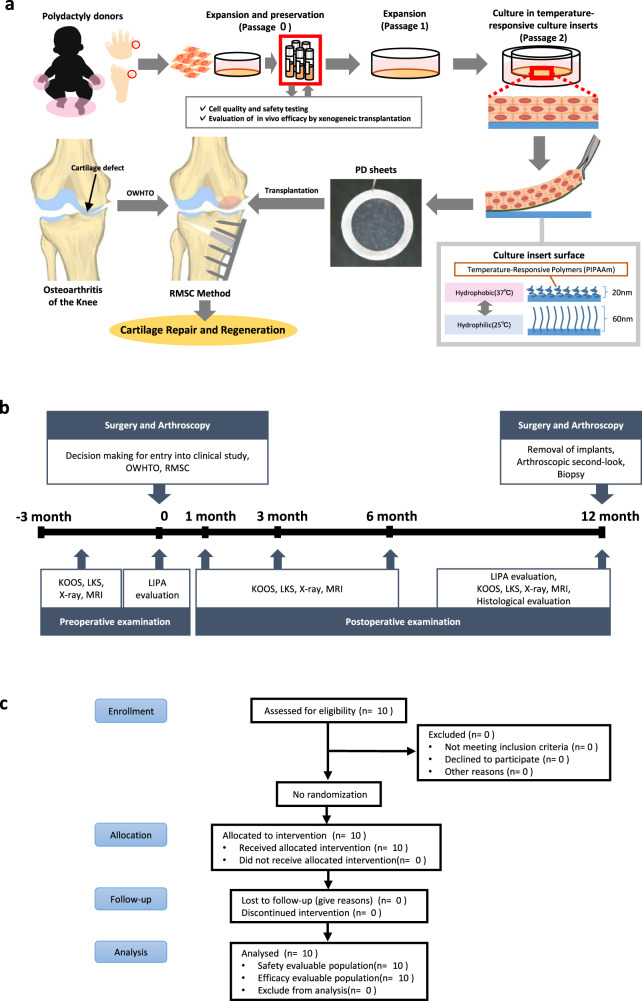
Surgical interventions and transplantation of chondrocyte sheets. **a** Combination of conventional surgical intervention and implantation of chondrocyte sheets. Polydactyly-derived allogenic chondrocyte sheets (PD sheets) were fabricated from cartilage tissue obtained from polydactyly surgery. In the cell-processing center, isolated chondrocytes were passaged once and stocked at −180 °C. To fabricate PD sheets, cells were thawed and passaged once and then seeded on temperature-responsive culture inserts. The PD sheets were further cultured for 14–16 days and transplanted. OAK patients were first treated with conventional surgical treatments OWHTO, followed by the removal of unhealthy tissue, marrow stimulation, and chondrocyte sheet transplantation (RMSC method). **b** The overall protocol for the clinical study. The final decision about entry into the clinical study was made during the arthroscopic evaluation. For patients meeting the inclusion criteria, LIPA was used to assess their cartilage defects. RMSC removal of unhealthy tissue, marrow stimulation, and chondrocyte sheet transplantation, OWHTO open-wedge high tibial osteotomy, LIPA laser-induced photoacoustic, KOOS Knee Injury and Osteoarthritis Outcome Score, LKS Lysholm Knee Score, MRI magnetic resonance imaging. **c** The CONSORT flow diagram. CONSORT diagram for a single-arm, open-label, uncontrolled, comparative study (that is, enrolment, intervention allocation, follow-up, and data analysis).

We have designed a combination therapy (Fig. 1a) in which conventional surgical treatment for OAK, which is covered by National Health Insurance in our country, was followed by the removal of unhealthy tissue, bone marrow stimulation, and chondrocyte sheet transplantation (the “RMSC method”) to treat cartilage defects^3^. This study was designed to evaluate the effectiveness of the total treatment but not the add-on effects of each treatment. HTO for patients with OAK is a joint-preserving procedure that has garnered attention throughout the world. Articular cartilage is sometimes regenerated after the improvement of whole leg alignment by HTO, but this is mainly fibrous cartilage, which has different properties from hyaline cartilage^7,8^. We hypothesized that PD sheet transplantation in conjunction with HTO may accelerate the regeneration of native cartilage, such as hyaline cartilage, reduce the symptoms of OAK, and help to maintain the biological qualities of the knee joint.

Temperature-responsive culture dishes or inserts (CellSeed Inc., Tokyo, Japan), whose use was first reported by Okano et al.^9,10^, have a special intelligent surface. Using these inserts, chondrocyte sheets can be produced by maintaining the extracellular matrix and then reducing the temperature to release the sheets, with no need for enzymatic digestion.

We were the first to report the application of layered chondrocyte sheets for articular cartilage repair^11,12^. We have reported evidence from animal studies indicating the potential of these layered chondrocyte sheets in the treatment of nontraumatic arthritis in rat^13^, a partial defect in rabbit articular cartilage^14^, and osteochondral defects in rat^15,16^, rabbit^17–19^, and minipig^20^ cartilage. These types of defects are usually present in OAK. We have also investigated the mode of action of the layered chondrocyte sheets, and we have suggested that chondrocyte sheets exert their ongoing regeneration effects in articular cartilage through the continuous secretion of humoral factors such as melanoma inhibitory activity (MIA), transforming growth factor β (TGF-β), and prostaglandin E_2_, which are important in cartilage regeneration^21,22^.

Ten patients with OAK and cartilage defects were enrolled in this study and received therapy with PD sheets. A rigorous evaluation protocol (Fig. 1b) was designed to assess the endpoints of safety and efficacy of the therapy and to evaluate the clinical and structural outcomes. The properties of the transplanted allogeneic PD sheets were also evaluated thoroughly using gene expression analysis to investigate their potential use for predicting the clinical and structural outcomes of the therapy. In this small initial longitudinal case series, we wanted to determine whether the expression of specific marker gene sets in PD sheets could provide potential markers for predicting the outcomes of this new regenerative therapy for OAK.

## Results

### Predicting clinical and structural outcomes by analyzing PD cell sheets

**Fig. 2:**
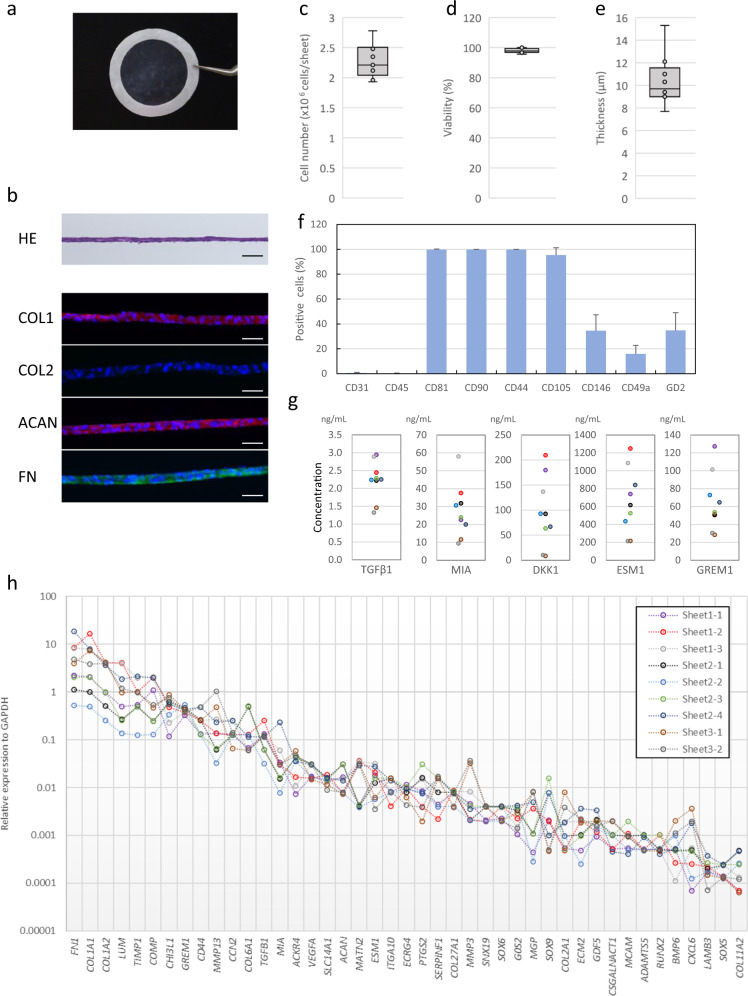
Properties of PD sheets. **a** PD sheets were handled using a circular PVDF support membrane. **b** Representative images of cross-section of PD sheet. Histological staining with hematoxylin and eosin (HE), immunohistochemical staining for type I collagen (COL1), type II collagen (COL2), aggrecan (ACAN), and fibronectin (FN). scale bar; 50 μm. **c**–**e** Distribution of transplanted PD sheet characteristics of nine lots: The box represents the interquartile range of the values. The top and bottom of the whiskers represent the maximum and minimum values. The line inside the box represents the median values. Cell number (c) and cell viability (d) were determined after enzymatic digestion of a cell sheet. **e** Thickness of PD sheet was measured using microscopic images of cross sections. **f** Flow cytometric analysis of surface markers for the cells in the PD sheets. The PD sheets were digested enzymatically and analyzed by single-color staining. Data are expressed as the mean ± SD of the percentage of surface marker-expressing cells from nine lot of sheets (Supplementary Fig. 2). Cells were positive for CD81, CD90, CD44, and CD105 and negative for CD31 and CD45. Staining for CD146, CD49a, and GD2 differed between the lots. **g** Concentrations of humoral factors secreted by PD sheets. Each mark indicates a lot of sheets. Some marks overlap. **h** The gene expression profile of transplanted sheets was analyzed using qPCR for cartilage-related genes, and the results are reported relative to the expression of *GAPDH*. A total of nine lots of PD sheets were fabricated using cryopreserved cells from three polydactyly patient donors. Three lots were from donor 1 and were used to create sheets 1-1, 1-2, and 1–3 in **h**. Four lots were from donor 2 and were used to create sheets 2-1, 2-2, 2-3, and 2–4. Two lots were from donor 3 and were used to create sheets 3-1 and 3-2. Each lot was used for one patient, except for sheet 3-2, which was transplanted into two patients (patients 9 and 10). Data with the same color were from the same lot of PD sheets in **g**, **h**.

Figure 2 shows the properties of PD sheets transplanted in this study. The PD sheets can be handled using a circular white support membrane of polyvinylidene difluoride (Fig. 2a). The multilayered structures were confirmed by histological staining (Fig. 2b). Immunostaining showed that the sheets strongly expressed fibronectin (FN), COL1, and aggrecan (ACAN). PD sheets exhibited almost no COL2 expression (Fig. 2b). The cells in the PD sheets were dedifferentiated, and the properties of chondrocyte sheets were different from those of hyaline cartilage. Figure 2c–e shows the distribution of properties of the transplanted PD sheets with respect to cell number, viability, and thickness, respectively. Figure 2f shows the results of the flow cytometric analysis of the dispersed PD sheets. Almost all cells from PD sheets were positive for cluster of differentiation 81 (CD81) and mesenchymal stem cell markers, CD90 (Thy-1), CD44 (receptor for hyaluronic acid), and CD105 (endoglin). CD146 (melanoma cell adhesion molecule)-positive and GD2 (disialoganglioside GD2)-positive cells were present at a frequency of 20–70% and CD49a (integrin α1)-positive cells at a frequency of 5–25%. Almost all cells were negative for the blood lineage markers CD31 (platelet endothelial cell adhesion molecule-1) and CD45 (leukocyte common antigen). The marker expression was similar to that of adult knee chondrocyte sheets that were transplanted in a previous clinical study that used autologous cells. The production of transforming growth factor β1 (TGF-β1) and previously identified efficacy-associated factors^5^, melanoma inhibitory activity (MIA), Dickkopf WNT signaling pathway inhibitor 1 (DKK1), endothelial cell-specific molecule 1 (ESM1), and gremlin 1 (GREM1) confirmed by enzyme-linked immunosorbent assay (ELISA) (Fig. 2g). Quantitative polymerase chain reaction (qPCR) analysis of the genes expressed by the PD sheets revealed widespread expression of cartilage-related genes of interest (Fig. 2h).

### Clinical outcomes

No serious adverse events related to the combined surgeries, such as deep infection or pseudoarthrosis, were observed during the follow-up period. Other adverse events were reported and included deep vein thrombosis in one patient, pain in 10 patients, leukocytosis in 10 patients, and increased C-reactive protein levels in 10 patients. Deep vein thrombosis was healed by conservative therapy. These adverse events were considered to have been related to the open-wedge high tibial osteotomy (OWHTO). We believe that these were unlikely to have been related to the transplantation of PD sheets.

**Fig. 3:**
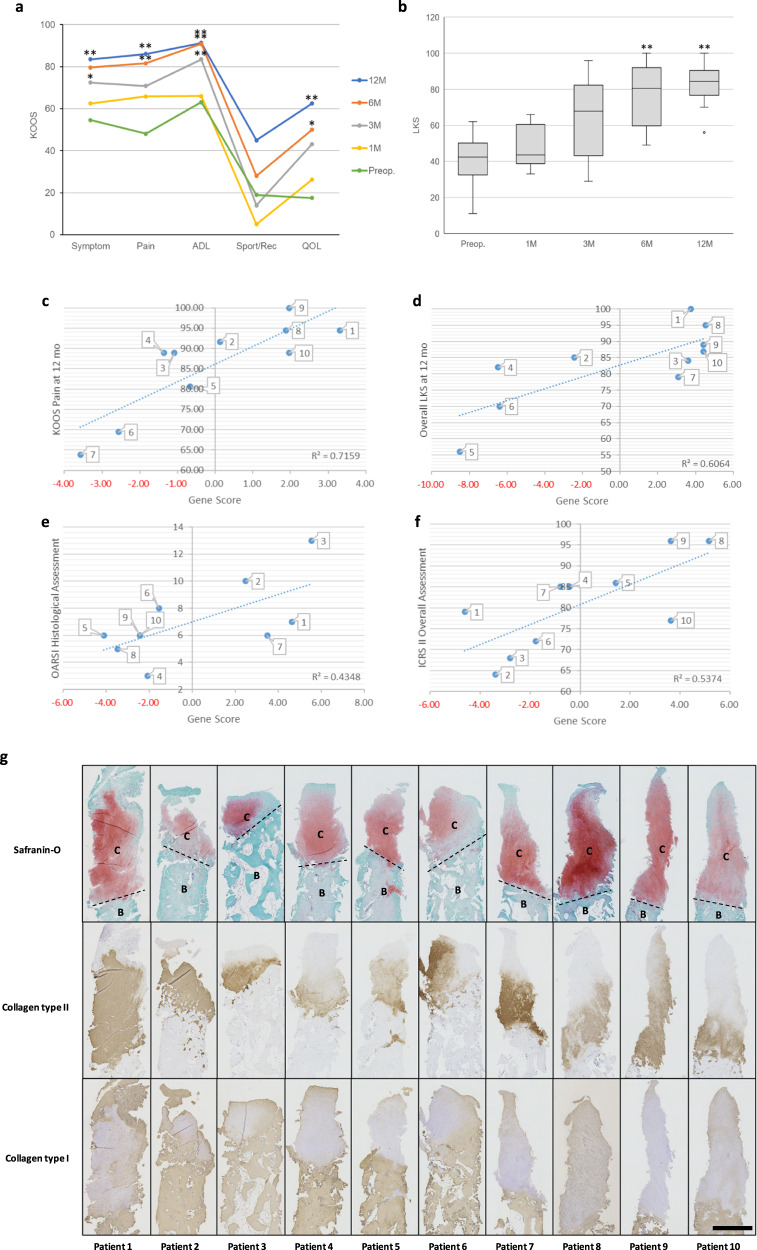
Clinical and histological outcomes. **a** KOOS was used to assess the effects on knee-related parameters. The patient averages for the KOOS subscales, symptom, pain, ADL, and QOL improved significantly from the baseline. *p < 0.05, **p < 0.01. ADL activities of daily living, QOL quality of life. **b** Patient average for LKS improved significantly from the baseline. The box represents the interquartile range of the scores. The top and bottom of the whiskers represent the maximum and minimum scores, except for the data at 12 months that contains the outlier. The line inside the box represents the median score. *p < 0.05, **p < 0.01. **c**–**f** Correlational analysis was used to select a gene marker set that was predictive of each of the outcome measures. The scatter plot shows the correlations between the gene scores and KOOS pain at 12 months (**c**), LKS at 12 months (**d**), and OARSI (**e**) and ICRS II (**f**). The gene score was calculated from the gene expression of the predictive gene marker set for KOOS pain (*PTGS2*, *TGFB1*, *MIA*, and *G0S2*), the LKS (*COL1A2*, *MATN2*, *SOX9*, and *TGFB1*), and the OARSI histological score (*CCN2*, *COMP*, *ACKR4*, *ESM1*, and *GREM1*), and ICRS II overall assessment (*COL2A1*, *COL27A1*, *ACKR4*, and *ESM1*), as described in the Methods section (Supplementary Tables 4–7). The number assigned to each symbol indicates the corresponding patient. **g** Histological results of patient biopsies and results from clinical evaluations. C = articular cartilage, B = subchondral bone, Dashed line = boundary between articular cartilage region and subchondral bone region. Biopsies of femoral medial condyle were taken from areas of regenerated cartilage 12 months postoperatively. scale bar = 1 mm. Histological sections from all patients stained for Safranin O. Immunostaining showed the expression of type II collagen (COL2) in all patients.

The LKS improved significantly from 40.1 ± 13.9 points preoperatively to 80.5 ± 15.7 points at the final follow-up. All of the KOOS subscales (symptom, pain, function in daily living, function in sport and recreation, and quality of life) also improved significantly (Fig. 3a, b and Supplementary Table 1, 2).

### Structural outcomes

The cartilage defects and locations are described in Table 1. Briefly, the mean total defect area was 15.9 cm^2^ for one patient and >20 cm^2^ in four patients. Ten patients had medial femoral condyle (MFC) defects (mean area 10.7 cm^2^), six patients had trochlear (Tr) defects (mean area 4.6 cm^2^), and five patients had kissing defects of the tibia (mean area 4.0 cm^2^).

**Table 1:** Clinical examinations and outcomes.

Patient number	1	2	3	4	5	6	7	8	9	10
Age (years)	56	45	57	52	51	52	53	58	58	57
BMI (kg/m^2^)	27.3	34.6	31.2	29.0	25.8	35.5	25.9	23.5	23.5	27.3
Duration of symptoms (years)	3	8	1	2.5	2	1	1	3	1	6
KL grade	2	4	3	3	3	2	3	4	3	3
Outerbridge classification	MFC 3 Tr 3	MFC 4 Kiss 4 Tr 3	MFC 3-4	MFC 4	MFC 4 Kiss 4	MFC 3-4 Tr 3-4	MFC 3-4	MFC 4 Kiss 4 Tr 3	MFC 3-4 Kiss 4 Tr 3-4	MFC 3-4 Kiss 4 Tr 3-4
Location and Size of the defect (cm^2^)	MFC5.1 Tr 4.5	MFC 8.8 Kiss 4.2 Tr 4.6	MFC 9.0	MFC 15.0	MFC 15.0 Kiss 5.0	MFC 9.0 Tr 4.0	MFC 12.0	MFC 10.5 Kiss 4.5 Tr 5.3	MFC 11.0 Kiss 6.0 Tr 5.0	MFC 12.0 Kiss 4.5 Tr 4.0
Number of transplanted cell sheet (total)	MFC 6 Tr 4 (10)	MFC 4 Kiss 4 Tr 4 (12)	MFC 9 (9)	MFC 13 (13)	MFC 12 Kiss 3 (15)	MFC 9 Tr 6 (15)	MFC 14 (14)	MFC 6 Kiss 3 Tr 3 (12)	MFC 6 Kiss 5 Tr 4 (15)	MFC 6 Kiss 5 Tr 4 (15)
Thickness of RC (preop. value) (mm)	MFC 2.87 (0.90)	MFC 3.30 (NA) Tr 3.25 (2.45)	MFC 2.90 (1.28)	MFC 3.64 (1.51)	MFC 3.94 (1.86)	MFC 2.64 (1.75) Tr 2.54 (2.07)	MFC 3.47 (1.66)	MFC 4.44 (0.77) Tr 3.89 (1.56)	MFC 4.10 (1.03)	MFC 4.37 (1.17) Tr 4.02 (1.17)
Viscoelasticity ratio by LIPA (preop. value)	MFC 0.86 (0.53)	MFC 0.83 (NA) Tr 0.87 (0.66)	MFC 0.91 (0.53)	MFC 0.90 (NA)	MFC 1.00 (NA)	MFC 0.94 (0.84) Tr 0.89 (0.86)	MFC 0.96 (0.71)	MFC 0.93 (NA) Tr 0.90 (0.71)	MFC 0.96 (0.82)	MFC 0.98 (NA) Tr 0.86 (0.76)
ICRS II (Overall Assessment)	79	64	68	85	86	72	85	96	96	77
OARSI histological score	7	10	13	3	6	8	6	5	6	6
Mankin score	4	8	5	2	5	5	3	5	4	4
MOCART 2.0 Knee Score (preop. value)	50 (10)	60 (20)	50 (25)	75 (25)	80 (30)	75 (55)	35 (20)	50 (20)	60 (40)	55 (30)

*BMI* body mass index, *KL grade* Kellgren–Lawrence grade, *MFC* medial femoral condyle, *Kiss* Kissing lesion of tibia of tibial plateau, *Tr* trochlea, *RC* regenerated cartilage, *LIPA* laser-induced photoacoustic measurement, *NA* not applicable, *ICRS* International Cartilage Repair Society.

The femorotibial angle and percentage of mechanical axis (%MA) changed significantly from 179.4° ± 2.8° to 168.9° ± 2.8° and from 24.1 ± 10.8 to 67.5 ± 9.2 from before to after transplantation, respectively.

Magnetic resonance imaging (MRI) confirmed the regeneration of cartilage in areas that lacked cartilage preoperatively (Supplementary Figure 1). The results of the Magnetic Resonance Observation of Cartilage Repair Tissue 2.0 (MOCART 2.0) score improved significantly (Table 1).

The results of the laser-induced photoacoustic (LIPA) evaluation are shown in Table 1. The viscoelastic characteristics of the regenerated cartilage are expressed as the ratio relative to that of normal cartilage in the same joint. The characteristics improved after transplantation, and the mean thickness of the regenerated cartilage was 3.54 mm.

### Histological outcomes

All biopsies revealed strong staining for Safranin O and expression of COL2 (Fig. 3g). Although the degree to which regeneration occurred varied between patients and some cases represent a sign of degeneration, these results nevertheless suggest that regeneration of hyaline cartilage had occurred. The results of the Osteoarthritis Research Society International (OARSI) histological score, the Mankin score, and the International Cartilage Repair Society (ICRS II) overall assessment are shown in Table 1. The quality of regenerated cartilage was very high, with an ICRS II average score of 80.8 (64–96, 0: fibrous tissue, 100: hyaline cartilage), as shown in Table 1 although fibrocartilage was also present.

### Potential markers for predicting the outcomes

To identify PD sheets that promoted superior outcomes, gene marker sets predictive of the postoperative clinical and structural outcomes were identified based on the correlational analysis between the gene expression profile and outcomes. The analyses were described previously in the study on autologous cell sheets^3^. Potential marker sets were selected from cartilage-related genes (list in Supplementary Table 3) with the highest probability of appearance in a matrix of 100 sets of Pearson correlation coefficients using the leave-five-out process.

The gene score was calculated using the gene expression data of the marker set as described in Methods (Supplementary Tables 4–7). Figure 3c–f shows the scatter plots indicating the gene scores of the transplanted PD sheets and clinical outcomes for each individual case. The marker sets for each outcome score were as follows: for KOOS (Fig. 3c) *PTGS2*, *TGFB1*, *MIA*, and *G0S2*; for LKS (Fig. 3d) *COL1A2*, *MATN2*, *SOX9*, and *TGFB1*; and for OARSI histological score (Fig. 3e) *CCN2*, *COMP*, *ACKR4*, *ESM1*, and *GREM1*; for ICRS II overall assessment (Fig. 3f) *COL2A1*, *COL27A1*, *ACKR4*, and *ESM1*.

### Case report (Patient 5)

**Fig. 4:**
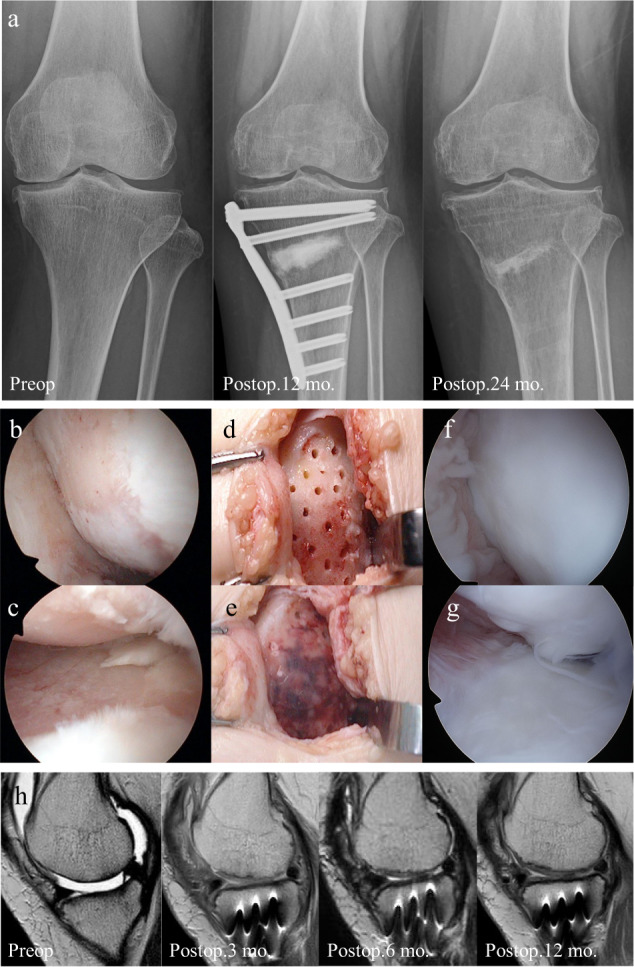
Case presentation of regenerative therapy (patient 5). X-ray photographs (**a**), arthroscopic images (**b**–**g**), and magnetic resonance imaging (**h**). **a** X-ray preoperative photograph of the left knee confirmed a varus deformity and narrowing of the medial joint space. X-ray photograph 12 months postoperatively showed that the alignment was maintained after OWHTO. At 24 months postoperatively (12 months after implants were removed), the alignment was maintained and beta-tricarcium phosphate graft has been absorbed. **b**, **c** Preoperative cartilage defect of the medial femoral condyle and kissing lesion of tibial plateau caused exposure of the subchondral bone. **d** Intraoperative pictures of medial femoral condyle after microfracture. **e** PD sheets were transplanted just as putting on it without suturing. **f**, **g** The presence of regenerated cartilage was confirmed at the second look arthroscopic view (12 months postoperatively). **h** Sagittal view of the medial femorotibial joint. T2-weighted images were obtained using a 3.0-Tesla MRI. Time course from the preoperative cartilage defect to regenerated cartilage after surgery. The preoperative MR image shows a broad area of the cartilage defect in the medial condyle of the femur, a kissing lesion in the tibia, and the collection of synovial fluid. The regenerated cartilage was detected 3 months after surgery and was maintained with time. The irregular subchondral bone caused by marrow stimulation was initially detected and became smooth by 12 months.

A 51-year-old woman underwent PD-sheet transplantation combined with OWHTO for OAK of the left knee. The femorotibial angle and %MA changed from 183° to 167° and 11.4 to 68.9, respectively (Fig. 4a). Subchondral bone was exposed across a wide area in the MFC (Fig. 4b) and medial tibial plateau (Fig. 4c). After using LIPA to evaluate the viscoelastic properties in the residual cartilage and subchondral bone, we performed microfracture (Fig. 4d) and transplantation of PD sheets (Fig. 4e). One year after transplantation, regenerated cartilage that fully covered the defect area was observed in the MFC (Fig. 4f) and medial tibial plateau (Fig. 4g). Preoperative MRI showed a full-thickness cartilage defect at the MFC. This defect area appeared to be coated with PD sheets and was filled with regenerative tissue after 3 months, and the regenerative tissue was clearly visible and maintained at 12 months after transplantation (Fig. 4h).

## Discussion

We have confirmed the mode of action, safety, and efficacy of PD sheets in experiments using the xenogeneic model^4^ and in other studies^19,21,23^ before undertaking the clinical study.

In the current study, we speculate that the mode of action of humoral factors of PD sheets was the most likely mechanism because we did not use immunosuppressants. In our earlier research, we found variability in the therapeutic potential of PD sheets after xenogeneic orthotopic transplantation and recognized the importance of the selection of donors for future studies^5^. Cavalli et al.^24^ reported that polydactyly chondrocytes can be expanded in vitro and maintain a fast and steady proliferative rate for up to five passages, which is an important characteristic of an off-the-shelf cell source. The proper passage of PD cells is also critical in clinical trials. To address this issue, we identified potential gene marker sets for predicting the outcome of PD sheet transplantation (Fig. 3c–f). The marker genes for each outcome score are different. *TGFB1* appeared in common in KOOS and LKS, both of which are in the self-report symptom questionnaire. *ESM1* and *ACKR4* appeared in the OARSI histological score and ICRS II overall assessment, both scores being related to biopsy tissue evaluation. The marker set of genes can be useful for selecting the allogeneic cell source for cell sheet fabrication prior to clinical use.

Because we analyzed the expression of cartilage-related genes in only a small initial longitudinal series, other genes may have been extracted if a different gene set had been used for analysis. The current marker set of genes may be improved by replacing the genes with newly identified, more predictive ones.

Articular cartilage sometimes regenerates after bone marrow stimulation or abrasion arthroplasty combined with HTO. However, these regenerated tissues are mainly fibrous cartilage, which differs from native hyaline cartilage^7,8,25–27^ and does not provide the same long-term load-bearing effects as hyaline cartilage. Previously, we compared clinical outcomes between patients undergoing OWHTO and total knee arthroplasty (TKA). In these patients, the KOOS was lower in patients who underwent OWHTO than in those who received TKA, and residual symptoms such as knee grinding, clicking, and stiffness were worse after OWHTO than after TKA^28^. On this point, in the current study, we confirmed that regeneration of hyaline cartilage had occurred after PD sheet transplantation combined with OWHTO. Our findings suggest that long-term therapeutic effects along with improvement of lower limb alignment can be expected after the combination treatment.

We have previously reported on our use of autologous chondrocyte sheet transplantation and have continued to observe the long-term clinical outcomes^3^. For the previous study, we had been directed by the Japan Ministry of Health, Labour and Welfare (MHLW) to transplant cell sheets only into knees with defects measuring <4.2 cm^2^, which is the size of one cell sheet. However, for the current study, we were able to prepare enough PD sheets to cover larger defects and we did not limit patient enrollment because of defect size. In this clinical study, some patients had defects measuring >20 cm^2^. Generally, the common exclusion criteria that define the “red knee” are lesions measuring >8 cm^2^, malalignment, age >55 years, kissing lesions, and diffuse cartilage thinning^29^, and some patients with red knee were enrolled in this current study. Although there were no serious adverse events after transplantation of the PD sheets, we must still complete phase III clinical trials with a well-controlled comparative study by restricting enrollment to patients without red knee.

The number of transplanted PD sheets, locations of defects, and total size of the defects were not related to the clinical outcomes and histological scores. However, these results may have been influenced by the fact that all patients received a sufficient number of PD sheets to cover the defects completely.

Our study has three limitations. First, the study included only 10 patients with general OA who underwent OWHTO. This associated surgery may have affected the outcomes of the therapy. Second, we observed promising outcomes for the use of PD sheets for at least 1.5 years, but longer-term observations are needed to evaluate this new combination therapy fully. Finally, our clinical study was designed as a single-arm, nonrandomized, and uncontrolled study, and further studies with more patients and conditions are needed.

We have been continuing to develop the techniques for preserving and transporting cell sheets in preparation for future commercialization. We continue to research the vitrification method to facilitate the cryopreservation of cell sheets while maintaining their macro- and microstructures to ensure a high rate of the viability of the constituent cells^30^. The circulating vitrification bag and vitrification storage box are useful for the long-term preservation of vitrified cell sheets, which will make the clinical application of cryopreserved cell sheets even more feasible^31^.

We confirmed the safety and efficacy of the transplantation of PD sheets. Ten patients underwent PD sheet transplantation combined with OWHTO. Second-look arthroscopy 1 year after transplantation showed regenerated cartilage that covered the defect areas fully. LIPA evaluation suggested that the regenerated cartilage had normal mechanical properties, and histological analysis of biopsy samples indicated hyaline-like cartilage. This combination surgery may provide an ideal regenerative therapy with disease-modifying effects in patients with OAK.

## Methods

The experiments were performed under the approval and guidance of the Clinical Research Review Committee of Tokai University School of Medicine. We presented these preclinical research data to the MHLW in Japan and applied for a Class I regenerative medicine provision plan under the law for the safety of regenerative medicine. The permit was issued on April 27, 2016, and clinical research using PD sheets was allowed to begin. Informed consent was obtained from the parents or guardians of the donors in all cases. Some surgical specimens were irreversibly de-identified. All experiments handling human cells and tissues were performed in line with the tenets of the Declaration of Helsinki. The animal experiments were approved by the Institutional Animal Experiment Committee at Tokai University and were performed in accordance with the guidelines of the Institutional Regulation for Animal Experiments and the Fundamental Guideline for Proper Conduct of Animal Experiment and Related Activities in Academic Research Institutions under the jurisdiction of the Ministry of Education, Culture, Sports, Science, and Technology for animal handling and care.

### Study design and setting

Our clinical study was designed as a single-arm, nonrandomized, uncontrolled comparative study. This study (UMIN Clinical Trials Registry, UMIN000015205, and Japan Registry of Clinical Trials, jRCTa030190242) was approved by the Institutional Review Board of the Tokai University School of Medicine and by the MHLW of Japan. Written informed consent was obtained from all participants. The CONSORT flow diagram is shown in Fig. 1c.

### Donor selection

The cell sheets for transplantation were fabricated from donor cells that exhibited an ability to initiate hyaline cartilage regeneration after xenogeneic orthotopic transplantation into a Japanese white rabbit model of cartilage full-thickness defect^5,19^. We also performed array comparative genomic hybridization (array CGH) and karyotype analysis of the P2 PD cells used to fabricate the cell sheets and as long-term cultured cells (P12 cells) to assess the genetic stability of the PD cells during in vitro fabrication. No copy number alterations were detected by array CGH, and no karyotype abnormalities, as judged by the International System for Human Cytogenetic Nomenclature guidelines, were found in the cells used as the cell source.

### Fabrication of PD sheets

Cartilage tissue was obtained from three patients (average age: 12.3 months, range: 10–15 months, one boy and two girls) who underwent polydactyly surgery at Tokai University Hospital. A summary of the PD sheet fabrication process is shown in Fig. 1a. Cartilage tissue was minced with scissors and then incubated in Dulbecco’s modified Eagle’s medium/F12 (DMEM/F12; Gibco, Grand Island, NY, USA) supplemented with 20% fetal bovine serum (FBS; AusGeneX, Molendinar, Australia, or SAFC Biosciences, Lenexa, KS, USA), 1% antibiotic–antimycotic solution (AB; Gibco), and 5 mg/mL collagenase type 1 (Worthington, Mannheim, Germany) for 1.5 h at 37 °C in a humidified atmosphere of 5% CO_2_ and 95% air. The cell suspension was washed and passed through a 100 μm strainer (BD Falcon, Franklin Lakes, NJ, USA).

The collected cells were seeded at a density of 0.5 to 1 × 10^4^ cells/cm^2^ into six-well culture plates (Corning Inc., Corning, NY, USA) in DMEM/F12 supplemented with 20% FBS and 1% AB, and incubated at 37 °C. After 4 days, 100 μg/mL ascorbic acid (AA; Nissin Pharmaceutical, Yamagata, Japan) was added to the medium, and the medium was replaced every 3 or 4 days. Cells were cryopreserved using STEM-CELLBANKER™ (ZENOAQ, Fukushima, Japan) when they reached subconfluence. Cells were thawed and seeded once at a density of 0.5 to 1 × 10^4^ cells/cm^2^ in DMEM/F12 supplemented with 20% FBS, 1% AB, and 100 μg/mL AA. When the cells reached subconfluence, they were detached using TrypLE Express (Thermo Fisher Scientific, Tokyo, Japan) and seeded onto temperature-responsive culture inserts (CellSeed Inc., Tokyo, Japan) at 1 × 10^4^ cells/cm^2^. The medium was replaced every 3 or 4 days. After 2 weeks, the culture plates were kept at 25 °C for 30 min to promote detachment of PD sheets from the inserts, and the PD sheets were harvested. To analyze the properties of PD sheets using real-time qPCR, flow cytometric analysis, and histological and immunohistochemical staining, PD sheets were harvested on day 13 of culture.

### Cell count and viability

PD sheets were washed in Dulbecco’s phosphate-buffered saline (DPBS; Gibco). The sheets were then incubated in TripLE Express® (Gibco) at 37 °C for 15 min and centrifuged at 1500 rpm for 5 min. The cell sheets were resuspended in 0.25 mg/mL collagenase P (Roche, Basel, Switzerland) at 37 °C for up to 30 min and then centrifuged at 1500 rpm for 5 min. The isolated cells were finally resuspended in DMEM/F12, and the cell count and viability were determined using the trypan blue exclusion assay.

### Flow cytometric analysis

After the cell count was obtained, isolated cells were washed with DPBS containing 0.2% bovine serum albumin (BSA; Sigma-Aldrich, St. Louis, MO, USA) and 1 mM ethylenediaminetetraacetic acid (EDTA; Gibco). About 1.5 × 10^5^ cells were mixed in each tube with the following antibodies: hCD31–fluorescein isothiocyanate (FITC) (1:10; IM1431U, Beckman & Coulter, Brea, CA, USA), CD44–FITC (1:10; 555478, BD Biosciences, Franklin, NJ, USA), hCD45–FITC (1:10; A07782, Beckman & Coulter), hCD81–allophycocyanin (APC) (1:10; 551112, BD Biosciences), hCD90–APC (1:100; 559869, BD Biosciences), CD105–phycoerythrin (PE) (1:5; A07414, B76299, Beckman & Coulter), CD146–PE (1:5; 5050-PE100T, BioCytex, Marseilles, France), CD49-PE (1:5; 559596, BD Biosciences), and GD2 (1:10; 554272, BD Biosciences). The cells were incubated for 90 min at 4 °C and then washed with DPBS containing 0.2% BSA and 1 mM EDTA. GD2 was recognized by incubating the cells with the secondary antibody FITC-conjugated anti-mouse IgG (1:20; 349031, BD Biosciences) for 10 min at 4 °C. Fluoroprobe-labeled mouse IgG1 antibody (1:10; Beckman & Coulter) was used as a negative control. Stained cells were analyzed using a FACSVerse™ cell sorter (BD Biosciences) (Supplementary Fig. 2).

### Histological and immunohistochemical staining

PD sheets were harvested after culture and then embedded and frozen in optimal cutting temperature compound (Sakura Finetek Japan, Tokyo, Japan). Sections 10 μm thick were stained with hematoxylin and eosin using standard methods (Supplementary Figure 3). Sections 20 μm thick were immunostained with anti-human COL1 (1:200; 1310-01, Southern Biotech, Birmingham, AL, USA), COL2 (1:200; F-57, Kyowa Pharma Chemical, Toyama, Japan), FN (1:1000; MA5-11981, Thermo Fisher Scientific, Waltham, MA, USA), and ACAN (1:20; 967800, R&D Systems, Minneapolis, MN, USA) at 4 °C overnight. The sections were washed and incubated at room temperature for 1 h with the secondary antibody Alexa Fluor 488-conjugated goat anti-mouse Ig (1:200; A-11017, Thermo Fisher Scientific) for COL2 and FN or Alexa Fluor 546-conjugated donkey anti-goat Ig (1:400, A-11056, Thermo Fisher Scientific) for COL1 and ACAN. After immunostaining, the sections were washed and mounted with VECTASHIELD Antifade Mounting Medium with 4′,6-diamidino-2-phenylindole (Vector Laboratories, Burlingame, CA, USA). Microscopic images were captured under a BZ-X810 microscope (Keyence, Osaka, Japan).

### RNA isolation

The PD sheets were disrupted in TRIzol Reagent (Thermo Fisher Scientific) using a SHAKE Master Neo device (Bio Medical Science, Tokyo, Japan), and the total RNA was isolated using an RNeasy Mini Kit (Qiagen, Valencia, CA, USA) according to the manufacturer’s instructions. RNA quantity and quality were determined using a Nanodrop One spectrophotometer (Thermo Fisher Scientific) and an Agilent Bioanalyzer (Agilent Technologies, Santa Clara, CA, USA).

### Real-time qPCR

Total RNA was converted to cDNA using QuantiTect Reverse Transcription Kit (Qiagen). A TaqMan PreAmp Master Mix Kit and TaqMan Gene Expression Assays (both from Thermo Fisher Scientific) (Supplementary Table 3) were used to preamplify the cDNA according to the manufacturer’s instructions. TaqMan qPCR was performed using a 7500 qPCR System or QuantiStudio 3 (both from Thermo Fisher Scientific). The relative expression values for each gene (–ΔCt values) were calculated using *GAPDH* as the internal control.

### Measurement of humoral factors

To collect the supernatants of the fabricated PD sheets, the sheets were transferred to 3 mL of DMEM/F12 supplemented with 1% FBS and 1% AB on day 14 of culture and cultured for another 72 h. Supernatants were collected and centrifuged at 15,000 *g* for 10 min to remove cell debris. The concentrations of TGF-β1 (R&D Systems), MIA (Roche), Dickkopf WNT signaling pathway inhibitor 1 (Thermo Fisher Scientific), endothelial cell-specific molecule 1 (ESM1; Abcam, Cambridge, UK), and gremlin 1 (GREM1; Cloud-Clone Corp., Katy, TX, USA) were measured using ELISA kits. The signal detected for the blank medium containing 1% FBS was subtracted to adjust for the proteins contained in FBS.

### Clinical research study of PD sheet transplantation

Ten patients (four men and six women) who had cartilage defects categorized arthroscopically as Outerbridge grade III or IV were enrolled and received the therapy. Their mean age at the time of the operation was 54 years (range 45–58), and their mean body mass index was 28.3 kg/m^2^ (range 23.5–35.5). The Kellgren and Lawrence grades from knee roentgenograms were grade 2 in two patients, grade 3 in six, and grade 4 in two (Table 1). We performed PD sheet transplantation in the first patient in February 2017 and the last in December 2019.

The inclusion criteria for this clinical research study were having medial compartment OAK, which is usually indicated for OWHTO (Table 2). In other clinical research studies of autologous chondrocyte sheet transplantation, the size of the damaged cartilage lesion was regulated as <4.2 cm^2,3^. However, there was no regulation of size for this clinical research study of PD sheet transplantation because more than ten PD sheets could be prepared in advance. The total number of defects to be transplanted with PD sheets was 21 (10 for MFC, 6 for Tr, 5 for kissing lesions); the average defect sizes were 10.8, 4.6, and 4.0 for MFC, Tr, and kissing, respectively. The maximum total defect size was 22.0 cm^2^ in one patient (Table 1).

**Table 2:** Inclusion and exclusion criteria of the clinical study.

Eligibility inclusion criteria	Eligibility exclusion criteria
Patients who meet all of the following inclusion criteria and have the ability to consent will be included in the study. 1 20–60 years of age and any sex 2 Have a knee cartilage lesions 3 Outerbridge classification Grade III or IV 4 Cartilage defect in the patellofemoral joint or condyle of the femur that can be covered by a fabricated cell sheet and is indicated for conventional marrow stimulation, osteochondral autografts, etc.	1 Patients who have difficulty providing informed consent 2 Complications that interfere with surgery under general anesthesia or that affect knee surgery 3 Patients with infectious diseases (including testing positive for HBV, HCV, HIV, HTLV-1, syphilis) 4 Patients with systemic inflammatory diseases, such as rheumatoid arthritis

*HBV* hepatitis B virus, *HCV* hepatitis C virus, *HIV* human immunodeficiency virus, *HTLV-1* human T cell leukemia virus type 1.

### Surgery and rehabilitation

We designed a combination therapy in which conventional surgical treatment for OAK, which is covered by National Health Insurance in Japan, was followed by the RMSC method (Fig. 1a). Cryopreserved allogeneic chondrocytes were thawed and cultured for 3 weeks to fabricate PD sheets before the transplantation surgery. A total of nine lots were fabricated: three lots from donor 1, four lots from donor 2, and two lots from donor 3. Each lot was used for one patient, except for the ninth lot, which was transplanted into two patients. We performed OWHTO as described by Takeuchi et al.^32^ and aimed to achieve a postoperative %MA of 62.5. After biplane (oblique and proximal tuberosity) osteotomy, artificial bone was transplanted into the open gap and fixed at the osteotomy site using TomoFix® (DePuy Synthes, Bettlach, Switzerland) or TriS® (Olympus Terumo Biomaterials, Tokyo, Japan).

After OWHTO, the knee joint was opened about 5 cm using the medial parapatellar approach. The MFC, medial tibial plateau, and patellofemoral joint were observed, and RMSC was performed (Supplementary Movie). Briefly, after the removal of unhealthy tissue and bone marrow stimulation (microfracture in seven patients and abrasion arthroplasty in three), PD sheets were transplanted to cover all of the cartilage defect surface. PD sheets can be transplanted without any suturing or periosteum patch because of their excellent adhesive properties. This RMSC method^3^ is an efficient way to regenerate hyaline cartilage because it inserts bone marrow-derived MSCs and various humoral factors secreted by PD sheets into the cartilage defect space. PD sheets were transplanted into the MFC in 10 patients, medial tibial plateau in five, and patellofemoral joint in six. The mean number of transplanted sheets was 13 (9–15). The Outerbridge grades and location and size of the cartilage defects are shown in Table 1.

All patients were immobilized immediately after surgery using a plaster splint that was maintained for 2 weeks at 20° of knee flexion to avoid disturbing the transplanted sheets. Patients then started range-of-motion exercise and partial weight bearing at 2 weeks, and full weight bearing at 4 weeks after surgery. In general, low-impact activities were started at 6 months, and high-impact activities were allowed at 8 months.

### Clinical outcomes

We evaluated the clinical outcomes using the patient-oriented KOOS and LKS preoperatively and at 1, 3, 6, and 12 months postoperatively^33,34^.

### Structural outcomes

Imaging assessment was performed using X-ray photographs and MRI. X-ray photographs were examined for knee alignment, condition of the subchondral bone, and progression of OAK. Progress was evaluated using the Kellgren–Lawrence grading scale preoperatively and postoperatively. MRI examinations were performed using an Achieva 3.0-T TX scanner (Philips Healthcare, Best, The Netherlands), within a TX SENSE Knee eight-channel coil (Philips Healthcare). The images were taken with 10° of knee flexion. Sagittal and coronal MR images were used to evaluate the cartilage defect areas preoperatively and postoperatively.

For evaluation of the therapy, we used the MOCART 2.0 method described by Schreiner et al.^35^ (Table 1). Arthroscopic evaluation of the cartilage defects included their condition, size, and Outerbridge grade, and was performed preoperatively and postoperatively. The LIPA method was used to evaluate the viscoelastic properties of cartilage preoperatively and postoperatively (Supplementary Movie). The use of LIPA was approved by the Institutional Review Board for Clinical Research at Tokai University, and the method has been applied clinically to evaluate the mechanical properties of cartilage in patients^36^. The LIPA method was used arthroscopically during the first surgery (PD sheet transplantation and OWHTO) and 12 months postoperatively (second-look and removal of implants).

### Histological outcomes

Histological outcomes were evaluated 12 months postoperatively using an arthroscopically performed biopsy taken from near the center of the regenerated cartilage. Samples were decalcification by 10% EDTA Solution B (Wako Pure Chemical Industries, Osaka, Japan) for 9 days at 4 °C. And then, samples were embedded in paraffin wax, and 3 μm sections were cut, deparaffinized, stained with Safranin O, and immunostained for COL1 and COL2 using previously reported methods^15^. The ICRS II overall assessment^37^ and the OARSI histological score were assessed independently by three trained orthopedic surgeons. Microscopic scoring of the cartilage was evaluated using the method described by Little et al.^38^, who provided representative images for each score. There were minimal variations in scoring between the three scorers. The Mankin score was obtained similarly^39,40^ Structural compromise (0–6 points), loss of matrix staining (0–4 points), cellularity anomalies (0–3 points), and violation of tidemark integrity (0 or 1 point) were graded using this scale, with normal cartilage given a score of 0 out of 14 points.

### Predictive gene marker sets

Predictive gene marker sets were selected from the 43 genes and control genes examined (*GAPDH* and *ACTB*) (Supplementary Table 3) as those whose expression correlated with the KOOS pain score and LKS, OARSI histological score and ICRS overall assessment score at 12 months postoperatively. First, 36 genes (Supplementary Tables 4–7) with normalized ΔCt values greater than –10 cycles were identified as having detectable gene expression. For each outcome assessment, Pearson correlation coefficients were calculated between the expression level of each of the genes and the individual scores (KOOS, LKS, OARSI histological score, and ICRS overall assessment score) using the leave-five-out process, and the genes were ranked from highest to lowest for the absolute values of the coefficients. From this, a matrix of 100 sets of Pearson correlation coefficients was calculated. The representative genes were selected independently as predictive markers for each of the outcome measures by selecting >0.5 of absolute value for the Pearson correlation coefficients for the genes listed according to their probability of appearance. Finally, four or five gene sets with the highest probability of appearance were selected as the predictive markers. The gene score was calculated as the normalized value of the expression of each gene based on linear functions: Gene score = Σ(–ΔCt value) (Supplementary Tables 4–7).

### Statistical analysis

Data were analyzed using repeated-measures analysis of variance and the post hoc Bonferroni test to identify differences between the pre-operative and postoperative scores for clinical outcomes (*n* = 10) for KOOS scores and overall LKS. *p* values < 0.05 were significant.

## Supplementary information

The clinical trial protocol

Supplementary Materials

Supplemental Movie

Supplemental Information Files

## Data Availability

The data sets generated and/or analyzed during the current study are available from the corresponding author on reasonable request.
