# Supplementary material for: Polydactyly-derived allogeneic chondrocyte cell-sheet transplantation with high tibial osteotomy as regenerative therapy for knee osteoarthritis
Source: NPJ Regen Med. 2022 Dec 16;7:71. doi: 10.1038/s41536-022-00272-1 (PMC9755241; doi:10.1038/s41536-022-00272-1)
Supplement: Supplementary file 1 — The clinical trial protocol [file 41536_2022_272_MOESM1_ESM.pdf]

The Clinical study for the joint treatment by allogeneic cell sheet

Study protocol

Institutions
Department of Orthopedic Surgery, Surgical Science,
Tokai University School of Medicine

Department of Plastic Surgery, Surgical Science,
Tokai University School of Medicine

Department of Orthopedic Surgery,
Tokai University Hospital

Department of Plastic Surgery, Surgical Science,
Tokai University Hospital

Cell Processing Center,
Tokai University Hospital

Research Representative
Masato Sato

Department of Orthopedic Surgery, Surgical Science,
Tokai University School of Medicine
143 Shimokasuya, Isehara-shi, Kanagawa-ken 259-1193
0463-93-1121
0463-96-4404
sato-m@is.icc.u-tokai.ac.jp

April 17, 2020
6th edition

Confidentiality

This clinical study protocol is to be considered confidential information only to be shared with the review board; clinical/principal investigators, co-investigators, and research collaborators of the clinical study; the Dean of Tokai University School of Medicine; the Chairman of the Tokai University Hospital; and various departments of the Tokai University School of Medicine and Tokai University Hospital involved in this clinical study.

Summary

1. Objectives

This study aims (1) to perform knee joint cartilage treatment using allogeneic chondrocyte sheets, whose therapeutic effect has been confirmed in animal experiments, in patients with knee joint cartilage defects; (2) to confirm knee cartilage restoration after fabricating allogeneic chondrocyte sheets using chondrocytes from surgical remains obtained from patients with polydactyly and transplanting the sheets into cartilage defect regions in patients with knee joint cartilage defects; (3) to objectively assess the safety of this novel treatment as the primary endpoint; and (4) to assess its efficacy as the secondary endpoint.

2. Target disease

Cartilage defects caused by osteoarthritis of the knee

3. Study design

A single-arm, open-label, uncontrolled, comparative study will be carried out to confirm the safety and efficacy of cartilage restoration through allogeneic chondrocyte sheet transplantation as treatment for cartilage defects caused by osteoarthritis of the knee.

4. Endpoints

Primary endpoint: safety (determined by serious adverse event rate)

Secondary endpoint: efficacy (determined by histological evaluation, arthroscopy, MRI, X-ray, laser-induced photoacoustic method, clinical assessment by J-KOOS, and Lysholm Knee Score)

5. Subjects of the study

Inclusion criteria

Patients who meet all of the following inclusion criteria and have the ability to consent will be included in the study.

① Patients of any sex and between 20 and 60 years of age

② Patients with knee cartilage lesions

③ Patients with arthroscopic findings indicative of cartilage lesion grade III or IV in the Outerbridge classification

④ Patients with cartilage defect in the patellofemoral joint or condyle of the femur

that can be covered by a fabricated cell sheet and is indicated for conventional bone marrow stimulation, osteochondral autograft transfer, etc.

Exclusion criteria

Patients who meet at least one of the following exclusion criteria will not be included in the study.

① Patients who have difficulty providing informed consent

② Patients with complications that interfere with surgery under general anesthesia or that affect knee surgery

③ Patients with infectious diseases, such as hepatitis B virus (HBV), hepatitis C virus (HCV), human immunodeficiency virus (HIV), or human T-cell leukemia virus (HTLV), and syphilis.

④ Patients with systemic inflammatory diseases, such as rheumatoid arthritis

6. Study methods

① Tissue collection: Knee cartilage tissue will be collected from surgical remains obtained from polydactyly surgery.

② Fabrication of allogeneic chondrocyte sheets: Polydactyly tissue will be transported to the processing room where cells will be isolated, passaged, and cryopreserved. Samples from preserved polydactyly-derived chondrocyte cells with confirmed safety and properties of cell sheets will be selected as allogeneic chondrocytes for transplantation. Once the target patient has been enrolled, the fabrication of cell sheets will commence 3 weeks before the transplant date. The cells will be seeded in temperature-responsive culture dishes to produce allogeneic chondrocyte sheets.

③ Transplantation of cell sheets: Allogeneic chondrocyte sheets will be transplanted into the site of cartilage defect in combination with high tibial osteotomy (HTO).

④ Postoperative follow-up: Hospital stay will be approximately 1 month (rehabilitation and rest will be similar to those of patients undergoing HTO alone). Follow-ups will be conducted 1, 3, 6, and 12 months after surgery. Thereafter, follow-ups will be conducted every 6 months to 1 year for 5 years.

7. Study period and target sample size

Clinical study period

Five years from March 11, 2016 (this period encompasses 1 to 2 years for collecting, storing, evaluating, and validating cells for transplantation, as well as the period

for clinical study on transplantation and follow-up after 1 year)

Expected enrollees

Twenty cases of polydactyly tissue donation and ten cases of transplantation

8. Participating institutions

Department of Orthopedic Surgery, Surgical Science, Tokai University School of Medicine

Department of Plastic Surgery, Surgical Science, Tokai University School of Medicine

Department of Orthopedic Surgery, Tokai University Hospital

Department of Plastic Surgery, Surgical Science, Tokai University Hospital

Cell Processing Center, Tokai University School of Medicine

Table of Contents

１. Objectives • 7
２. Clinical study implementation system • 7
３. Background • 12
４. Ethical considerations • 17
５. Patient selection (Patients for transplantation) • • • • • • • • • • • • • • • 21
６. Clinical study period and target enrollments • • • • • • • • • • • • • • • • • 23
７. Enrollment • 24
８. Study methods • 25
９. Postoperative tests, endpoints, and schedule • • • • • • • • • • • • • • • • • 31
１０. Expected adverse events • 32
１１. Handling of adverse events • 33
１２. Medical fees and compensation • 36
１３. Statistical considerations • 36
１４. Disclosure of study information and publication of results • • • • • • • • • • 38
１５. Use of samples and information in new research • • • • • • • • • • • • • • • 38
１６. Management of samples and information (storage and disposal) • • • • • • • 38
１７. Study funding and conflicts of interest of investigators, etc. • • • • • • • • • 39
１８. Response to inquiries from subjects and related individuals • • • • • • • • • 39
１９. Response to the provision of medical care to subjects after study • • • • • • • 39
２０. Monitoring and audit • 39
２１. Attached reference materials • 40

1. Objectives

The cell sheets used in this study will be used in the treatment of intractable knee cartilage using Japan's cutting-edge technology. At present, we are conducting clinical research using human stem cells with autologous chondrocyte sheets. Eight patients underwent transplantation without any major adverse events. Issues with treatment using autologous chondrocyte sheets include the sacrifice of cartilage in healthy regions, the limited number of viable donor regions, and the low growth ability of the cartilage among older adults. We expect the development of a novel treatment method that uses autologous chondrocyte sheets. In this study, we will carry out knee cartilage treatment using allogeneic chondrocyte sheets, whose therapeutic effect has been confirmed in animal experiments, in patients with knee cartilage lesions. Chondrocytes isolated from surgical remains obtained from polydactyly patients will be passaged and cryopreserved as allogeneic cells. An allogeneic chondrocyte sheet will be fabricated by culturing cells, whose safety and characteristics in a cell sheet have been confirmed in advance, from the cryopreserved cells. These sheets will be transplanted into the cartilage defect regions in the knee of patients. Thereafter, the restoration of the knee cartilage will be confirmed. The safety of this novel treatment will be objectively assessed as the primary endpoint. Furthermore, its efficacy will be assessed as the secondary endpoint.

2. Clinical study implementation system

1) Clinical research representative

This study will be collectively supervised by:
Masato Sato
Professor, Department of Orthopedic Surgery, Surgical Science, Tokai University School of Medicine
Director, Cell Processing Center, Tokai University Hospital
(Department of Orthopedic Surgery, Tokai University Hospital)

2) Clinical study investigators

Polydactyly surgery and collection of allogeneic tissues will be handled by:
Tadashi Akamatsu
Professor, Department of Plastic Surgery, Surgical Science, Tokai University School of Medicine
(Department of Plastic Surgery, Tokai University Hospital)

Clinical diagnosis, transplantation, and postoperative assessment will be handled

by:
Genya Mitani
Associate Professor, Department of Orthopedic Surgery, Surgical Science, Tokai University School of Medicine
(Department of Orthopedic Surgery, Tokai University Hospital)

Clinical diagnosis, transplantation, and postoperative assessment will be handled by:
Tomonori Takagaki
Lecturer, Department of Orthopedic Surgery, Surgical Science, Tokai University School of Medicine
(Department of Orthopedic Surgery, Tokai University Hospital)

Polydactyly surgery and allogeneic tissue collection will be handled by:
Kotaro Imagawa
Lecturer, Department of Plastic Surgery, Surgical Science, Tokai University School of Medicine
(Department of Plastic Surgery, Tokai University Hospital)

Clinical diagnosis, transplantation, and postoperative assessment will be handled by:
Kosuke Hamahashi
Lecturer, Department of Orthopedic Surgery, Surgical Science, Tokai University School of Medicine
(Department of Orthopedic Surgery, Tokai University Hospital)

Evaluation of the properties of the cell sheets and safety will be handled by:
Taku Ukai
Lecturer, Department of Orthopedic Surgery, Surgical Science, Tokai University School of Medicine
(Department of Orthopedic Surgery, Tokai University Hospital)

Evaluation of the properties of the cell sheets and safety will be handled by:
Munetaka Yokoyama
Assistant Professor, Department of Orthopedic Surgery, Surgical Science, Tokai University School of Medicine

(Department of Orthopedic Surgery, Tokai University Hospital)

Evaluation of the properties of the cell sheets and safety will be handled by:
Yoshiki Tani
Assistant Professor, Department of Orthopedic Surgery, Surgical Science, Tokai University School of Medicine
(Department of Orthopedic Surgery, Tokai University Hospital)

Evaluation of the properties of the cell sheets and safety will be handled by:
Daichi Takizawa
Assistant Professor, Department of Orthopedic Surgery, Surgical Science, Tokai University School of Medicine
(Department of Orthopedic Surgery, Tokai University Hospital)

Evaluation of the properties of the cell sheets and safety will be handled by:
Yasuyuki Sogo
Assistant Professor, Department of Orthopedic Surgery, Surgical Science, Tokai University School of Medicine
(Department of Orthopedic Surgery, Tokai University Hospital)

Evaluation of the properties of the cell sheets and safety will be handled by:
Naoki Takatori
Assistant Professor, Department of Orthopedic surgery, Surgical Science, Tokai University School of Medicine
(Department of Orthopedic Surgery, Tokai University Hospital)

3) Study collaborators

Recording of fabrication and manufacturing process of the cell sheets and the evaluation of the properties of the cell sheets and safety will be handled by:
Yoshihiko Nakamura
Assistant to the Director, Cell Processing Center, Tokai University Hospital

Recording of fabrication and manufacturing process of the cell sheets, the evaluation of the properties of the cell sheets and safety, and the monitoring will be handled by:
Eriko Toyoda

Specific Researcher, Department of Orthopedic Surgery, Surgical Science, Tokai University School of Medicine

Recording of fabrication and manufacturing process of the cell sheets and the evaluation of the properties of the cell sheets and safety will be handled by:
Eri Okada
Researcher, Department of Orthopedic Surgery, Surgical Science, Tokai University School of Medicine

Recording of fabrication and manufacturing process of the cell sheets and the evaluation of the properties of the cell sheets and safety will be handled by:
Maehara Miki
Specific Researcher, Department of Orthopedic Surgery, Surgical Science, Tokai University School of Medicine

Recording of fabrication and manufacturing process of the cell sheets and the evaluation of the properties of the cell sheets and safety will be handled by:
Ayako Watanabe
Specific Researcher, Department of Orthopedic Surgery, Surgical Science, Tokai University School of Medicine

The following person will act as a witness during informed consent briefing and will provide support to patients:
Yuko Chiba
Clinical Study Coordinator, Tokai University Hospital

4) Data management manager/Statistical analysis manager

Hiroyuki Kobayashi
Professor, Department of Clinical Pharmacology, Department of Basic Practice, Tokai University School of Medicine

5) Responsible monitor

Rumiko Shimazawa
Professor, Department of Clinical Pharmacology, Basic Medical Sciences, Tokai University School of Medicine

6) Auditor

Hiroko Shimizu

Professor, Department of Clinical Pharmacology, Basic Medical Sciences, Tokai University School of Medicine

7) Personal information manager

Yoshiaki Ogawa

Director, Information System Department, Tokai University School of Medicine - Isehara Campus

8) Providers and administrators

Masahiko Watanabe

Director, Tokai University School of Medicine

9) Cell processing facilitator/administrator

Yoshiaki Matsumae

Director, Tokai University

10) Principal investigator

Same as research representative

11) Research institutions

Department of Orthopedic Surgery, Surgical Science, Tokai University School of Medicine

Department of Plastic Surgery, Surgical Science, Tokai University School of Medicine

Department of Orthopedic Surgery, Tokai University Hospital

Department of Plastic Surgery, Tokai University Hospital

Cell Processing Center, Tokai University Hospital

143 Shimokasuya, Isehara-shi, Kanagawa-ken 259-1193 TEL: 0463-93-1121

3. Background

1) Current treatment methods and issues concerning the target disease

Since osteoarthritis and other diseases of motor organs are not life threatening, they have been slightly neglected in comparison to life-threatening diseases, such as cancer and heart disease. However, these diseases decrease activities of daily living, which lowers the quality of life, leading to immeasurable human and social loss. According to the 2013 White Paper on Aging Society, individuals aged 65 years and above in Japan reached a record high of 30.79 million, accounting for 24.1% of the total population (aging rate) and creating an unprecedented super-aging society. Furthermore, joint disease is the leading cause of the decrease in healthy life expectancy (cause of requiring support) (2010 Comprehensive Survey of Living Conditions).

It is generally believed that joint cartilage defect, when left untreated, will progress to osteoarthritis in 10 to 20 years. Although mild cartilage damage often has no subjective symptoms, and despite fibrillation on the surface of joint cartilage, early treatment is necessary for mild cartilage damage upon confirmation of the deterioration of the extracellular matrix of chondrocytes, which plays an important role in viscoelasticity and lubrication. Once cartilage damage spreads and becomes severe, it requires major invasive surgeries, such as total knee arthroplasty, which places a huge burden on the patient. At present, autologous chondrocyte transplantation combined with periosteal transplantation is performed for moderate cartilage defects. However, this treatment poses the following problems: (1) it requires the sacrifice of tissue from two healthy regions; (2) the surgery cannot be performed multiple times due to the limited number of viable regions for tissue collection; and (3) older adults have low cartilage growth ability. Therefore, there is a demand for the development of a new treatment method.

Of the main repair methods in Japan, marrow stimulation, involves shaving the defective cartilage as thinly as possible until bleeding from the bone marrow occurs. Thereafter, the defect is repaired using fibroblastic cartilage derived from undifferentiated mesenchymal stem cells in the bone marrow by creating multiple small holes in the bone at 3-mm to 4-mm intervals using a pointed instrument. Although the surgery could be easily performed with arthroscopy, it is best suited for individuals who are young (35 to 40 years old), have been injured within 1 year, have a lesion smaller than 2 cm^2, and have no surgical history. Osteochondral autograft, which has become the standard method for treatment of localized osteochondral defects, involves multiple cylindrical osteochondral pillars taken from non-weight-bearing regions in the knee joint, resulting in cartilage defects at relatively large weight-bearing regions. The site of the cartilage defect is repaired by a complex transplanted autologous hyaline cartilage

and the fibroblastic cartilage regenerated from the gap. Therefore, whether or not the transplanted hyaline cartilage and the regenerated fibroblastic cartilage can be bonded to each other affects the surgical results. Hence, the method is suitable for osteochondritis dissecans, including osteochondritis dissecans of the elbow and ankle joints, patella cartilage defects, cartilage defects associated with ligament and meniscus injuries, osteonecrosis, and cartilage damage secondary to trauma. Furthermore, cartilage defects should ideally be 1 cm^2 to 4 cm^2 and the patient must be 40 years of age or younger. JACC is an insurance-covered processed cellular tissue product that uses healthy periosteum and cartilage. In this method, isolated chondrocytes are embedded and cultured in atelocollagen, the defect is covered with the periosteum, and cultured cells are transplanted into the gap. However, this method is indicated for traumatic cartilage defects measuring 4 cm^2 or larger, and not for osteoarthritis (Table 1).

Table 1. Major repair methods in Japan versus allogeneic chondrocyte sheet transplantation

Methods	Age of indication	Target injury	Process	Repair
Bone marrow stimulation	*ideal for people aged 35 to 40 years	Within 1 year after injury<br>Size: 2 cm^2 or smaller	The defective cartilage is thinned until the bone marrow bleeds and multiple small holes are created in the bone at 3-mm to 4-mm intervals using a pointed instrument	Fibroblastic repair using undifferentiated mesenchymal stem cells of the bone marrow
Osteochondral autograft transfer	*ideal for people aged below 40 years	1 cm^2 to 4 cm^2 cartilage defect.<br>osteochondritis dissecans, including osteochondritis dissecans of the elbow and ankle joints, patella cartilage defects, cartilage defects associated with ligament and meniscus injuries, osteonecrosis, and cartilage damage secondary to trauma	Multiple cylindrical osteochondral pillars collected from non-weight-bearing regions of the knee are implanted into the cartilage defects at weight-bearing regions	Repair by a complex transplanted autologous hyaline cartilage and fibroblastic cartilage regenerated from the gap

Autologous cultured cartilage graft (Jacc ®)	*carried out on people aged 14 to 45 years in clinical trials	Traumatic cartilage deficiency or osteochondritis dissecans in osteoarthritis of the knee (excluding osteoarthritis of the knee). No other treatment and cartilage defect area is at least 4 cm^2	Using healthy periosteum and cartilage, isolated chondrocytes are embedded and cultured in atelocollagen, the defect is covered with the periosteum, and cultured cells are transplanted into the gap.	Repaired by periosteum and autologous cultured chondrocytes
Cell sheet transplantation (autologous)	20- to 60-year-olds with cartilage lesions associated with trauma and degeneration	4.2 cm^2 or smaller knee cartilage lesion caused by trauma or degeneration	Cell sheets are fabricated by co-culturing cartilage tissue and periosteum tissue collected from the patient and are transplanted through marrow injection	Repair of hyaline cartilage by humoral factors from cell sheets and undifferentiated mesenchymal stem cells from bone marrow
Cell sheet transplantation (allogeneic)	20- to 60-year-olds with cartilage lesions associated with trauma and degeneration	Knee cartilage lesion caused by trauma or degeneration	Cell sheets are fabricated from cartilage tissue collected from surgical remains of a tissue donor and are transplanted through marrow injection	Expected repair of hyaline cartilage by humoral factors from cell sheets and chondrocytes derived from undifferentiated mesenchymal stem cells of bone marrows

*: Report of the Working Group for the Development of Standards for Use of Autologous Cultured Cartilage, March 2013, Japanese Orthopedic Association

2) Results of non-clinical studies relevant to this study.

We have conducted basic research on the restoration of joint cartilage mainly using rabbits and miniature pigs:

・Preparation of carrier for cartilage restoration through tissue engineering
Rabbits (Sato M. et al. J Biomed Mate Res A (2003) Nationwide Joint Researc h)

・Building the optimal extracellular environment
Mice (Ishihara M. et al. Biomaterials (2002) Nationwide Joint Research)

Human cells (Ishihara M. et al. J Biomed Mater Res (2001) Nationwide Joint Research)
Rabbits (Ono K. et al. Surgery (2001) Nationwide Joint Research)
Mice (Ishihara M. et al. Wound Repair Regen (2001) Nationwide Joint Research)

・Restoration by allogeneic transplantation of tissue-engineered cartilage
Rabbits (Masuoka K. et al. J Biomed Mater Res B (2005) Nationwide Joint Research)
Rabbits (Sato M. et al. J Biomed Mater Res B (2007) on-campus implementation)
Rabbits (Sato M. et al. Spine (2003) on-campus implementation)
Rabbits (Sato M. et al. Med Biol Eng Comput (2003) on-campus implementation)

・Cartilage of restoration by cartilage sheet transplantation
Rabbits (Masuoka K. et al. J Biomed Mater Res B (2005) Nationwide Joint Research)
Rabbits (Sato M. et al. J Biomed Mater Res B (2007) Nationwide Joint Research)
Rabbits (Sato M. et al. Spine (2003) Nationwide Joint Research)
Rabbits (Sato M. et al. Med Biol Eng Comput (2003) Nationwide Joint Research)
Rabbits/human cells (Sato M. et al. Med Biol Eng Comput (2008) on-campus implementation)

・We confirmed the importance of the interaction between host (patient) cells and donor cells in restoration and found that host (patient) cells will promote repair if minimum cartilage induction initiator (tissue engineering cartilage) is present.
Rabbits (Masuoka K. et al. J Biomed Mater Res B (2005) National Cooperative Study)
Rabbits (Nagai T. et al. Tissue Engineering-Part A (2008) on-campus implementation)

・We first reported restoration of joint cartilage using layered chondrocyte sheets fabricated in temperature-responsive culture dishes for partial injury of joint cart

ilage, which had been thought to be difficult to repair, and clarified the properti es of layered chondrocyte sheets, which have abundant repair abilities.
Rabbits and human cells (Kaneshiro K. et al. Biochem Biophys Res Commun (20 06) on-campus implementation)
Human cells (Kaneshiro N. et. al. Eur Cell Mater (2007) on-campus implementat ion)
Human cells (Mitani G. et al. BMC Biotechnology (2009) on-campus implementat ion)
Patent (International Application Number: PCT/JP2006/303759, International App lication Date: February 28, 2006, International Publication Number: WO2006/093 151, International Publication Date: September 8, 2006, Applicant: Cell Seed, In c., Inventor: Masato Sato et al.)

・We confirmed cartilage restoration in joint cartilage osteochondral defect (osteoc hondral injury).
Pigs (Ebihara G. et al. Biomaterials (2012) on-campus implementation)
Rabbits (Ito S. et al. Biomaterials (2012) on-campus implementation)

・We reported the development of the co-culture method using a temperature-res ponsive insert and the feasibility of fabrication of stable cell sheets within a sho rt period of time despite difficulty in fabrication of osteochondral sheets using h uman cells.
Human cells (Kokubo M. et al. J Tissue Eng Regen Med (2013) on-campus imple mentation)

・We also confirmed that the layered chondrocyte sheet secretes many cytokines, including TGFß, at high concentrations. The cell sheet stays in the injured site f or a long period of time, prevents the outflow of matrix, such as proteoglycan, fr om the injured area, and protects the injured area from catabolic factors in the joint fluid.
Human cells (Hamahashi K. et al. J Tissue Eng Regen Med (2012) on-campus i mplementation)

・We confirmed that the transplanted cell sheet stays in the joint for a long peri od of time and does not migrate to other organs by fabricating a cell sheet from luciferase-expressing transgenic rats and conducting cell tracing using IVIS 21

months after transplantation.
Rats (Takaku Y. et al. Biomaterials (2013) National Joint Research)

In osteoarthritis of the knee, the above series of studies demonstrate the thera peutic effect of cell sheets for partial (damage limited to inside the cartilage) an d osteochondral defects (damage reaching the subchondral bone; also referred to as osteochondral injury), which are often interchanged. Cell sheet engineering, w hich is an original Japanese technology, is a novel cartilage restoration treatmen t approved for clinical research using human stem cells, and targeting osteoarthr itis of the knee. Upon the approval of clinical research using human stem cells by a notification from the Minister of Health, Labour, and Welfare (Medical Affai rs 1003-3) issued on October 3, 2011, 11 patients were enrolled into a clinical st udy for knee joint treatment using cell sheets, which used autologous cells. Eigh t patients underwent the procedure and the clinical study ended 1 year after tra nsplantation. During the clinical study, there were no serious adverse events and a safe and effective joint therapeutic effect was achieved using chondrocyte shee t transplantation. At present, we are investigating the joint therapeutic effect of cell sheet transplantation.

We determined that allogeneic cell sheet transplantation is sufficiently safe bas ed on the results of a clinical study on autologous cell sheet transplantation, as well as the results of safety assessments of allogeneic cells, and those of allogenei c cell transplantation in animal experiments. Furthermore, we also determined th e feasibility of a clinical study that uses cell sheets manufactured from isolated c ells obtained from surgical remains of patients with polydactyly (allogeneic cell so urces).

4. Ethical considerations

1) Compliance with ethical guidelines for clinical research

This clinical study will be conducted in compliance with the ethical principles of the “Ethical Guidelines for Medical and Health Research Involving Human Subjects,” “Rules and Bylaws on Clinical Studies at Hospitals affiliated with the Tokai University School of Medicine,” “Guidance for quality and safety assurance of human cell and tissue derived medical devices and drugs,” "Act on Securing Safety of Regenerative Medicine," “Guidelines on clinical research using human stem cells, Appendix 1,” “Evaluation index on articular cartilage regeneration,” “Publication of next-generation medical device evaluation indices,” "Ensuring the quality and safety of pharmaceutical

and medical devices processed from human-derived (allogeneic) cells and tissues," "Guidelines on securing the quality and safety of human (allogeneic) somatic stem cell processed pharmaceuticals," and this Clinical Study Protocol.

2) Ethics Review Board

For this clinical study, upon deliberation by the Medical Ethics Review Board (certified committee for regenerative medicine under the Act on Securing Safety of Regenerative Medicine; hereinafter, the Committee), the approval of the dean of the School of Medicine (or in some cases, the hospital director) is necessary.

The research representative shall report the progress of the study and the occurrence of diseases and adverse events associated with the implementation of the study to the hospital director, who acts as the supervisor of the institution providing regenerative medicine. The hospital director shall create a disease report and an annual report to be submitted to the certified committee for regenerative medicine. After the comments of the committee have been received, a report shall be submitted to the Ministry of Health, Labour and Welfare. If the research representative and investigator learn of non-compliances with this protocol, this shall be immediately reported to the hospital director. The hospital director shall promptly request the comments of the certified committee for regenerative medicine and report any non-compliance, especially major events, to the Minister of Health, Labour and Welfare.

<Procedures for regular reporting, and changes and termination>

・Regular report

The following matters concerning the status of provision of regenerative medicine, etc., shall be reported to the Minister of Health, Labour and Welfare through the certified committee for regenerative medicine and the director of the Regional Bureau of Health and Welfare.

Items to be reported:

1 Number of persons who received such regenerative medicine, etc.

2 Occurrence status of diseases related to the regenerative medicine, etc., and the subsequent progress

3 Evaluation of the safety and scientific validity of the regenerative medicine, etc.

4 Date of termination if the provision of said regenerative medicine, etc., has been completed

Report deadline:

A report will be submitted every year, 90 days after a given period, starting from the date when the regenerative medicine provision plan is submitted to the director of the Regional Welfare Bureau.

・Notification of changes or termination of the regenerative medicine provision plan

In the event that a plan for provision of regenerative medicine, etc., is to be changed (excluding minor changes), the Minister of Health, Labour and Welfare shall be notified through the director of the Regional Bureau of Health and Welfare after requesting the comments of the certified committee for regenerative medicine on the regenerative medicine provision plan, etc., after the change. The specially certified committee for regenerative medicine will be notified of minor changes within 10 days of the change.

When terminating the provision of regenerative medicine, etc., the specially certified committee for regenerative medicine shall be notified, and the notification of terminating the provision of regenerative medicine, etc., shall be submitted to the Minister of Health, Labour and Welfare through the director of the Regional Bureau of Health and Welfare within 10 days from the date of termination.

3) Method of obtaining informed consent from subjects

Prior to the start of this clinical study, the principal investigator or clinical study investigator will explain the following items to subjects based on explanatory documents and obtain written informed consent. The investigator will explain that consent is at the discretion of the subject and that they will not suffer any unfavorable treatment should they refuse consent. The name of the explaining physician, the signature of the subject, date of consent, etc., will be present in the consent form. The explanatory document and consent form will be given to the subject, and a copy will be kept in an electronic medical record.

The following items shall be described in the explanatory document.

① Matters concerning the study
② Clinical study objectives
③ Clinical study methods (including experimental aspects and inclusion criteria)
④ Expected duration of participation of subjects in clinical study
⑤ Number of subjects expected to participate in clinical study
⑥ Expected clinical benefits and risks or inconveniences (subjects will be informed if there is no anticipated clinical benefit to the subject)
⑦ Presence of alternatives when using patients as subjects, and the expected

significant benefits and risks regarding the treatment methods

⑧ Treatment for subjects in the event of clinical research-related health hazards

⑨ Participation in the clinical study is at the discretion of the subject, and the subject or their legal representative may refuse consent or withdraw the subject's participation in the clinical study at any time. Furthermore, refusal or withdrawal will not result in any disadvantage to the subject or loss of benefits

⑩ Prompt communication to the subjects or their representative if information becomes available that may affect the will of the subject or their representative to continue participation in the clinical study

⑪ Conditions or reasons for discontinuing participation in clinical research

⑫ Preservation of the confidentiality (privacy) of subjects, even when clinical research results are published

⑬ Disclosure of information related to research

⑭ Information that the subject will not incur any cost

⑮ The information desk of the medical institution to be contacted for the names, occupation, and contact details of the principal investigator or investigators, as well as additional information regarding the clinical study or the rights of subjects

⑯ Matters to be observed by the subject

⑰ Explanation of the conflicts of interest

⑱ Attribution of intellectual property

4) Provision of information to subjects

The principal investigator shall promptly inform the subject should there be information that may influence the will of subjects regarding the continuation of their participation in the clinical study. Thereafter, the principal investigator shall confirm whether the subject still wishes to continue participating in the clinical study. Furthermore, this process will be described in the medical record.

5) Handling of personal information and protection of privacy

Since cell sheets are fabricated for each case, all physicians in the department of orthopedic surgery, including the research representative and investigators, know the subjects. Furthermore, since all clinical data are stored in electronic medical records, patient information can be accessed by physicians working at Tokai University Hospital. Therefore, anonymization specific to this clinical study will not be performed. However, anonymity at the same level as regular inpatients will be maintained, so personal

information will be managed using patient IDs.

All clinical study-related records, such as source materials, may be directly viewed during monitoring and audits of the clinical study, and investigations by the certified committee for regenerative medicine and regulatory authorities.

Furthermore, when analyzing clinical data or presenting at academic conferences, the principal investigator will assign and use patient IDs and non-regular subject identification numbers to protect personal information and ensure subject privacy.

Anonymized information, such as the correspondence table, shall be managed by a personal information manager at the Tokai University School of Medicine (head of the Isehara Information System Department).

5. Patient selection (patients for transplantation)

1) Tissue donors

(1) Eligible patients

Patients with polydactyly

(2) Inclusion and exclusion of subjects, and discontinuation criteria

Inclusion criteria

Patients who meet all of the following inclusion criteria and obtain consent from a family member, who is a legally acceptable representative, will be included in the study.

① Patients of any sex from 0 to 5 years of age

② Patients with polydactyly

Exclusion criteria

Patients who meet at least one of the following exclusion criteria will be excluded from this study.

① Informed consent of a family member, who is a legal representative, cannot be obtained

② Patients with significant complications

③ Patients with an infectious disease that may pose a problem (including HBV, HCV, HIV, HTLV, and syphilis).

[Rationale]

[Items set in consideration of the safety of subjects].

Considering the safe and ethical performance of this clinical study and taking into account the reliable data, the items ① to ③ were set.

Discontinuation criteria

The clinical study shall be discontinued immediately after informed consent is obtained if any of the following is applicable.

① The family seeks to withdraw participation from the clinical research

② The general condition of the patient deteriorates during surgery resulting in discontinuation of the surgery

③ The planned surgical procedure is changed due to intraoperative findings

④ This diagnosis is deemed inappropriate due to intraoperative findings

⑤ Any problem leads to a major complication during surgery

⑥ A serious adverse event occurs

2) Subjects (patients for transplantation)

(1) Target disease

Cartilage defects caused by osteoarthritis of the knee

(2) Inclusion and exclusion of subjects, and discontinuation criteria

Inclusion criteria

Patients who meet all of the following inclusion criteria and have the ability to consent will be included in the study.

① Patients of any sex between 20 and 60 years of age

② Patients with knee cartilage injury

③ Patients with arthroscopic findings showing cartilaginous injury above grade III in the Outerbridge classification

④ Patients with a cartilage defect in the patellofemoral joint or condyle of the femur, which can be covered with a fabricated cell sheet and is indicated for conventional marrow stimulation and osteochondral autografts

Exclusion criteria

Patients who meet at least one of the following exclusion criteria will be excluded from this study.

① Obtaining patient informed consent is difficult

② Patients with complications that interfere with general anesthetic surgery or that affect knee surgery

③ Patients with an infectious disease that may pose problems (including HBV, HCV, HIV, HTLV, and syphilis)

④Patients with a systemic inflammatory disease such as rheumatoid arthritis

[Rationale]
[Items set in consideration of the safety of subjects].
Considering the safe and ethical performance of this clinical study and taking into account the reliable data, the items ① to ④ were set.

Discontinuation criteria

The clinical study (cell sheet transplantation) should be discontinued immediately after informed consent is obtained, if any of the following is applicable. However, follow-up and collection of efficacy and safety data should be continued as much as possible.

[Discontinuation criteria for each clinical study subject]

① The patient requests to discontinue participation in the clinical study
②The patient's general condition deteriorates during surgery, resulting in discontinuation of surgery
③ The planned surgical procedure is changed due to intraoperative findings
④ Diagnosis is deemed inappropriate due to intraoperative findings
⑤ Any problem leads to a major complication during surgery
⑥ A serious adverse event occurs
⑦ The production of cell sheets could not be made on schedule

[Criteria for suspension and discontinuation of the entire clinical study]

The entire clinical study will be discontinued or suspended in the event of any of the following.

① The certified committee for regenerative medicine receives an opinion that the clinical research should not be continued
② A serious situation, such as a serious adverse event, occurs
③ A major breach/non-compliance of the study protocol is identified
④ The research representative or investigators determine that the clinical study should be discontinued or cannot be continued

6. Clinical study period and target enrollments

Duration of clinical study: Five years from March 11, 2016 (this period encompasses 1 to 2 years for collecting, storing, evaluating, and validating cells for transplantation, as well as the period for clinical study on transplantation and follow-up after 1 year)

A summary report will be submitted when the analysis of a 1-year observation period is completed.

Expected number of cases is 20 cases of polydactyly tissue donation and 10 cases of transplantation

[Rationale]

Therefore, if there are 20 donors, we estimate that there will be six to seven compatible samples, which is considered sufficient for evaluation and transplantation. Furthermore, as for transplantation cases, 30 surgeries for knee cartilage lesions are conducted at the Tokai University Hospital every year. Considering the selection criteria and the results of clinical research using autologous chondrocytes, the number of expected enrollees was set as the number of cases considered appropriate during the implementation period.

To prevent cross-contamination, separate clean benches and incubators will be used for each sample, and only one sample will be handled at a time. Therefore, the number of cases was determined in consideration of certain restrictions on the use of the cell processing center. It took 1 to 2 years to collect, store, evaluate and validate cells for transplantation, followed by the clinical study (transplant to patients) with a 1-year follow-up period.

7. Enrollment

Candidacy to selection method (enrollment procedure)

1) Tissue donor

Candidacy to selection method

Patients who meet the inclusion criteria will be considered candidates, and after confirming their eligibility based on medical history etc., those who have provided written informed consent will be enrolled as tissue donors.

2) Subjects (patients for transplantation)

Candidacy to selection method

Those who meet the selection criteria ① and ② will be selected as candidates, and those who have submitted written consent (first time) after confirming their eligibility based on medical history will be registered as cases. However, cell sheet transplantation will be performed only on those who have submitted written consent (second time) and who meet the selection criteria ③ and ④ based on preoperative arthroscopic findings.

8. Study methods

1) Phase of study: Clinical study

We carried out chondrocyte sheet transplantation prepared from patients' autologous cells during the clinical study for knee joint treatment using cell sheets with the issuance of the opinion of the Minister of Health, Labour and Welfare on October 3, 2011. The periosteum is not used when transplanting the cell sheet, which is also indicated for patients with osteoarthritis of the knee. To date, eight cases have patients the transplant with no serious adverse events, all of which followed a favorable progression with improved clinical symptoms. One year after the transplantation, the clinical study was completed. A safe and effective joint therapeutic effect has been obtained by chondrocyte sheet transplantation, and we are studying the joint therapeutic effect of cell sheet transplantation. In this study, we determined that allogeneic cell sheet transplantation is sufficiently safe based on the clinical study results of autologous cell sheet transplantation, as well as the results of safety assessment of allogeneic cells and those of allogeneic cell transplantation in animal experiments. Furthermore, we also determined the feasibility of a clinical study that uses cell sheets manufactured from isolated cells from surgical remains obtained from polydactyly patients (allogeneic cell sources). It is well known that allogeneic transplantation of cartilage is possible without immunosuppression and immune rejection. In other countries, allogeneic tissue, which is a chip of fresh cartilage tissue from young individuals, is being used clinically (Zimmer's DeNovo® NT). By using allogeneic chondrocytes, we can solve the issues concerning the use of the above-mentioned autologous cells. Furthermore, we believe that sufficient safety assessment can be performed by securing a sufficient number of cells with high cellular activity and assigning lot numbers. Since we will use cells isolated from tissues collected during surgery in the same facility, traceability will not be an issue.

We applied for a human stem cell clinical study because we believe that the use of a completely safe allogeneic cell sheet will result in a less invasive treatment and will have the same effect as an autologous cell sheet. With safety as the primary endpoint of this study, the efficacy of the treatment will be objectively assessed as a secondary endpoint, and various clinical evaluations will be performed to collect data on the treatment's effects.

2) Study design

As a treatment for cartilage defects in osteoarthritis, a single-arm open-label, uncontrolled, and comparative study will be conducted to confirm the safety and efficacy

of cartilage repair and restoration effects of allogeneic cell sheet transplantation.

3) Methods to minimize bias

This study is designed as an exploratory study with safety assessment as the primary endpoint, and it is a single-arm open-label, uncontrolled trial.

In this study, the age and BMI of the subjects, range of cartilage defect, defect region, number of cell sheet transplants, etc., are likely important factors in efficacy assessment and will be recorded as transplant patient summary in the case report form (transplantation).

To avoid bias toward efficacy evaluation, multiple physicians will evaluate the endpoints.

4) Method

This clinical study will use chondrocytes isolated from the cartilage tissue of the knuckles to be discarded during surgery in patients who do not have infectious diseases or serious complications other than polydactyly. Most eligible patients for tissue donation are approximately a year old, so sufficient time must be given to the family, which will act as the representative, to submit informed consent; samples will be collected after obtaining the consent form. We will select cells that passed the endotoxin test, mycoplasma test, virus test, and sterility test and select cells suitable for cell karyotype analysis, cell morphology observation, and stability test. The subjects will provide informed consent twice in total: prior to preoperative arthroscopy and prior to cell sheet transplantation. The clinical study will be carried out upon obtaining each of the consent forms. The degree of the cartilage lesion during arthroscopy will be evaluated. Thereafter, we will confirm if the subject meets the selection criteria. Once the target patient has been enrolled, cell sheet preparation will begin approximately 3 weeks before the transplant date. Polydactyly-derived tissues are transported from the operating room to the cell processing center, and the cells will be isolated and cryopreserved in the same room to evaluate the properties and safety of the cells. Once the target patient has been enrolled, cell sheet preparation will begin approximately 3 weeks before the date of transplantation. The cells will be seeded in a temperature-responsive culture dish to fabricate an allogeneic chondrocyte sheet. The fabricated allogeneic chondrocyte sheet (final product) will be transplanted to the region of cartilage injury in the patient on the planned day of transplantation surgery. Multiple sheets may be transplanted according to the size of the region of cartilage injury. If the region of cartilage injury is filled with unhealthy tissue, the tissue will be excised, the lesion will be washed, marrow stimulation will be performed, and a cell

sheet will be transplanted directly above the injured region. The allogeneic chondrocyte sheet will not be sutured to the surrounding tissue.

5) Study observation period and test items

<Hospitalization period>

Subjects will be hospitalized for 1 month, and postoperative rehabilitation and rest will be similar to those of subjects undergoing HTO alone.

-Content of rehabilitation-

The patient’s knee will be fixed with a plaster splint immediately after surgery until two weeks after surgery, and range of motion training will not be possible during this period.

Weight bearing:

・Complete non weight bearing immediately after surgery and up to 2 weeks

・One-third weight bearing from 3 weeks after partial weight bearing to 4 weeks after surgery

・One-half weight bearing from 4 to 5 weeks after surgery

・Full weight bearing 5 weeks after surgery

Range of motion training: starting after external fixation for 2 weeks after surgery

Muscle strength training with unloading: Isometric exercise from the day after surgery to 2 weeks after surgery

Exercise will be allowed after about 3 months. Since there are individual differences in recovery, this will be adjusted according to the subject’s condition.

Study observation period and examination items

Clinical study observation period: One year from the date of surgery

<Examination/observation items and timeline>

	Before transplantation			Inpatient (Transplant)	After 1 month	After 3 months	After 6 months	After 1 year	Discontinuation
	Outpatient	Inpatient (Test)	Outpatient						
Obtain informed consent	◎		◎						
Confirm eligibility	○	◎							
Regular surgery				◎					
Clinical evaluation	◎				◎	◎	◎	◎	
X-ray (lower limbs)	◎				◎	◎	◎	◎	
MRI	◎				◎	◎	◎	◎	
Arthroscopy		◎						◎	
Photoacoustic measurement		◎						◎	
Histological evaluation								◎	

Confirm adverse events*				◎	◎	◎	◎	◎	△

◎: Required　　○: Implemented when possible　　△: Implemented when necessary

*: Not required if no transplant

6) Overview of cells and specific cell processes (allogeneic chondrocyte sheets) used in this clinical study

<Cells used in this clinical study>

Name of cells: Polydactyly-derived chondrocyte cells

Cell donor selection method: The cell donor (substitute) shall be someone who has sufficient understanding of the clinical study, who is at an eligible age for polydactyly surgery, and meets the selection criteria of the cell donor.

Confirmation of eligibility of the cell donor: In addition to confirming the following medical history, eligibility will be determined based on experience of blood transfusion or transplantation.

・Infections caused by bacteria such as Treponema pallidum, gonorrhea, and Mycobacterium tuberculosis
・Sepsis and suspected sepsis
・Malignant tumor
・Serious metabolic endocrine disease
・Collagen disease or hematological disorders
・Liver disease
・Transmissible spongiform encephalopathy, suspected transmissible spongiform encephalopathy and dementia
・Specific hereditary diseases and family history related to the disease

Eligibility will be determined through interviews and tests to confirm the presence of infectious diseases

・HBV
・HCV
・HIV
・HTLV-1
・Parvovirus B19 (if necessary)

<Response in the event of doubts regarding cell safety>

In the event of doubts regarding cell safety, the physician performing regenerative medicine and so on shall promptly report to the research representative. The research representative shall report to the hospital director (manager), collect information on the

processing of cells and send to the manufacturing manager or quality control manager of the specific cell processing facility, check the manufacturing records and test records and so on and verify the safety. In addition, the patient's health status shall be checked, and if follow-up or treatment is required, it shall be promptly addressed.

<Method for manufacturing and quality control of specified cell products (chondrocyte sheets)>

Allogeneic chondrocyte sheets are prepared at the cell processing center (facility number: FC3150164) of the Central Medical Department, Tokai University Hospital, which is a facility licensed by the Ministry of Health, Labour and Welfare to manufacture specific cell processed products.

Name of a specified cell product: Allogeneic chondrocyte sheet

Content of manufacturing and quality control methods:

① Donor cell selection and allogeneic cell sheet fabrication

Donor cells will be isolated from cartilage tissue of the knuckles that are discarded at the time of surgery in patients with no serious complications other than infection and polydactyly. Compliance tests for quality and safety will be conducted, and cells confirmed to be compatible in all items will be cryopreserved for clinical research. The patient to undergo transplantation will be enrolled, and the frozen cell will be thawed and seeded from approximately 3 weeks before the date of transplantation. After the cells reach confluence, they will be seeded to a temperature-responsive culture dish, and the allogeneic chondrocyte sheet will be prepared.

② Storage methods of specified cell products, etc.

Donor cells: Cells that meet the compatibility test are cryopreserved.

Allogeneic chondrocyte sheets: They are not stored, but prepared according to the transplantation date. The administration period is 3 hours after packaging.

③ Analysis and testing method (quality control)

- Safety endpoints for donor cells

Acceptance tests: sterility test (direct method), mycoplasma test (gene detection and culture), endotoxin test (turbidimetry), and virus test A (Tokai University: gene detection method).

Culture process control: Sterility test (direct method), mycoplasma test (gene detection and culture methods), endotoxin test (turbidimetry), and virus-free test B (outsourced: gene detection method) are performed during the period up to the day before release. In virus-free assay A, HIV-1, HTLV-1, HCV, and HBV tests shall be

performed. In virus-free assay B, HIV-1, HIV-2, HTLV, HCV, HBV, Parvovirus B19, Epstein-Barr virus, cytomegalovirus, and West Nile virus tests shall be performed.
Compatibility test: Karyotype analysis (G-banding), stability test (array CGH), sterility test (direct method, Japanese Pharmacopoeia), mycoplasma test (gene detection and culture methods), and endotoxin test (turbidimetry) will be performed.

- Quality endpoints for donor cells

Culture process control: Cell count measurement (cell count and cell viability) is performed.
Compatibility test: Cell count (cell count and cell viability), morphology confirmation test (morphology and thickness), physical structure confirmation test (peeling test), and cartilage properties test (COLI and COLII) will be performed.

- Safety endpoints for allogeneic chondrocyte sheets

Culture process control: Sterility test (direct method), mycoplasma test (gene detection method and culture method), endotoxin test (turbidimetric method), and virus test A (Tokai University: gene detection method) will be performed during the period up to the day before release. Sterility test (direct method, Japanese Pharmacopoeia), and endotoxin test (turbidimetric method) will be performed on the day of release to check the safety of cell sheets used for transplantation.

- Quality endpoints for allogeneic chondrocyte sheets

Culture process control: Cell count measurement (cell count and cell viability), morphology confirmation test (morphology and thickness), and physical structure confirmation test (peeling test) will be performed for evaluation.

Use: Allogeneic chondrocyte sheets will be transplanted in combination with HTO.

<Expected benefits and disadvantages>

The restoration of cartilage defects in osteoarthritis of the knee using the original hyaline cartilage by allogeneic chondrocyte sheet transplantation is important from the functional aspect of the original joint function such as excellent viscoelasticity and wear resistance, which are the characteristics of hyaline cartilage. In order to maintain good knee joint function over the long term, it can be expected that one's joint can be preserved for a lifetime. Possible disadvantages include the possibility of infection and thrombosis that can be expected in HTO, the risk of using fetal bovine serum, the risk of using antibiotics, and the risk of infection in the production of specific cell processed products.

<Minimizing risks>

In the previous clinical study using the autologous cell sheet, no serious adverse events, such as major defects, and side effects occurred. However, if unexpected complications occur, appropriate measures must be promptly taken (such as administration of antibiotics, arthroscopic cleaning, and removal of transplanted cell sheets).

9. Postoperative tests, endpoints, and schedule

- Primary endpoint

<Safety assessment> Incidence of serious adverse events

Among the adverse events, the occurrence and incidence of serious adverse events will be confirmed, and the safety of treatment will be evaluated.

- Secondary endpoint

<Efficacy assessment>

1) Clinical assessment:

Tegner-Lysholm Knee Scoring Scale, Japanese Knee Injury and Osteoarthritis Outcome Score assessments will be performed preoperatively and at 1 month, 3 months, 6 months, and 1 year after surgery as a clinical evaluation criterion.

2) X-rays:

These will be used to assess the joint space, subchondral bone condition, and the progression of arthropathy. The progression of arthropathy will be objectively assessed using Kellgren-Lawrence grading scale before surgery and at 1 month, 3 months, 6 months, and 1 year after surgery.

3) MRI:

Changes in cartilage thickness and properties over time will be objectively assessed using Nelson MRI Grading preoperatively, and 1 month, 3 months, 6 months, and 1 year postoperatively.

4) Arthroscopy:

This will be used to evaluate the cartilage properties (color, hardness, and smoothness) and Outerbridge classification of the joint surface 1 year after surgery. If any injury or swelling of the joint occurs, the test shall be performed as appropriate to evaluate the condition of the cartilage.

5) Photoacoustic evaluation:

To quantitatively evaluate the viscoelastic properties of joint cartilage 1 year after surgery, we will evaluate the cartilage in the transplant region and surrounding cartilage under arthroscopy using an in-house functional diagnostic device. This is a functional evaluation method that has already been clinically applied at Tokai University Hospital under the approval of the Clinical Study Review Board of Tokai University School of Medicine.

6) Histological evaluation:

A part of the regenerated tissue will be submitted for biopsy at the time of arthroscopy, Safranin-O staining will be performed, and an objective histological evaluation will be performed using the Modified Mankin Score.

[Endpoints]

① Scores in clinical evaluation criteria at 1 year after surgery
② Scores in the X-ray evaluation criteria at 1 year after surgery
③ Scores in MRI evaluation criteria at 1 year after surgery
④ Viscoelastic evaluation with photoacoustic testing at 1 year after surgery
⑤ Scores in histological evaluation at 1 year after surgery

[Data management]

Regarding the subjects' data, information will be collected from the principal investigator and the investigator in charge of the subjects. The collaborators will compile a case report form, confirm it with the data manager, and then report it to the investigator. Data on safety and effectiveness will be reported regularly and to the Tokai University certified committee for regenerative medicine in accordance with the Regenerative Medicine Safety Assurance Act.

Data manager

Hiroyuki Kobayashi (Professor, Department of Clinical Pharmacology, Basic Medical Sciences, Tokai University School of Medicine)

10. Expected adverse events

A total of more than 20,000 cases of autologous cultured chondrocyte transplantation have been performed in other countries, and although there have been reports of postoperative infection, whose risk is at the same level as normal surgery, there have

been no reports of tumorigenesis. In addition, there have been reports of thickening and calcification of the transplanted periosteum in combination methods involving periosteum transplantation; however, periosteum will not be used in this study. In this clinical study, the only procedure performed on the subject in addition to the original surgery is the transplant of the cell sheet into the region of cartilage injury. It is well known that allogeneic transplantation of cartilage is possible without immunosuppression and immunorejection. In other countries, allogeneic tissue, which is a chip of fresh cartilage tissue from young individuals, is being clinically used (Zimmer's DeNovo® NT). Therefore, we believe that there is an extremely low possibility that the allogeneic chondrocyte sheet will be immunorejected. Furthermore, the safety of the allogeneic cell sheet will be confirmed by tumorigenicity test, stability test, and karyotype analysis. Moreover, various virus tests will be conducted multiple times to ensure sufficient safety. We believe that the risk of adverse events is at the same level as that of regular knee surgery under general anesthesia, and it is unlikely that their occurrence will be specific to this study.

11. Handling of adverse events

1) Symptoms or diseases

Any unfavorable or unintended sign, symptom, or disease occurring after surgery will be treated as an adverse event. If a complication worsens, it will also be handled as an adverse event. If the severity of the efficacy endpoint worsens, the event will not be treated as an adverse event.

2) Objective opinion

If abnormalities (normal→abnormal, abnormal→further abnormal) are shown by the final test compared with the test values* before the start of the clinical study, it will be treated as an adverse event. In addition, if there are missing test values* before the start of the clinical study and test values become abnormal after cell transplantation, it will be treated as an adverse event. However, if there are missing data, the value up to 30 days before the date of obtaining consent will be used as the reference value for judgment.

*: Test values performed during the observation period after obtaining consent (those performed multiple times should be close to the values at the start of the treatment period)

Regardless of the items specified or not specified in this study protocol, the adverse events during onset, during maximal deterioration, and during outcome determination,

as well as the data necessary for determining association will be stated in the medical record.

3) Recording and investigation of adverse events

In this clinical study, after the provision of regenerative medicine and so on as a general rule, regular outpatient visits and tests (such as clinical evaluation, X-ray, MRI examination, and arthroscopy) for 5 years or more will be carried out in outpatient clinics, similar to postoperative follow-up of ordinary surgical therapy. In addition to verifying the effects of this regenerative medicine and so on we will confirm the presence or absence of diseases and so on caused by the provision of this regenerative medicine and so on and grasp and follow up the occurrence of diseases, etc.

If an adverse event occurs, its symptoms or disease, content of objective findings, date of onset, degree, severity, presence or absence of treatment and its content, outcome and its determination date, and relevance to this clinical study with reasons will be stated in the medical records. If the name of the disease is stated, the symptoms associated with the disease will not be noted as an adverse event.

If adverse events are observed in the symptoms or diseases observed during the treatment period or objective findings, regardless of whether there is a causal relationship with this clinical study, in principle, follow-up will be conducted until the condition stabilizes or recovers to a level that cannot be considered as an adverse event. However, this does not apply if the investigator confirms that the patient has recovered. In that case, the grounds for determining recovery shall be stated in the medical record. If irreversible adverse events are observed due to organic disorders (including cerebral infarction and myocardial infarction), follow-up will be conducted until the symptoms stabilize or resolve.

4) Classification of adverse events

The severity of adverse events will be classified according to the following criteria:

① Mild: The adverse event does not impair the patient’s daily life

② Moderate: The adverse event impairs daily life, but the patient is still able to perform activities

③ Advanced: The adverse event greatly impairs the patient’s daily life

The outcome of adverse events will be classified according to the following criteria:

① Recovered: Normalized or recovered to a level that cannot be considered as an adverse event

② Continuous recovery: Those who have not recovered at that time

③ Unknown (deceased): Unknown outcome due to patient death

5) Determining the association between adverse events and this clinical study

The association of adverse events with this clinical study will be determined according to the following criteria, taking into account the condition of the subject, the time relation to treatment, and other factors.

① Obviously associated

② Possibly associated

③ May be associated

④ Not associated

Adverse events that fall under ① to ③ are considered as adverse events for which association to this clinical study cannot be ruled out, and adverse events that fall under ④ are considered as adverse events for which the association with this study can be ruled out.

6) Serious adverse events

If serious adverse events occur during the treatment period, whether associated with the clinical study or not, the principal investigator or the investigators will take appropriate measures immediately for the subject. The investigator will promptly report to the head of the hospital. In response to the report, the implementation regulations will be followed. In addition, even if this clinical study has fewer than 10 cases, the study will be discontinued.

[Serious adverse events]

1) Death

2) Potentially fatal cases

3) Cases requiring admission to a medical institution or prolongation of hospital stay for treatment

4) Disorder

5) Cases that might result in disability

6) Serious cases

7) Any congenital disease or anomaly in the offspring of a treated patient.

7) Reporting of diseases, etc.

In the event of a disease, disability, death, or infectious disease suspected to be due to the provision of regenerative medicine, the principal investigator shall promptly report to the hospital director.

The providing institution manager will report diseases and so on to the certified committee for regenerative medicine and the Minister of Health, Labour and Welfare, in accordance with Ministerial Ordinance No. 110 (Articles 35 and 36). Furthermore, in the event of a situation that may have a significant impact on ensuring the safety of the specified processed cell product, the product manufacturer shall report the serious situation in accordance with Article 107 of the Ministerial Ordinance.

8) Measures to understand information

The presence of diseases and so on will be confirmed in the outpatient clinic, and information such as health status will be grasped. When a disease and so on suspected to be caused by the provision of this regenerative medicine and so on occurs, a certain follow-up period will be provided to grasp information on the disease, etc.

We will establish a system that allows us to obtain the contact information of those who have received this regenerative medicine and so on and to grasp the outbreak of illness, etc. Necessary information will be grasped from the records such as the electronic medical record system of the hospital.

9) Providing new information

If new information on the safety of this clinical study is obtained, the practitioner will promptly submit a written report to the hospital director, dean of the school of medicine, principal investigator and investigators of the clinical study. The principal investigator and investigators will provide additional explanation to the subject and revise the explanatory document and informed consent form as necessary.

12. Medical fees and compensation

1) Medical fees

All costs related to cell sheet transplantation are paid from the research funds of Tokai University School of Medicine, with no out-of-pocket payments from the patient.

2) Compensation

If the clinical study results in compensation or liability for health damage to subjects, insurance for clinical studies, such as regenerative medicine, and compensation is possible within the scope of the compensation.

13. Statistical considerations

1) Target enrollments and rationale

Based on the results of clinical studies conducted to date, we estimated a target response rate of 75% and a threshold response rate of 25%. Assuming that the result exceeds the threshold response rate, the required number of cases calculated with α=0.05 and 1-β (detection power)=0.9 is eight cases. Considering the feasibility of the study, 10 cases were set.

2) Analysis set

Cell sheet transplantation is performed.

3) Analysis items and methods

① Analysis items:

The secondary endpoint, the Japanese Knee Injury and Osteoarthritis Outcome Sco re (J-KOOS) score 1 year after surgery, will be evaluated from the perspective of i mproving clinical symptoms.

② Analysis methods:

The amount of change in J-KOOS (value 1 year after surgery – preoperative valu e) in the target population will be analyzed by one-way analysis of variance and multiple comparison test (post hoc Holm-Bonferroni).

<Handling of missing data or dropouts>

If there are missing data 1 year after surgery and dropouts less than 1 year after surgery (evaluation points), the final J-KOOS score from the scores obtained 1 month, 3 months, and 6 months after surgery will be used for evaluation.

4) Interim analysis

Since the target enrollment is as small as 10 and the endpoint is 1 year after surgery, no interim analysis is planned. However, for cases with poor improvement of JKOOS as an efficacy evaluation, the assessment will be carried out each time.

5) Changes in the original statistical analysis plan

In the event of changes in the original statistical analysis plan, the protocol and statistical analysis plan will be revised, and the changes will be explained in the summary report. An application for changes to the certified committee for regenerative medicine will be submitted, and after receiving the opinion of the committee, the Minister of Health, Labour and Welfare will be notified via the director of the regional welfare bureau.

6) Statistician

Hiroyuki Kobayashi (Professor, Department of Clinical Pharmacology, Internal Medicine Science, Tokai University School of Medicine)

14. Disclosure of study information and publication of results

Information on the study will be uploaded in the public database (https://jrct.niph.go.jp) of the Japan Registry of Clinical Trials (jRCT) developed by the Ministry of Health, Labour and Welfare, and updated as appropriate. In addition, information on the study will be released on the project website of the research representative.

The results obtained in this study may be published in academic conferences, papers, etc. At the time of presentation, the subject's confidentiality (privacy) will be preserved. Furthermore, evaluation and analysis will be performed with the endpoint at one year of the observation period. A summary report will be submitted to the Ministry of Health, Labour and Welfare, and will be published on jRCT.

15. Use of samples and information in new research

The samples and information obtained in this study are extremely valuable and can only be obtained through surgery. Specimens and collected data that could not be used as surplus or for transplantation may be anonymized and used for other research for the development of medicine with the consent of the subject or the surrogate (see consent form). It may also be discarded without being used for transplantation.

16. Management of samples and information (storage and disposal)

1) Sample storage, storage period, and disposal

Samples, such as a part of cells, and a part of a specific processed cell product will be stored for 10 years by consignment to a storage room or reagent adjustment room affiliated with the cell processing facility, cell therapy archive, etc. After the storage period, the samples will be processed to prevent identification of the individual and then disposed of as medical waste in accordance with waste management regulations.

2) Information management, storage period, and disposal

The handling, management, storage, and storage period of the records related to the test are as follows.

Records related to regenerative medicine, etc.: Electronic data will be managed and stored in electronic medical records and hard disks dedicated to clinical research, and

paper-based original materials, case report forms, and materials related to tests will be stored in lockers with keys. The documents will be stored for 30 years in accordance with the Act on Securing Safety of Regenerative Medicine.

17. Study funding and conflicts of interest of investigators, etc.

This research will be carried out with research funding from the Japan Agency for Medical Research and Development. In addition, the research representative has received research funding from CellSeed Co., Ltd., and is conducting joint research such as optimizing the cell sheet manufacturing method and improving the culture equipment. However, since the funds provided will not be used for this clinical study, the transparency of the study implementation and the reliability of the results will not be compromised. In addition, the research representative of this clinical study will report to the Conflict-of-Interest Management Committee of the University and undergo review. The Institutional Review Board (certified committee for regenerative medicine) has recognized the appropriateness of the implementation of this study.

18. Response to inquiries from subjects and related individuals

○Clinical Study Coordinator: Yuko Chiba (job title: nurse), Contact information; 463-93-1121

○Patient Support Center: Information desk: 1st floor of hospital, general counseling room, contact information: 0463-93-1121

19. Response to the provision of medical care to subjects after study

As a general rule, follow-up will be carried out for at least 5 years in outpatient clinics, as in the case of postoperative follow-up of regular surgery.

20. Monitoring and audit

Monitoring will be carried out by people in charge designated by the principal investigator from among the investigators and collaborators in this research. As for the implementation procedure, the test implementation procedure will be confirmed, documents will be viewed, and the storage status will be confirmed by on-site monitoring according to the procedure manual. After confirmation of the monitoring results from the monitoring manager, a report will be submitted to the principal investigator. When an audit of medical institutions and so on is conducted, the principal investigator shall cooperate in the source document verification, etc.

Monitoring manager

Miko Shimazawa (Professor, Department of Clinical Pharmacology, Basic Medical Science, Tokai University School of Medicine)

Monitoring staff

Eriko Toyota (Postdoctoral Fellow, Department of Orthopedic Surgery, Surgical Science, Tokai University School of Medicine)

21. References

1. Masato Sato. Knee cartilage restoration treatment with cell sheets. Pharma Medica 31(4), 15-19, 2013.
2. Ebihara G, Sato M, Yamato M, Mitani G, Kutsuna T, Nagai T, Ito S, Ukai T, Kobayashi M, Kokubo M, Okano T, Mochida J. Cartilage repair in transplanted scaffold-free chondrocyte sheets using a minipig model. Biomaterials 33(15), 3846-3851, 2012.
3. Ito S, Sato M, Yamato M, Mitani G, Kutsuna T, Nagai T, Ukai T, Kobayashi M, Kokubo M, Okano T, Mochida J. Repair of articular cartilage defect with layered chondrocyte sheets and cultured synovial cells. Biomaterials 33(21), 5278-5286, 2012.
4. Hamahashi K, Sato M, Yamato M, Kokubo M, Mitani G, Ito S, Nagai T, Ebihara G, Kutsuna T, Okano T, Mochida J. Studies of the humoral factors produced by layered chondrocyte sheets. J Tissue Eng Regen Med, 2012. Published online in Wiley Online Library.
5. Kokubo M, Sato M, Yamato M, Mitani G, Kutsuna T, Ebihara G, Okano T, Mochida J. Characterization of chondrocyte sheets prepared using a co-culture method with temperature-responsive culture inserts. J Tissue Eng Regen Med (2013) Published online in Wiley Online Library.
6. Takaku Y, Murai K, Ukai T, Ito S, Kokubo M, Satoh M, Kobayashi E, Yamato M, Okano T, Takeuchi M, Mochida J, Sato M. In vivo cell tracking by bioluminescence imaging after transplantation of bioengineered cell sheets to the knee joint. Biomaterials 35(7), 2199-2206, 2014.
7. Maehara M, Sato M, Watanabe M, Matsunari H, Kokubo M, Kanai T, Sato M, Matsumura K, Hyon SH, Yokoyama M, Mochida J, Nagashima H. Development of a novel vitrification method for chondrocyte sheets. BMC Biotechnol 13(58), 2013.
8. Kutsuna T, Sato M, Ishihara M, Furukawa KS, Nagai T, Kikuchi M, Ushida T, Mochida J. Noninvasive evaluation of tissue-engineered cartilage with time-resolved laser-induced fluorescence spectroscopy. Tissue Eng Part C Methods

16(3), 365-373, 2010.
9. Kaneshiro N, Sato M, Ishihara M, Mitani G, Sakai H, Mochida J. Bioengineered chondrocyte sheets may be potentially useful for the treatment of partial thickness defects of articular cartilage. Biochem Biophys Res Commun 349 (2), 723-731, 2006.
10. Mitani G, Sato M, Lee JIK, Kaneshiro N, Ishihara M, Ota N, Kokubo M, Sakai H, Kikuchi T, Mochida J. The properties of bioengineered chondrocyte sheets for cartilage regeneration. BMC Biotechnol 9(17), 2009.
