# Supplementary material for: Polydactyly-derived allogeneic chondrocyte cell-sheet transplantation with high tibial osteotomy as regenerative therapy for knee osteoarthritis
Source: NPJ Regen Med. 2022 Dec 16;7:71. doi: 10.1038/s41536-022-00272-1 (PMC9755241; doi:10.1038/s41536-022-00272-1)
Supplement: Supplementary file 2 — Supplementary Materials [file 41536_2022_272_MOESM2_ESM.pdf]

Supplementary Table1 Source data for Fig 3a

PatientID	time	KOOS Symptom	KOOS Pain	KOOS ADL	KOOS Sport/Re	KOOS QOL	total KOOS
001	Preop.	78.57	69.44	72.06	55.00	31.25	61.26
001	1M	60.71	77.78	58.82	10.00	37.50	48.96
001	3M	96.43	91.67	98.53	35.00	68.75	78.07
001	6M	100.00	94.44	98.53	65.00	62.50	84.09
001	12M	100.00	94.44	100.00	90.00	68.75	90.64
002	Preop.	50.00	41.67	67.65	15.00	12.50	37.36
002	1M	82.14	75.00	82.35	0.00	50.00	57.90
002	3M	89.29	77.78	92.65	30.00	56.25	69.19
002	6M	82.14	86.11	94.12	35.00	50.00	69.47
002	12M	89.29	91.67	94.12	45.00	62.50	76.51
003	Preop.	57.14	47.22	54.41	20.00	6.25	37.01
003	1M	71.43	63.89	67.65	0.00	18.75	44.34
003	3M	82.14	69.44	85.29	0.00	31.25	53.63
003	6M	89.29	91.67	98.53	20.00	43.75	68.65
003	12M	85.71	88.89	98.53	35.00	62.50	74.13
004	Preop.	71.43	52.78	88.24	15.00	6.25	46.74
004	1M	75.00	83.33	88.24	0.00	31.25	55.56
004	3M	82.14	83.33	95.59	0.00	62.50	64.71
004	6M	85.71	91.67	92.65	0.00	43.75	62.76
004	12M	89.29	88.89	83.82	65.00	75.00	80.40
005	Preop.	35.71	13.89	44.12	0.00	6.25	19.99
005	1M	75.00	63.89	82.35	0.00	18.75	48.00
005	3M	53.57	75.00	80.88	0.00	25.00	46.89
005	6M	75.00	77.78	91.18	15.00	43.75	60.54
005	12M	64.29	80.56	85.29	35.00	43.75	61.78
006	Preop.	57.14	36.11	58.82	10.00	31.25	38.67
006	1M	50.00	50.00	36.76	0.00	6.25	28.60
006	3M	71.43	50.00	72.06	0.00	25.00	43.70
006	6M	75.00	72.22	66.18	0.00	50.00	52.68
006	12M	67.86	69.44	72.06	40.00	62.50	62.37
007	Preop.	39.29	36.11	55.88	10.00	0.00	28.26
007	1M	67.86	80.56	76.47	0.00	43.75	53.73
007	3M	64.29	72.22	75.00	10.00	50.00	54.30
007	6M	75.00	77.78	86.76	20.00	62.50	64.41
007	12M	57.14	63.89	80.88	0.00	37.50	47.88
008	Preop.	50.00	61.11	48.53	15.00	25.00	39.93
008	1M	57.14	63.89	70.59	40.00	37.50	53.82
008	3M	78.57	77.78	79.41	40.00	62.50	67.65
008	6M	96.43	94.44	97.06	85.00	87.50	92.09
008	12M	100.00	94.44	100.00	95.00	93.75	96.64
009	Preop.	75.00	69.44	80.88	50.00	25.00	60.07
009	1M	53.57	66.67	50.00	0.00	18.75	37.80
009	3M	57.14	61.11	76.47	10.00	31.25	47.19
009	6M	46.43	58.33	86.76	10.00	31.25	46.56
009	12M	92.86	100.00	100.00	35.00	75.00	80.57
010	Preop.	32.14	52.78	60.29	0.00	31.25	35.29
010	1M	32.14	33.33	47.06	0.00	0.00	22.51
010	3M	50.00	50.00	79.41	15.00	18.75	42.63
010	6M	71.43	72.22	97.06	30.00	25.00	59.14
010	12M	89.29	88.89	98.53	10.00	43.75	66.09
Average	12M	83.57	86.11	91.32	45.00	62.50	
Average	6M	79.64	81.67	90.88	28.00	50.00	
Average	3M	72.50	70.83	83.53	14.00	43.13	
Average	1M	62.50	65.83	66.03	5.00	26.25	
Average	Preop.	54.64	48.06	63.09	19.00	17.50	

Supplementary Table2　Source data for Fig 3b

	Patient1	Patient2	Patient3	Patient4	Patient5	Patient6	Patient7	Patient8	Patient9	Patient10	Ave	SD
Preop.	50	37	62	42	11	43	28	34	43	51	40.1	14
1M	59	58	41	66	38	33	65	46	39	41	48.6	12
3M	96	78	73	82	41	44	44	83	63	29	63.3	22
6M	100	88	95	77	61	49	56	91	66	84	76.7	18
12M	100	85	84	82	56	70	79	95	89	87	82.7	12

Supplementary Table 3. Probes used in qPCR

Gene Symbol	Assay ID	Reference Sequence
ACAN	Hs00153936_m1	NM_013227.3; NM_001135.3
ACKR4	Hs00664347_s1	NM_016557.3; NM_178445.2
ACTB	Hs01060665_g1	NM_001101.3
ADAMTS5	Hs00199841_m1	NM_007038.3
BMP6	Hs01099599_gH	NM_001718.4
CCN2	Hs01026927_g1	NM_001901.2
CD44	Hs01075861_m1	NM_000610.3; NM_001202555.1; NM_001202556.1; NM_001001392.1; NM_001001391.1; NM_001001390.1; NM_001001389.1
CHI3L1	Hs01072228_m1	NM_001276.2
COL11A2	Hs00899185_g1	NM_080681.2; NM_080679.2; NM_080680.2
COL1A1	Hs01076775_g1	NM_000088.3
COL1A2	Hs01028971_m1	NM_000089.3
COL27A1	Hs00259829_m1	NM_032888.2
COL2A1	Hs01060356_g1	NM_033150.2; NM_001844.4
COL6A1	Hs01095599_g1	NM_001848.2
COMP	Hs01572837_g1	NM_000095.2
CSGALNACT1	Hs00218054_m1	NM_018371.4; NM_001130518.1
CXCL6	Hs00605742_g1	NM_002993.3
ECM2	Hs00154821_m1	NM_001197296.1; NM_001393.3; NM_001197295.1
ECRG4	Hs00260897_m1	NM_032411.2
ESM1	Hs00199831_m1	NM_007036.4
FN1	Hs01549970_g1	NM_212482.1; NM_002026.2; NM_212476.1; NM_212478.1; NM_212474.1
G0S2	Hs00274783_s1	NM_015714.3
GAPDH	Hs02758991_g1	NM_002046.4; NM_001256799.1
GDF5	Hs00167060_m1	NM_000557.2
GREM1	Hs01879841_s1	NM_001191322.1; NM_001191323.1; NM_013372.6
ITGA10	Hs01006923_g1	NM_003637.3
LAMB3	Hs00165078_m1	NM_000228.2; NM_001017402.1; NM_001127641.1
LUM	Hs00929860_m1	NM_002345.3
MATN2	Hs01051833_g1	NM_030583.2; NM_002380.3
MCAM	Hs00174838_m1	NM_006500.2
MGP	Hs00969490_m1	NM_000900.3; NM_001190839.1
MIA	Hs00197954_m1	NM_006533.3; NM_001202553.1
MMP13	Hs00233992_m1	NM_002427.3
MMP3	Hs00968305_m1	NM_002422.3
PTGS2	Hs00153133_m1	NM_000963.2
RUNX2	Hs00231692_m1	NM_001015051.3; NM_001278478.1; NM_001024630.3
SERPINF1	Hs01106934_m1	NM_002615.5
SLC14A1	Hs00998199_m1	NM_001128588.3; NM_001146036.2; NM_001146037.1; NM_001308278.1; NM_001308279.1; NM_015865.6
SNX19	Hs01040307_g1	NM_014758.2
SOX5	Hs00753050_s1	NM_001261414.1; NM_006940.4; NM_001261415.1; NM_178010.2; NM_152989.3

SOX6	Hs00264525_m1	NM_033326.3; NM_017508.2; NM_001145819.1; NM_001145811.1
SOX9	Hs01001343_g1	NM_000346.3
TGFB1	Hs00998133_m1	NM_000660.4
TIMP1	Hs00171558_m1	NM_003254.2
VEGFA	Hs00900055_m1	NM_001025366.2; NM_001025367.2; NM_001025368.2; NM_001025369.2; NM_001025370.2; NM_001033756.2; NM_001171622.1; NM_001171623.1; NM_001171624.1; NM_001171625.1; NM_001171626.1; NM_001171627.1; NM_001171628.1; NM_001171629.1; NM_001171630.1; NM_001204384.1; NM_001204385.1; NM_001287044.1; NM_001317010.1; NM_003376.5

Supplementary Table 4 Source data for Fig 3c

KOOS (Pain)		94	92	89	89	81	69	64	94	100	89			
dCt	Symbol	1	2	3	4	5	6	7	8	9	10	ave	Weight	Corr
	CCN2(CTGF)	−2.9656	−2.99	−2.0329	−3.0106	−2.9905	−2.9707	−2	−3.94	−2.82	−2.82	−2.85403	40	−0.44
	COL1A1	4.0431	1.06	2.9845	−0.0128	−0.9918	1.002	2.97	2.87	1.97	1.97	1.7865	34	0.17
	COL1A2	2.047	−0.03	2.0213	−0.9818	−1.9642	0.0124	1.87	2.06	2.01	2.01	0.90547	47	0.25
	COL2A1	−11.0047	−10.86	−9.0389	−10.0075	−9.9986	−10.9984	−9.08	−6.98	−8.02	−8.02	−9.40081	43	0.30
	COL6A1	−2.9728	−3.88	−4.0028	−0.994	−2.9988	−0.9797	−3.13	−4.04	−4.06	−4.06	−3.11181	56	−0.47
	COMP	1.0275	0.11	1.0451	−2.0063	−2.9736	−2.0173	0.98	−1.08	−0.87	−0.87	−0.66546	43	0.08
	ACAN	−7.0121	−5.91	−6.0345	−5.0147	−6.9973	−5.0141	−6.14	−7.09	−6.95	−6.95	−6.31127	48	−0.45
	ECM2	−8.9025	−11.01	−9.0091	−9.9383	−11.9366	−9.9988	−8.11	−9.06	−8.83	−8.83	−9.56253	35	0.02
	FN1	3.0907	1.13	3.0251	0.1634	−0.9103	1.0321	4.21	2	2.26	2.26	1.8261	44	−0.07
	SNX19	−8.99	−8.91	−7.965	−7.9974	−8.9979	−7.9651	−7.96	−8	−7.95	−7.95	−8.26854	21	−0.17
	LUM	2.0223	−0.99	1.9891	−1.9138	−2.8662	−1.9733	0.9	−0.02	0.24	0.24	−0.23719	48	0.26
	MATN2	−4.7702	−7.87	−4.8325	−7.9524	−8.0255	−7.877	−5.1	−4.99	−4.98	−4.98	−6.13776	51	0.29
	MGP	−8.1105	−11.13	−6.9535	−9.8372	−11.7893	−9.8377	−7.64	−6.92	−6.91	−6.91	−8.60382	37	0.26
	MMP3	−8.8942	−7.78	−6.9397	−8.9081	−8.9156	−7.8888	−8.14	−4.96	−4.76	−4.76	−7.19464	41	0.44
	MMP13	−2.8611	−2.89	−1.9182	−3.9949	−4.9589	−3.9403	−2.12	−1.03	0.05	0.05	−2.36134	54	0.43
	SERPINF1	−8.8206	−7.82	−7.9639	−6.9758	−7.9654	−5.9915	−6	−6	−5.88	−5.88	−6.92972	28	−0.25
	SOX6	−7.9499	−8.81	−8.0079	−7.9978	−8.9731	−7.9962	−7.94	−8.9	−9	−9	−8.45749	54	−0.46
	PTGS2	−7.9786	−6.89	−6.0181	−5.9539	−6.9557	−5.0045	−7.06	−9	−8.03	−8.03	−7.09208	76	−0.55
	ACTB	−1.9955	−0.84	−1.0256	−0.0322	−1.9757	0.0006	−1.07	−2.12	−2.07	−2.07	−1.31984	49	−0.48
	SOX9	−9.0044	−8.92	−9.9599	−6.9985	−6.9955	−6.0045	−9.96	−11.06	−10.97	−10.97	−9.08428	56	−0.45
	TGFB1	−1.9908	−2.93	−3.001	−4.0104	−4.9872	−4.0138	−3.11	−3.05	−3.04	−3.04	−3.31732	64	0.42
	TIMP1	−0.0072	−0.87	0.9753	−1.0185	−2.9772	−0.9994	1.08	0	−0.02	−0.02	−0.3857	38	0.03
	VEGFA	−6.0036	−5.88	−5.0144	−5.0125	−5.9469	−5.0116	−5.06	−6.07	−5.04	−5.04	−5.4079	31	−0.32
	MIA	−5.0067	−4.92	−4.0445	−6.0268	−7.0055	−5.9739	−2.12	−5.05	−5.05	−5.05	−5.02474	69	−0.28
	ITGA10	−7.9249	−6.91	−7.0019	−5.9954	−6.9785	−5.987	−6.14	−6.01	−6.93	−6.93	−6.68077	44	−0.48
	COL27A1	−6.8312	−6.93	−6.8749	−6.9523	−7.9277	−6.9453	−7.07	−8.02	−7.04	−7.04	−7.16314	20	−0.02
	CD44	−1.9827	−2.9	−1.9881	−1.9585	−2.9398	−2.9542	−1.06	−1.97	−1.05	−1.05	−1.98533	53	0.15
	GDF5	−10.0337	−9.72177	−9.29114	−8.89407	−9.52517	−8.94083	−8.24564	−8.95951	−9.32106	−9.32106	−9.22539	63	−0.64
	CHI3L1	−3.07629	−1.04436	−2.15193	−0.68843	−1.56021	−0.80631	−0.84734	−0.20991	−0.39275	−0.39275	−1.11703	20	−0.07
	SLC14A1	−6.18985	−5.73932	−6.18322	−6.00298	−6.05102	−6.10998	−5.95646	−6.42848	−6.79221	−6.79221	−6.22457	39	−0.45
	ACKR4	−7.09358	−5.92536	−6.5111	−4.55019	−4.79475	−4.57069	−4.833	−4.0784	−4.40055	−4.40055	−5.11582	28	−0.19
	ESM1	−5.78454	−5.6019	−5.00078	−6.31876	−7.44041	−5.99155	−5.18054	−7.3377	−8.14868	−8.14868	−6.49535	35	−0.42
	GREM1	−1.62234	−1.41603	−1.38249	−1.07265	−0.88165	−1.2348	−1.22787	−1.19476	−1.37212	−1.37212	−1.27768	35	−0.39
	LAMB3	−12.4681	−12.1783	−12.6957	−12.2737	−12.5432	−11.8793	−11.3977	−12.7199	−13.768	−13.768	−12.5692	80	−0.72
	ECRG4(C2orf40)	−6.43748	−6.68077	−6.95285	−6.95071	−6.70694	−6.60886	−6.71116	−7.33705	−7.87219	−7.87219	−7.01302	57	−0.47
	G0S2	−9.87822	−8.80164	−9.41247	−8.23331	−7.91401	−8.03359	−7.91659	−8.43953	−9.4839	−9.4839	−8.75971	91	−0.71
Marker		1	2	3	4	5	6	7	8	9	10			Corr
Normalized dCt	**PTGS2**	−0.89	0.20	1.07	1.14	0.14	2.09	0.03	−1.91	−0.94	−0.94			−0.55
	**TGFB1**	1.33	0.39	0.32	−0.69	−1.67	−0.70	0.21	0.27	0.28	0.28			0.42
	**MIA**	0.02	0.10	0.98	−1.00	−1.98	−0.95	2.90	−0.03	−0.03	−0.03			−0.28
	**G0S2**	−1.12	−0.04	−0.65	0.53	0.85	0.73	0.84	0.32	−0.72	−0.72			−0.71
	GeneScore	3.31	0.12	−1.09	−1.36	−0.67	−2.56	−3.57	1.88	1.96	1.96			0.85

Supplementary Table 5 Source data for Fig 3d

Lysholm score		100	85	84	82	56	70	79	95	89	87			
dCt	Symbol	1	2	3	4	5	6	7	8	9	10	ave	Weight	Corr
	CCN2(CTGF)	−2.9656	−2.99	−2.0329	−3.0106	−2.9905	−2.9707	−2	−3.94	−2.82	−2.82	−2.85403	11	−0.20
	COL1A1	4.0431	1.06	2.9845	−0.0128	−0.9918	1.002	2.97	2.87	1.97	1.97	1.7865	83	0.78
	COL1A2	2.047	−0.03	2.0213	−0.9818	−1.9642	0.0124	1.87	2.06	2.01	2.01	0.90547	85	0.77
	COL2A1	−11.0047	−10.86	−9.0389	−10.0075	−9.9986	−10.9984	−9.08	−6.98	−8.02	−8.02	−9.40081	42	0.31
	COL6A1	−2.9728	−3.88	−4.0028	−0.994	−2.9988	−0.9797	−3.13	−4.04	−4.06	−4.06	−3.11181	64	−0.39
	COMP	1.0275	0.11	1.0451	−2.0063	−2.9736	−2.0173	0.98	−1.08	−0.87	−0.87	−0.66546	73	0.62
	ACAN	−7.0121	−5.91	−6.0345	−5.0147	−6.9973	−5.0141	−6.14	−7.09	−6.95	−6.95	−6.31127	60	−0.29
	ECM2	−8.9025	−11.01	−9.0091	−9.9383	−11.9366	−9.9988	−8.11	−9.06	−8.83	−8.83	−9.56253	60	0.66
	FN1	3.0907	1.13	3.0251	0.1634	−0.9103	1.0321	4.21	2	2.26	2.26	1.8261	69	0.61
	SNX19	−8.99	−8.91	−7.965	−7.9974	−8.9979	−7.9651	−7.96	−8	−7.95	−7.95	−8.26854	54	0.14
	LUM	2.0223	−0.99	1.9891	−1.9138	−2.8662	−1.9733	0.9	−0.02	0.24	0.24	−0.23719	83	0.73
	MATN2	−4.7702	−7.87	−4.8325	−7.9524	−8.0255	−7.877	−5.1	−4.99	−4.98	−4.98	−6.13776	85	0.68
	MGP	−8.1105	−11.13	−6.9535	−9.8372	−11.7893	−9.8377	−7.64	−6.92	−6.91	−6.91	−8.60382	70	0.67
	MMP3	−8.8942	−7.78	−6.9397	−8.9081	−8.9156	−7.8888	−8.14	−4.96	−4.76	−4.76	−7.19464	51	0.42
	MMP13	−2.8611	−2.89	−1.9182	−3.9949	−4.9589	−3.9403	−2.12	−1.03	0.05	0.05	−2.36134	70	0.64
	SERPINF1	−8.8206	−7.82	−7.9639	−6.9758	−7.9654	−5.9915	−6	−6	−5.88	−5.88	−6.92972	43	−0.01
	SOX6	−7.9499	−8.81	−8.0079	−7.9978	−8.9731	−7.9962	−7.94	−8.9	−9	−9	−8.45749	39	0.05
	PTGS2	−7.9786	−6.89	−6.0181	−5.9539	−6.9557	−5.0045	−7.06	−9	−8.03	−8.03	−7.09208	78	−0.58
	ACTB	−1.9955	−0.84	−1.0256	−0.0322	−1.9757	0.0006	−1.07	−2.12	−2.07	−2.07	−1.31984	60	−0.31
	SOX9	−9.0044	−8.92	−9.9599	−6.9985	−6.9955	−6.0045	−9.96	−11.06	−10.97	−10.97	−9.08428	94	−0.66
	TGFB1	−1.9908	−2.93	−3.001	−4.0104	−4.9872	−4.0138	−3.11	−3.05	−3.04	−3.04	−3.31732	97	0.91
	TIMP1	−0.0072	−0.87	0.9753	−1.0185	−2.9772	−0.9994	1.08	0	−0.02	−0.02	−0.3857	57	0.67
	VEGFA	−6.0036	−5.88	−5.0144	−5.0125	−5.9469	−5.0116	−5.06	−6.07	−5.04	−5.04	−5.4079	52	−0.13
	MIA	−5.0067	−4.92	−4.0445	−6.0268	−7.0055	−5.9739	−2.12	−5.05	−5.05	−5.05	−5.02474	42	0.38
	ITGA10	−7.9249	−6.91	−7.0019	−5.9954	−6.9785	−5.987	−6.14	−6.01	−6.93	−6.93	−6.68077	49	−0.28
	COL27A1	−6.8312	−6.93	−6.8749	−6.9523	−7.9277	−6.9453	−7.07	−8.02	−7.04	−7.04	−7.16314	46	0.30
	CD44	−1.9827	−2.9	−1.9881	−1.9585	−2.9398	−2.9542	−1.06	−1.97	−1.05	−1.05	−1.98533	63	0.49
	GDF5	−10.0337	−9.72177	−9.29114	−8.89407	−9.52517	−8.94083	−8.24564	−8.95951	−9.32106	−9.32106	−9.22539	39	−0.22
	CHI3L1	−3.07629	−1.04436	−2.15193	−0.68843	−1.56021	−0.80631	−0.84734	−0.20991	−0.39275	−0.39275	−1.11703	51	−0.10
	SLC14A1	−6.18985	−5.73932	−6.18322	−6.00298	−6.05102	−6.10998	−5.95646	−6.42848	−6.79221	−6.79221	−6.22457	27	−0.36
	ACKR4	−7.09358	−5.92536	−6.5111	−4.55019	−4.79475	−4.57069	−4.833	−4.0784	−4.40055	−4.40055	−5.11582	43	−0.30
	ESM1	−5.78454	−5.6019	−5.00078	−6.31876	−7.44041	−5.99155	−5.18054	−7.3377	−8.14868	−8.14868	−6.49535	27	0.02
	GREM1	−1.62234	−1.41603	−1.38249	−1.07265	−0.88165	−1.2348	−1.22787	−1.19476	−1.37212	−1.37212	−1.27768	77	−0.78
	LAMB3	−12.4681	−12.1783	−12.6957	−12.2737	−12.5432	−11.8793	−11.3977	−12.7199	−13.768	−13.768	−12.5692	32	−0.31
	ECRG4(C2orf40)	−6.43748	−6.68077	−6.95285	−6.95071	−6.70694	−6.60886	−6.71116	−7.33705	−7.87219	−7.87219	−7.01302	51	−0.29
	G0S2	−9.87822	−8.80164	−9.41247	−8.23331	−7.91401	−8.03359	−7.91659	−8.43953	−9.4839	−9.4839	−8.75971	87	−0.70
Marker	Marker	1	2	3	4	5	6	7	8	9	10			Corr
Normalized dCt	**COL1A2**	1.14	−0.94	1.12	−1.89	−2.87	−0.89	0.96	1.15	1.10	1.10			0.77
	**MATN2**	1.37	−1.73	1.31	−1.81	−1.89	−1.74	1.04	1.15	1.16	1.16			0.68
	**SOX9**	0.08	0.16	−0.88	2.09	2.09	3.08	−0.88	−1.98	−1.89	−1.89			−0.66
	**TGFB1**	1.33	0.39	0.32	−0.69	−1.67	−0.70	0.21	0.27	0.28	0.28			0.91
	GeneScore	3.76	−2.44	3.61	−6.48	−8.52	−6.41	3.09	4.55	4.43	4.43			0.78

Supplementary Table 6 Source data for Fig 3e

OARSI Histological Assessment		7	10	13	3	6	8	6	5	6	6			
dCt	Symbol	1	2	3	4	5	6	7	8	9	10	ave	Weight	Corr
	CCN2(CTGF)	-2.9656	-2.99	-2.0329	-3.0106	-2.9905	-2.9707	-2	-3.94	-2.82	-2.82	-2.85403	50	0.47
	COL1A1	4.0431	1.06	2.9845	-0.0128	-0.9918	1.002	2.97	2.87	1.97	1.97	1.7865	28	0.27
	COL1A2	2.047	-0.03	2.0213	-0.9818	-1.9642	0.0124	1.87	2.06	2.01	2.01	0.90547	27	0.21
	COL2A1	-11.0047	-10.86	-9.0389	-10.0075	-9.9986	-10.9984	-9.08	-6.98	-8.02	-8.02	-9.40081	25	-0.25
	COL6A1	-2.9728	-3.88	-4.0028	-0.994	-2.9988	-0.9797	-3.13	-4.04	-4.06	-4.06	-3.11181	41	-0.34
	COMP	1.0275	0.11	1.0451	-2.0063	-2.9736	-2.0173	0.98	-1.08	-0.87	-0.87	-0.66546	52	0.52
	ACAN	-7.0121	-5.91	-6.0345	-5.0147	-6.9973	-5.0141	-6.14	-7.09	-6.95	-6.95	-6.31127	43	0.11
	ECM2	-8.9025	-11.01	-9.0091	-9.9383	-11.9366	-9.9988	-8.11	-9.06	-8.83	-8.83	-9.56253	11	-0.05
	FN1	3.0907	1.13	3.0251	0.1634	-0.9103	1.0321	4.21	2	2.26	2.26	1.8261	28	0.27
	SNX19	-8.99	-8.91	-7.965	-7.9974	-8.9979	-7.9651	-7.96	-8	-7.95	-7.95	-8.26854	35	-0.14
	LUM	2.0223	-0.99	1.9891	-1.9138	-2.8662	-1.9733	0.9	-0.02	0.24	0.24	-0.23719	44	0.39
	MATN2	-4.7702	-7.87	-4.8325	-7.9524	-8.0255	-7.877	-5.1	-4.99	-4.98	-4.98	-6.13776	30	0.11
	MGP	-8.1105	-11.13	-6.9535	-9.8372	-11.7893	-9.8377	-7.64	-6.92	-6.91	-6.91	-8.60382	22	0.03
	MMP3	-8.8942	-7.78	-6.9397	-8.9081	-8.9156	-7.8888	-8.14	-4.96	-4.76	-4.76	-7.19464	28	-0.02
	MMP13	-2.8611	-2.89	-1.9182	-3.9949	-4.9589	-3.9403	-2.12	-1.03	0.05	0.05	-2.36134	23	0.02
	SERPINF1	-8.8206	-7.82	-7.9639	-6.9758	-7.9654	-5.9915	-6	-6	-5.88	-5.88	-6.92972	41	-0.41
	SOX6	-7.9499	-8.81	-8.0079	-7.9978	-8.9731	-7.9962	-7.94	-8.9	-9	-9	-8.45749	29	0.17
	PTGS2	-7.9786	-6.89	-6.0181	-5.9539	-6.9557	-5.0045	-7.06	-9	-8.03	-8.03	-7.09208	49	0.33
	ACTB	-1.9955	-0.84	-1.0256	-0.0322	-1.9757	0.0006	-1.07	-2.12	-2.07	-2.07	-1.31984	44	0.14
	SOX9	-9.0044	-8.92	-9.9599	-6.9985	-6.9955	-6.0045	-9.96	-11.06	-10.97	-10.97	-9.08428	21	-0.08
	TGFB1	-1.9908	-2.93	-3.001	-4.0104	-4.9872	-4.0138	-3.11	-3.05	-3.04	-3.04	-3.31732	16	0.27
	TIMP1	-0.0072	-0.87	0.9753	-1.0185	-2.9772	-0.9994	1.08	0	-0.02	-0.02	-0.3857	39	0.28
	VEGFA	-6.0036	-5.88	-5.0144	-5.0125	-5.9469	-5.0116	-5.06	-6.07	-5.04	-5.04	-5.4079	27	0.04
	MIA	-5.0067	-4.92	-4.0445	-6.0268	-7.0055	-5.9739	-2.12	-5.05	-5.05	-5.05	-5.02474	41	0.26
	ITGA10	-7.9249	-6.91	-7.0019	-5.9954	-6.9785	-5.987	-6.14	-6.01	-6.93	-6.93	-6.68077	45	-0.36
	COL27A1	-6.8312	-6.93	-6.8749	-6.9523	-7.9277	-6.9453	-7.07	-8.02	-7.04	-7.04	-7.16314	41	0.36
	CD44	-1.9827	-2.9	-1.9881	-1.9585	-2.9398	-2.9542	-1.06	-1.97	-1.05	-1.05	-1.98533	26	-0.30
	GDF5	-10.0337	-9.72177	-9.29114	-8.89407	-9.52517	-8.94083	-8.24564	-8.95951	-9.32106	-9.32106	-9.22539	37	-0.32
	CHI3L1	-3.07629	-1.04436	-2.15193	-0.68843	-1.56021	-0.80631	-0.84734	-0.20991	-0.39275	-0.39275	-1.11703	42	-0.46
	SLC14A1	-6.18985	-5.73932	-6.18322	-6.00298	-6.05102	-6.10998	-5.95646	-6.42848	-6.79221	-6.79221	-6.22457	36	0.23
	ACKR4	-7.09358	-5.92536	-6.5111	-4.55019	-4.79475	-4.57069	-4.833	-4.0784	-4.40055	-4.40055	-5.11582	76	-0.65
	ESM1	-5.78454	-5.6019	-5.00078	-6.31876	-7.44041	-5.99155	-5.18054	-7.3377	-8.14868	-8.14868	-6.49535	67	0.54
	GREM1	-1.62234	-1.41603	-1.38249	-1.07265	-0.88165	-1.2348	-1.22787	-1.19476	-1.37212	-1.37212	-1.27768	50	-0.44
	LAMB3	-12.4681	-12.1783	-12.6957	-12.2737	-12.5432	-11.8793	-11.3977	-12.7199	-13.768	-13.768	-12.5692	15	0.08
	ECRG4(C2orf40)	-6.43748	-6.68077	-6.95285	-6.95071	-6.70694	-6.60886	-6.71116	-7.33705	-7.87219	-7.87219	-7.01302	24	0.25
	G0S2	-9.87822	-8.80164	-9.41247	-8.23331	-7.91401	-8.03359	-7.91659	-8.43953	-9.4839	-9.4839	-8.75971	29	-0.33
Marker		Patient 1	Patient 2	Patient 3	Patient 4	Patient 5	Patient 6	Patient 7	Patient 8	Patient 9	Patient 10			Corr
Normalized dC	**CCN2(CTGF)**	-0.11	-0.14	0.82	-0.16	-0.14	-0.12	0.85	-1.09	0.03	0.03			0.47
	**COMP**	1.69	0.78	1.71	-1.34	-2.31	-1.35	1.65	-0.41	-0.20	-0.20			0.52
	**ACKR4**	-1.98	-0.81	-1.40	0.57	0.32	0.55	0.28	1.04	0.72	0.72			-0.65
	**ESM1**	0.71	0.89	1.49	0.18	-0.95	0.50	1.31	-0.84	-1.65	-1.65			0.54
	**GREM1**	-0.34	-0.14	-0.10	0.21	0.40	0.04	0.05	0.08	-0.09	-0.09			-0.44
	GeneScore	4.61	2.48	5.53	-2.09	-4.11	-1.55	3.48	-3.46	-2.44	-2.44			0.66

Supplementary Table 7 Source data for Fig 3f

ICRS Ⅱ (Overall Assessment)		79	64	68	85	86	72	85	96	96	77			
dCt	Symbol	ENAD01	ENAD02	ENAD03	ENAD04	ENAD05	ENAD06	ENAD07	ENAD08	ENAD09	ENAD10	ave	Weight	Corr
	CCN2(CTGF)	−2.9656	−2.99	−2.0329	−3.0106	−2.9905	−2.9707	−2	−3.94	−2.82	−2.82	−2.854	35	−0.39
	COL1A1	4.0431	1.06	2.9845	−0.0128	−0.9918	1.002	2.97	2.87	1.97	1.97	1.7865	18	0.01
	COL1A2	2.047	−0.03	2.0213	−0.9818	−1.9642	0.0124	1.87	2.06	2.01	2.01	0.90547	34	0.13
	COL2A1	−11.005	−10.86	−9.0389	−10.008	−9.9986	−10.998	−9.08	−6.98	−8.02	−8.02	−9.4008	69	0.62
	COL6A1	−2.9728	−3.88	−4.0028	−0.994	−2.9988	−0.9797	−3.13	−4.04	−4.06	−4.06	−3.1118	22	−0.09
	COMP	1.0275	0.11	1.0451	−2.0063	−2.9736	−2.0173	0.98	−1.08	−0.87	−0.87	−0.6655	21	−0.33
	ACAN	−7.0121	−5.91	−6.0345	−5.0147	−6.9973	−5.0141	−6.14	−7.09	−6.95	−6.95	−6.3113	53	−0.46
	ECM2	−8.9025	−11.01	−9.0091	−9.9383	−11.937	−9.9988	−8.11	−9.06	−8.83	−8.83	−9.5625	35	0.25
	FN1	3.0907	1.13	3.0251	0.1634	−0.9103	1.0321	4.21	2	2.26	2.26	1.8261	14	−0.02
	SNX19	−8.99	−8.91	−7.965	−7.9974	−8.9979	−7.9651	−7.96	−8	−7.95	−7.95	−8.2685	32	0.25
	LUM	2.0223	−0.99	1.9891	−1.9138	−2.8662	−1.9733	0.9	−0.02	0.24	0.24	−0.2372	17	−0.07
	MATN2	−4.7702	−7.87	−4.8325	−7.9524	−8.0255	−7.877	−5.1	−4.99	−4.98	−4.98	−6.1378	39	0.28
	MGP	−8.1105	−11.13	−6.9535	−9.8372	−11.789	−9.8377	−7.64	−6.92	−6.91	−6.91	−8.6038	42	0.32
	MMP3	−8.8942	−7.78	−6.9397	−8.9081	−8.9156	−7.8888	−8.14	−4.96	−4.76	−4.76	−7.1946	50	0.34
	MMP13	−2.8611	−2.89	−1.9182	−3.9949	−4.9589	−3.9403	−2.12	−1.03	0.05	0.05	−2.3613	40	0.28
	SERPINF1	−8.8206	−7.82	−7.9639	−6.9758	−7.9654	−5.9915	−6	−6	−5.88	−5.88	−6.9297	41	0.43
	SOX6	−7.9499	−8.81	−8.0079	−7.9978	−8.9731	−7.9962	−7.94	−8.9	−9	−9	−8.4575	35	−0.33
	PTGS2	−7.9786	−6.89	−6.0181	−5.9539	−6.9557	−5.0045	−7.06	−9	−8.03	−8.03	−7.0921	63	−0.58
	ACTB	−1.9955	−0.84	−1.0256	−0.0322	−1.9757	0.0006	−1.07	−2.12	−2.07	−2.07	−1.3198	54	−0.48
	SOX9	−9.0044	−8.92	−9.9599	−6.9985	−6.9955	−6.0045	−9.96	−11.06	−10.97	−10.97	−9.0843	36	−0.30
	TGFB1	−1.9908	−2.93	−3.001	−4.0104	−4.9872	−4.0138	−3.11	−3.05	−3.04	−3.04	−3.3173	13	−0.13
	TIMP1	−0.0072	−0.87	0.9753	−1.0185	−2.9772	−0.9994	1.08	0	−0.02	−0.02	−0.3857	17	−0.04
	VEGFA	−6.0036	−5.88	−5.0144	−5.0125	−5.9469	−5.0116	−5.06	−6.07	−5.04	−5.04	−5.4079	34	−0.11
	MIA	−5.0067	−4.92	−4.0445	−6.0268	−7.0055	−5.9739	−2.12	−5.05	−5.05	−5.05	−5.0247	19	−0.07
	ITGA10	−7.9249	−6.91	−7.0019	−5.9954	−6.9785	−5.987	−6.14	−6.01	−6.93	−6.93	−6.6808	29	0.24
	COL27A1	−6.8312	−6.93	−6.8749	−6.9523	−7.9277	−6.9453	−7.07	−8.02	−7.04	−7.04	−7.1631	72	−0.57
	CD44	−1.9827	−2.9	−1.9881	−1.9585	−2.9398	−2.9542	−1.06	−1.97	−1.05	−1.05	−1.9853	48	0.45
	GDF5	−10.034	−9.7218	−9.2911	−8.8941	−9.5252	−8.9408	−8.2456	−8.9595	−9.3211	−9.3211	−9.2254	24	0.31
	CHI3L1	−3.0763	−1.0444	−2.1519	−0.6884	−1.5602	−0.8063	−0.8473	−0.2099	−0.3928	−0.3928	−1.117	41	0.40
	SLC14A1	−6.1899	−5.7393	−6.1832	−6.003	−6.051	−6.11	−5.9565	−6.4285	−6.7922	−6.7922	−6.2246	56	−0.48
	ACKR4	−7.0936	−5.9254	−6.5111	−4.5502	−4.7947	−4.5707	−4.833	−4.0784	−4.4005	−4.4005	−5.1158	65	0.59
	ESM1	−5.7845	−5.6019	−5.0008	−6.3188	−7.4404	−5.9916	−5.1805	−7.3377	−8.1487	−8.1487	−6.4954	67	−0.60
	GREM1	−1.6223	−1.416	−1.3825	−1.0726	−0.8816	−1.2348	−1.2279	−1.1948	−1.3721	−1.3721	−1.2777	33	0.36
	LAMB3	−12.468	−12.178	−12.696	−12.274	−12.543	−11.879	−11.398	−12.72	−13.768	−13.768	−12.569	37	−0.29
	ECRG4(C2orf40)	−6.4375	−6.6808	−6.9528	−6.9507	−6.7069	−6.6089	−6.7112	−7.337	−7.8722	−7.8722	−7.013	47	−0.45
	G0S2	−9.8782	−8.8016	−9.4125	−8.2333	−7.914	−8.0336	−7.9166	−8.4395	−9.4839	−9.4839	−8.7597	24	0.16
Marker	Marker	1	2	3	4	5	6	7	8	9	10			Corr
Normalized dCt	**COL2A1**	−1.60	−1.46	0.36	−0.61	−0.60	−1.60	0.32	2.42	1.38	1.38			0.62
	**COL27A1**	0.33	0.23	0.29	0.21	−0.76	0.22	0.09	−0.86	0.12	0.12			−0.57
	**ACKR4**	−1.98	−0.81	−1.40	0.57	0.32	0.55	0.28	1.04	0.72	0.72			0.59
	**ESM1**	0.71	0.89	1.49	0.18	−0.95	0.50	1.31	−0.84	−1.65	−1.65			−0.60
	GeneScore	−4.62	−3.40	−2.82	−0.43	1.43	−1.77	−0.80	5.16	3.63	3.63			0.73

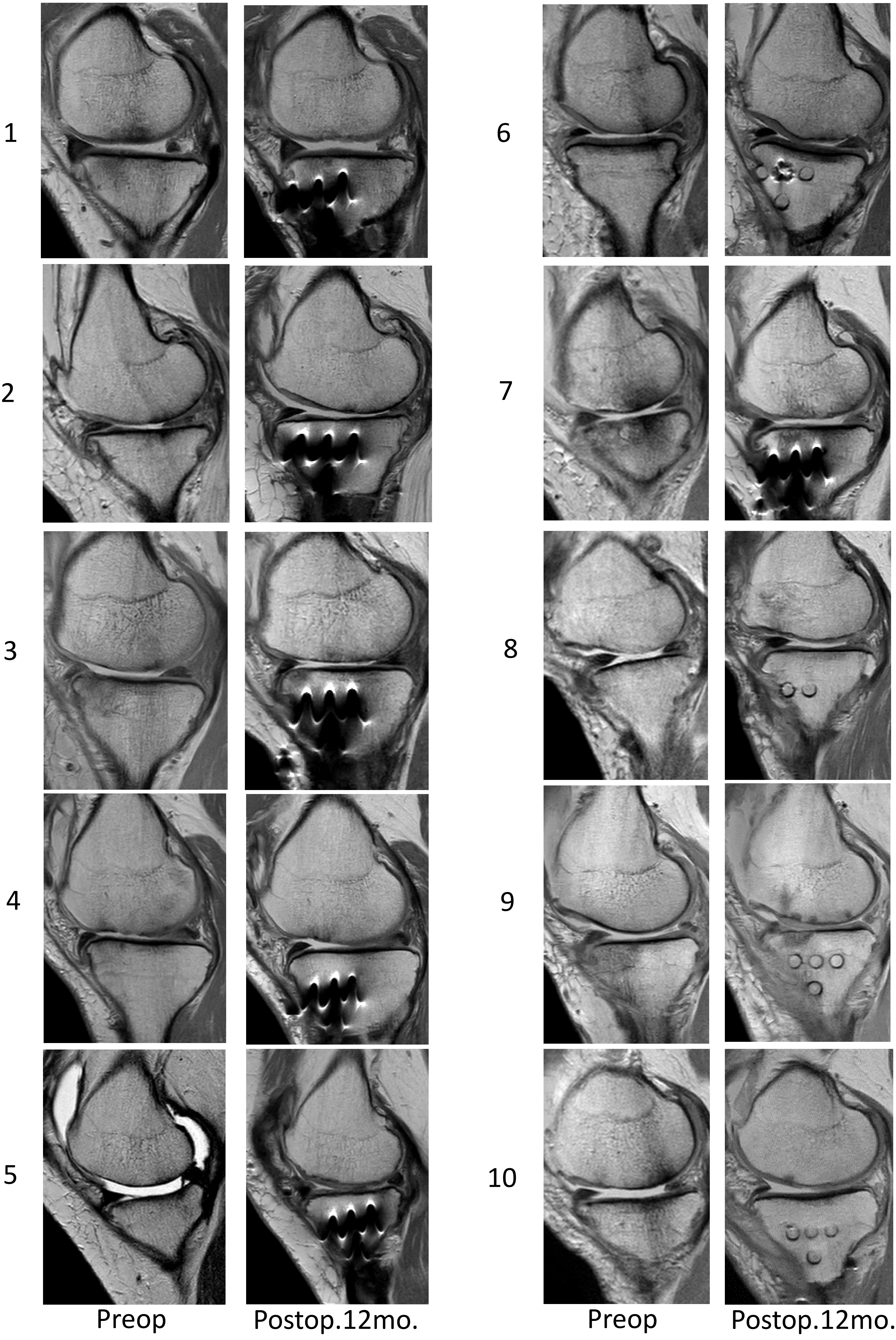

Supplementary Figure 1. Preoperative and postoperative MR images of all cases.

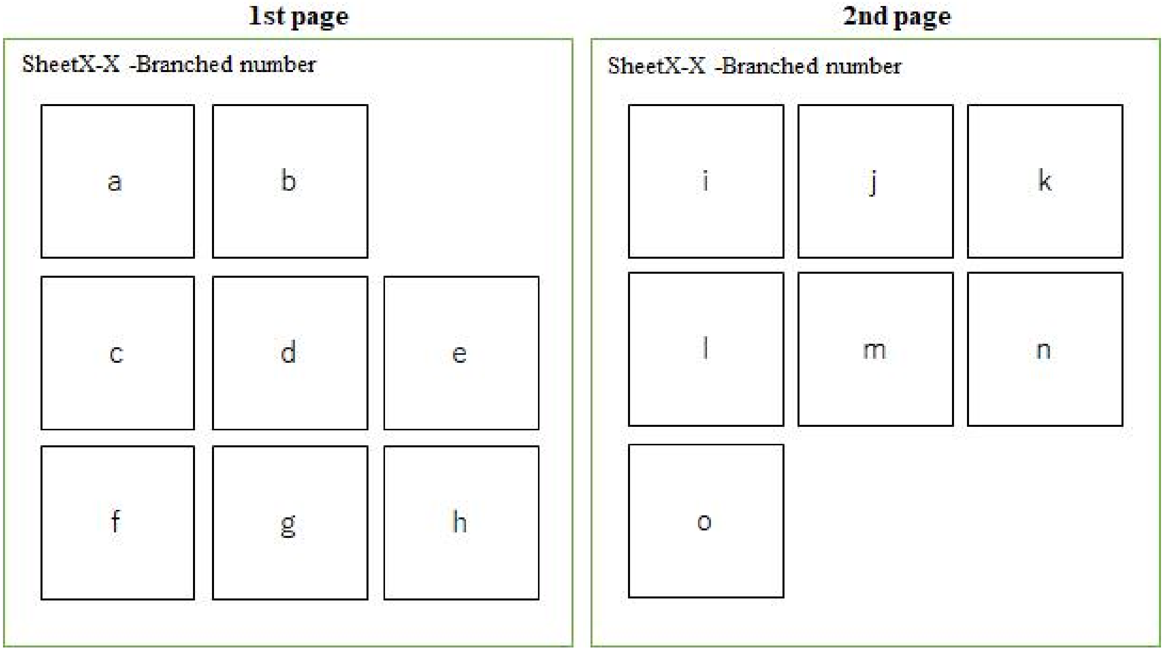

Flow cytometric analysis for PD sheets.

Data layouts and description on subsequent pages.
Labels on upper-left corner of each page is the identification of PD sheets. Branched number means 1st and 2 nd page of the same test sample.
Forward and side scatter gates were set on all dots to exclude debris (a). Then live cell gate were set on fraction of (a) to exclude dead cells as positive for propidium iodide (PI) (b). Live cells were analyzed on the surface marker expression.
(c-e, l) Dot plots for Isotype controls. Breaking lines for negative cells were set so 99% cells were included in lower-left (LL) gate.
(c) Setting for FITC-labeled antibodies used for analysis of CD31 (f), CD44 (g), CD45 (h).
(d) Setting for PE-labeled antibodies used for analysis of CD49a (i), CD105(o), CD146 (n).
(e) Setting for APC-labeled antibodies used for analysis of CD81 (j), CD90 (k).
(l) Setting for FITC-labeled goat anti mouse antibody used for analysis of GD2 (m).
Percentage of the positive cells in Figure 2f obtained as % Gated in lower-right (LR) gate.

Supplementary Figure 2. Raw data for Fig. 2f.

Sheet 1-1 -1

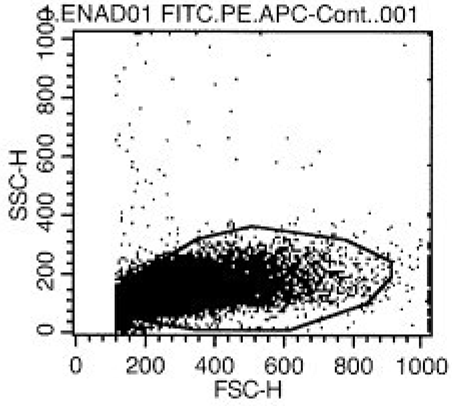

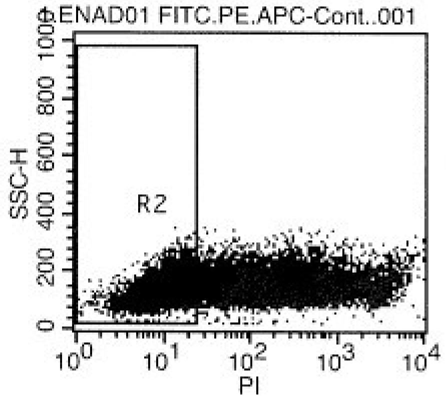

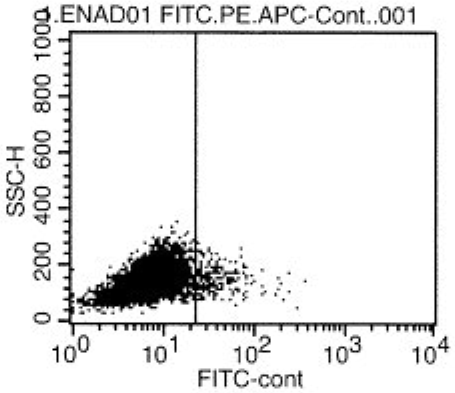

Quad	Events	% Gated
UL	0	0.00
UR	0	0.00
LL	6572	97.03
LR	201	2.97

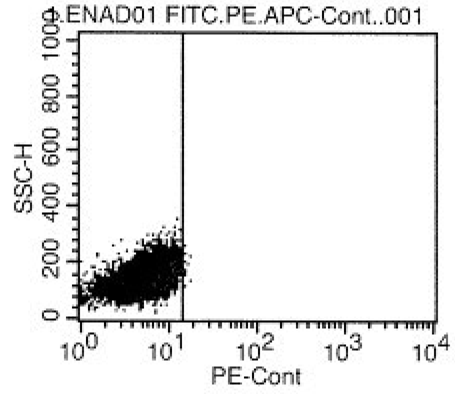

Quad	Events	% Gated
UL	0	0.00
UR	0	0.00
LL	6758	99.78
LR	15	0.22

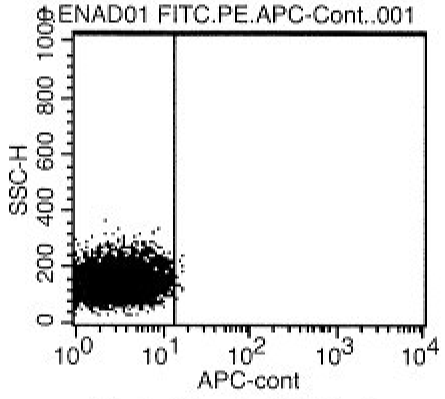

Quad	Events	% Gated
UL	0	0.00
UR	0	0.00
LL	6754	99.72
LR	19	0.28

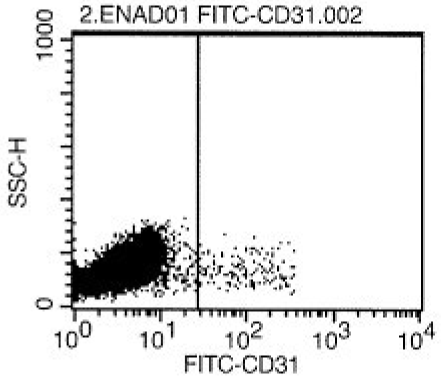

Quad	Events	% Gated
UL	0	0.00
UR	0	0.00
LL	9802	98.45
LR	154	1.55

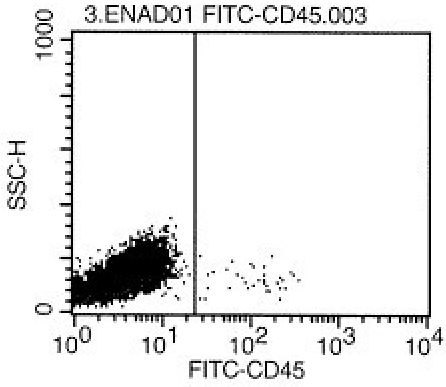

Quad	Events	% Gated
UL	0	0.00
UR	0	0.00
LL	11019	99.68
LR	35	0.32

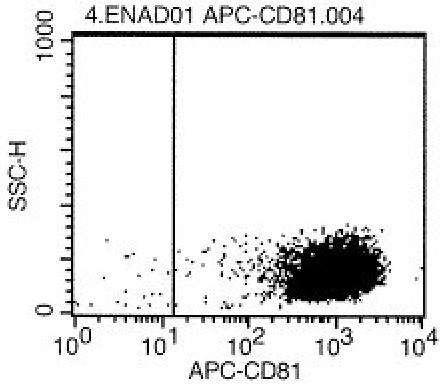

Quad	Events	% Gated
UL	0	0.00
UR	0	0.00
LL	21	0.22
LR	9649	99.78

Sheet1-1 -2

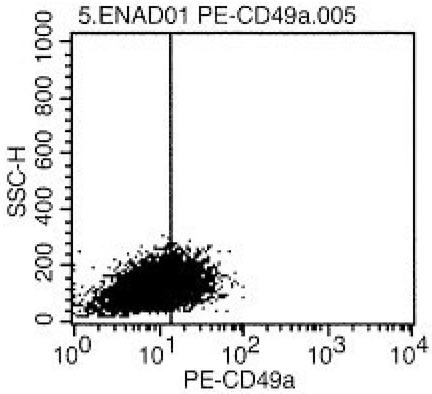

Quad	Events	% Gated
UL	0	0.00
UR	0	0.00
LL	7634	75.39
LR	2492	24.61

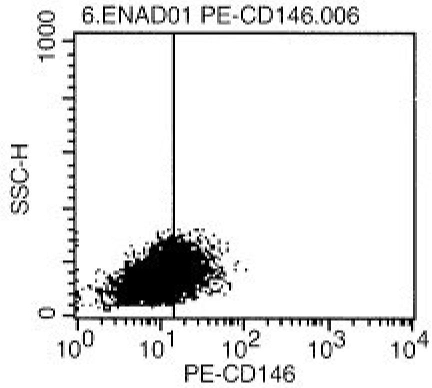

Quad	Events	% Gated
UL	0	0.00
UR	0	0.00
LL	6391	76.02
LR	2016	23.98

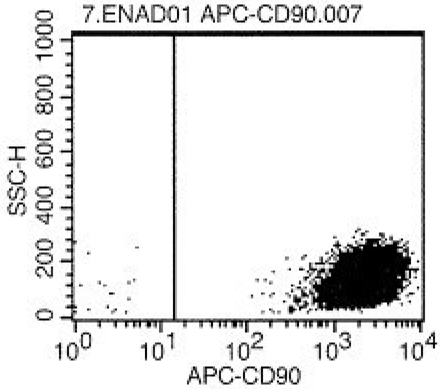

Quad	Events	% Gated
UL	0	0.00
UR	0	0.00
LL	18	0.18
LR	10128	99.82

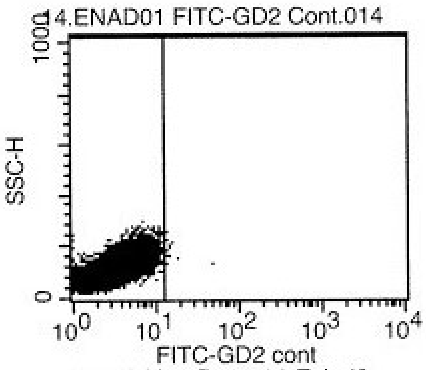

Acquisition Date: 14-Feb-1[illegible]

Quad	Events	% Gated
UL	0	0.00
UR	0	0.00
LL	9462	99.85
LR	14	0.15

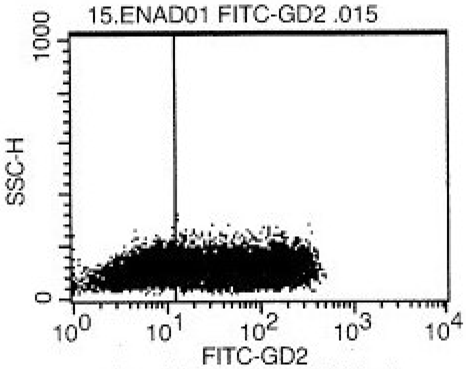

Acquisition Date: 14-Feb-1[illegible]

Quad	Events	% Gated
UL	0	0.00
UR	0	0.00
LL	2920	29.99
LR	6818	70.01

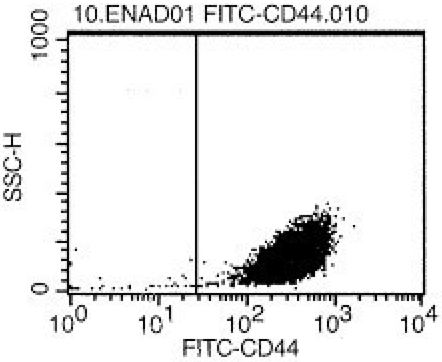

Quad	Events	% Gated
UL	0	0.00
UR	0	0.00
LL	34	0.29
LR	11791	99.71

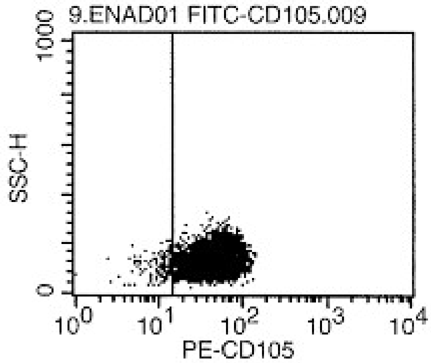

Quad	Events	% Gated
UL	0	0.00
UR	0	0.00
LL	144	2.70
LR	5180	97.30

Sheet1-2 -1

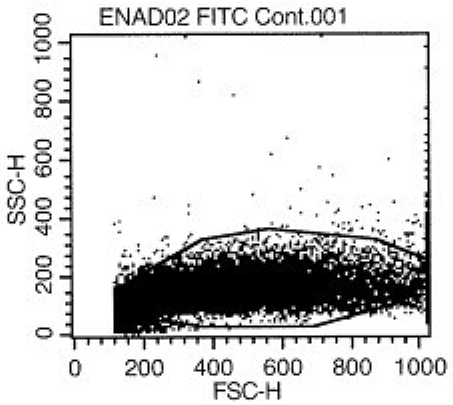

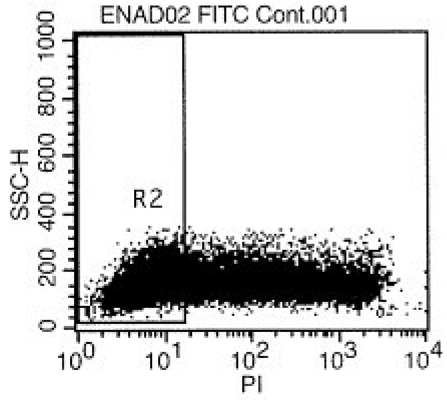

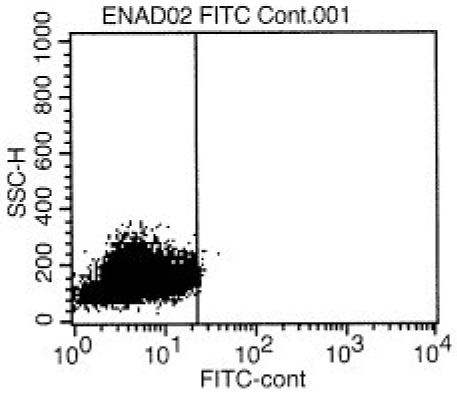

Quad	Events	% Gated
UL	0	0.00
UR	0	0.00
LL	7446	99.56
LR	33	0.44

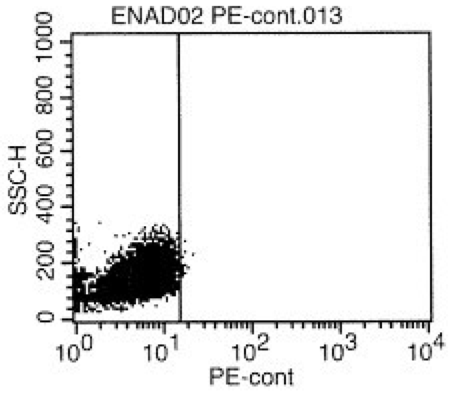

Quad	Events	% Gated
UL	0	0.00
UR	0	0.00
LL	6799	99.79
LR	14	0.21

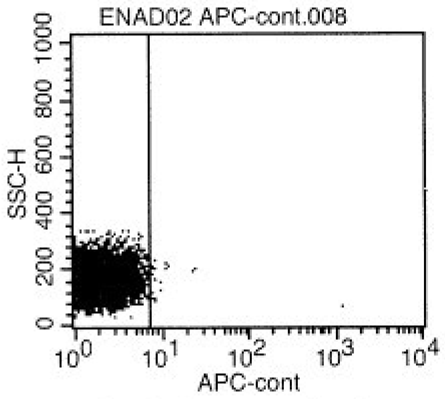

Quad	Events	% Gated
UL	0	0.00
UR	0	0.00
LL	7591	99.61
LR	30	0.39

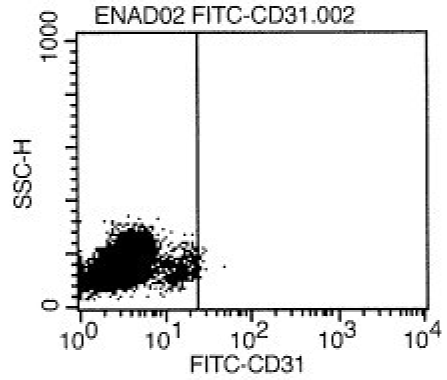

Quad	Events	% Gated
UL	0	0.00
UR	0	0.00
LL	6978	99.74
LR	18	0.26

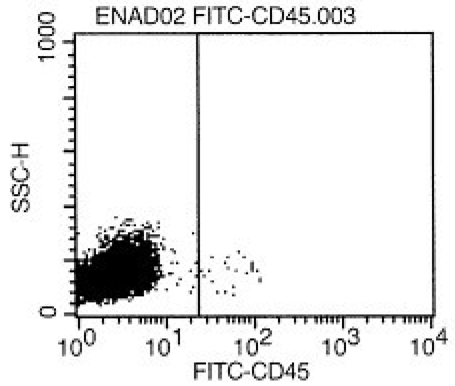

Quad	Events	% Gated
UL	0	0.00
UR	0	0.00
LL	7265	99.67
LR	24	0.33

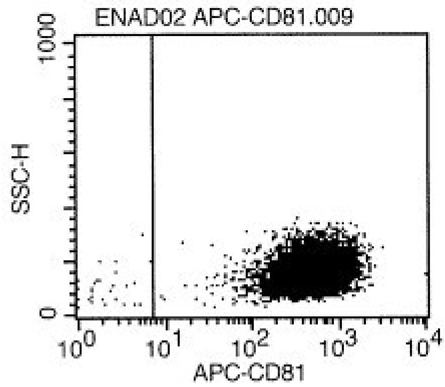

Quad	Events	% Gated
UL	0	0.00
UR	0	0.00
LL	27	0.36
LR	7454	99.64

Sheet1-2 -2

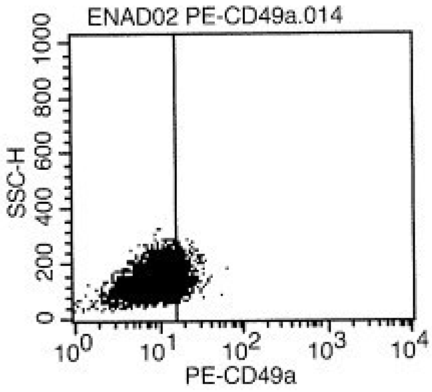

Quad	Events	% Gated
UL	0	0.00
UR	0	0.00
LL	4729	87.74
LR	661	12.26

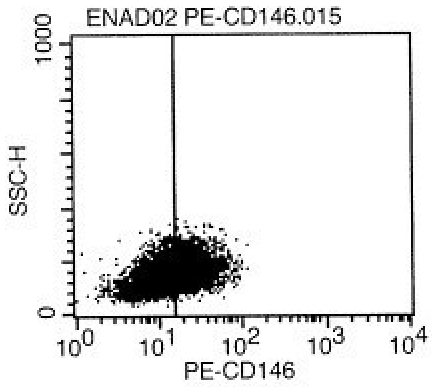

Quad	Events	% Gated
UL	0	0.00
UR	0	0.00
LL	4533	61.95
LR	2784	38.05

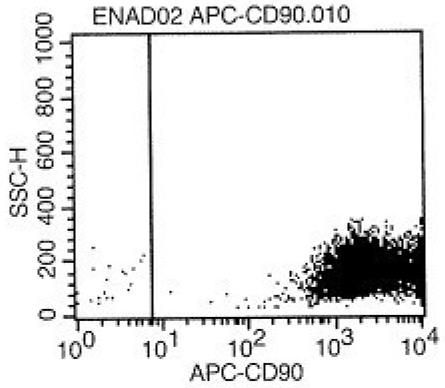

Quad	Events	% Gated
UL	0	0.00
UR	0	0.00
LL	20	0.36
LR	5511	99.64

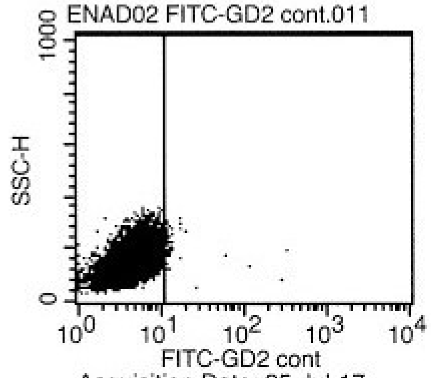

Acquisition Date: 25-Jul-17

Quad	Events	% Gated
UL	0	0.00
UR	0	0.00
LL	10364	99.28
LR	75	0.72

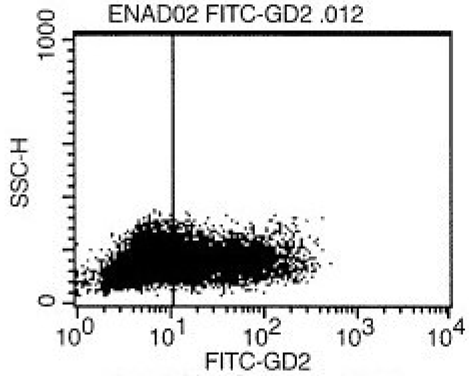

Acquisition Date: 25-Jul-17

Quad	Events	% Gated
UL	0	0.00
UR	0	0.00
LL	6736	67.41
LR	3256	32.59

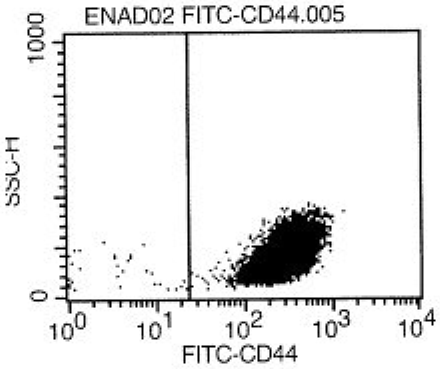

Quad	Events	% Gated
UL	0	0.00
UR	0	0.00
LL	31	0.32
LR	9770	99.68

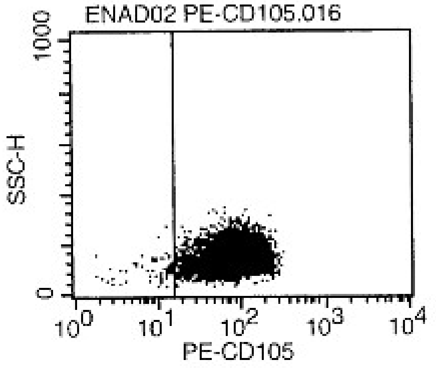

Quad	Events	% Gated
UL	0	0.00
UR	0	0.00
LL	76	1.32
LR	5696	98.68

Sheet1-3 -1

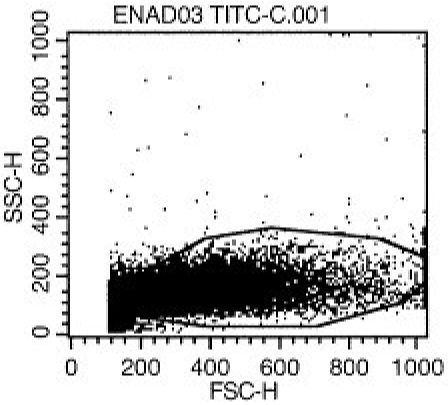

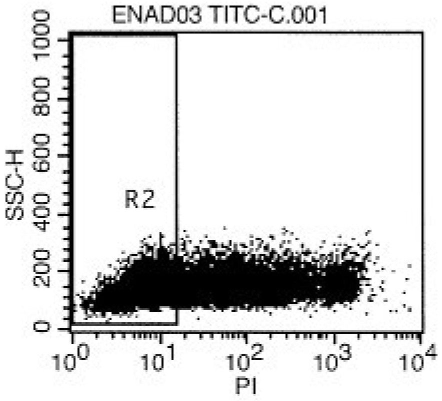

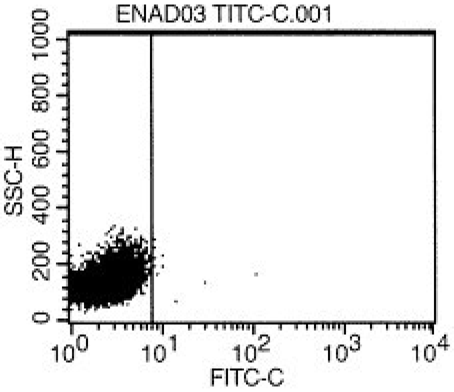

Quad	Events	% Gated
UL	0	0.00
UR	0	0.00
LL	6313	99.75
LR	16	0.25

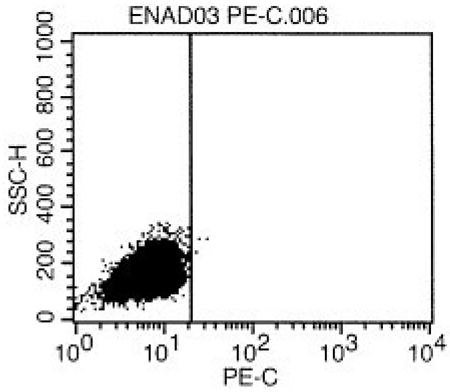

Quad	Events	% Gated
UL	0	0.00
UR	0	0.00
LL	8377	99.96
LR	3	0.04

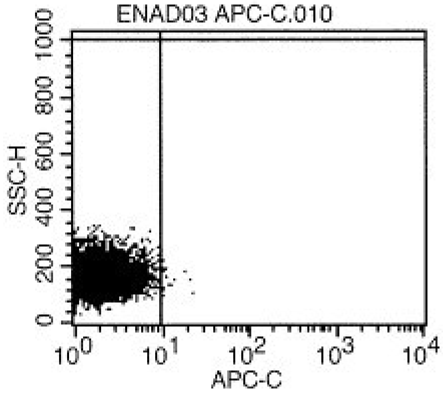

Quad	Events	% Gated
UL	0	0.00
UR	0	0.00
LL	7928	99.80
LR	16	0.20

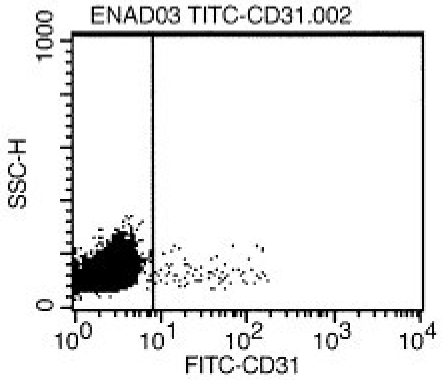

Quad	Events	% Gated
UL	0	0.00
UR	0	0.00
LL	6272	98.85
LR	73	1.15

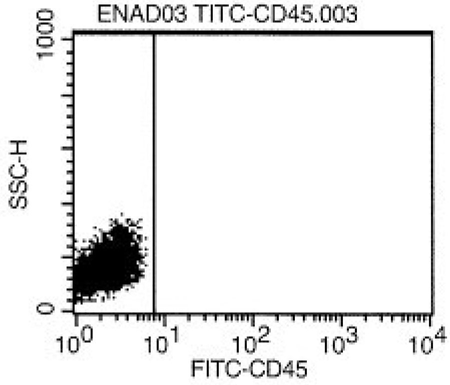

Quad	Events	% Gated
UL	0	0.00
UR	0	0.00
LL	7723	100.00
LR	0	0.00

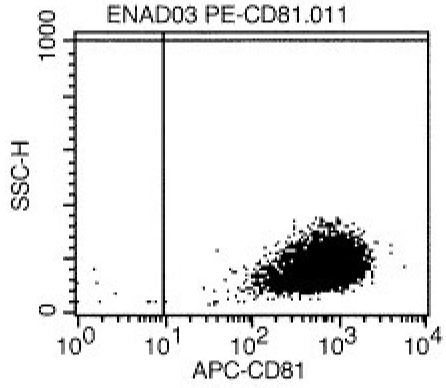

Quad	Events	% Gated
UL	0	0.00
UR	0	0.00
LL	9	0.12
LR	7816	99.88

Sheet1-3 -2

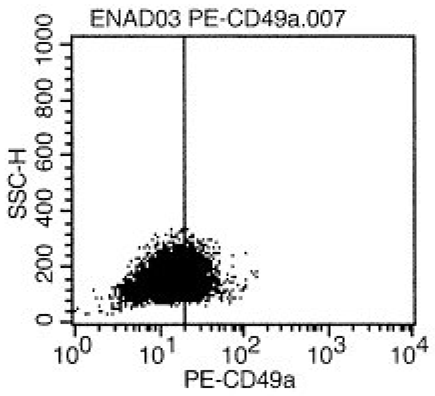

Quad	Events	% Gated
UL	0	0.00
UR	0	0.00
LL	5969	77.16
LR	1767	22.84

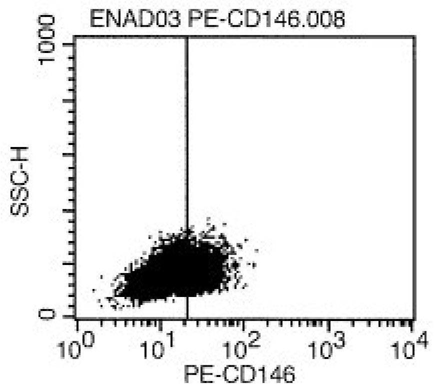

Quad	Events	% Gated
UL	0	0.00
UR	0	0.00
LL	5835	74.75
LR	1971	25.25

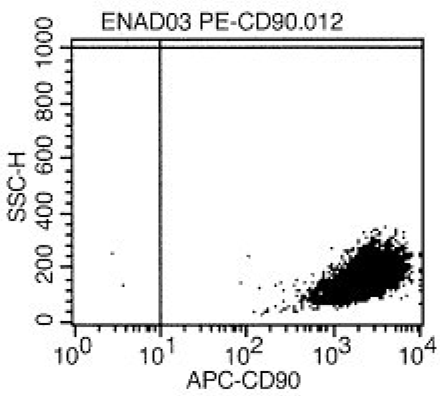

Quad	Events	% Gated
UL	0	0.00
UR	0	0.00
LL	2	0.03
LR	7537	99.97

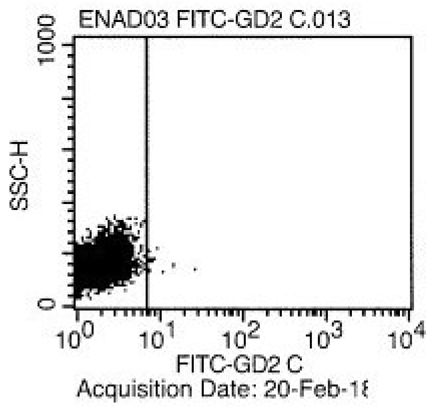

Quad	Events	% Gated
UL	0	0.00
UR	0	0.00
LL	9268	99.86
LR	13	0.14

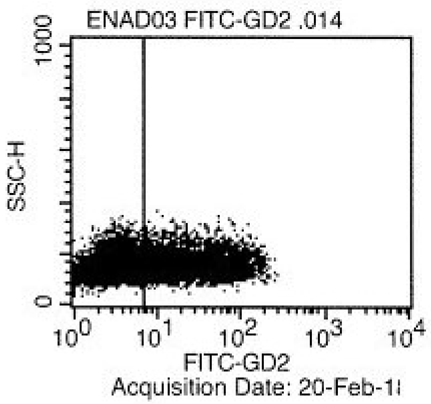

Quad	Events	% Gated
UL	0	0.00
UR	0	0.00
LL	4674	56.03
LR	3668	43.97

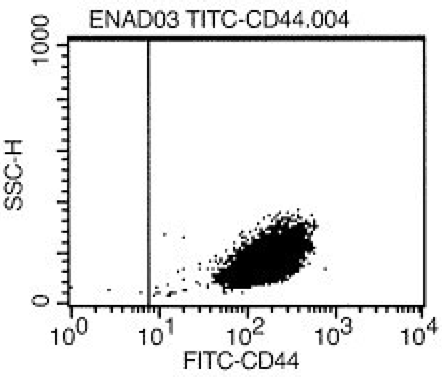

Quad	Events	% Gated
UL	0	0.00
UR	0	0.00
LL	4	0.03
LR	12744	99.97

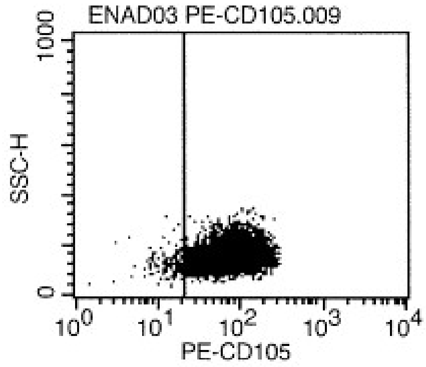

Quad	Events	% Gated
UL	0	0.00
UR	0	0.00
LL	170	2.56
LR	6472	97.44

Sheet2-1 -1

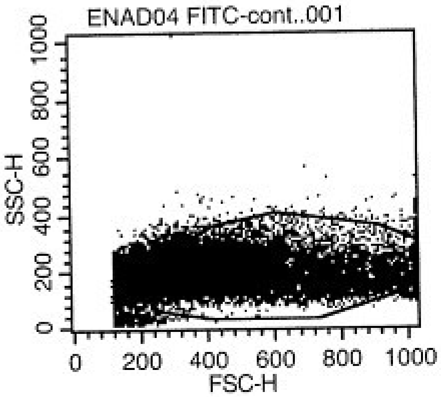

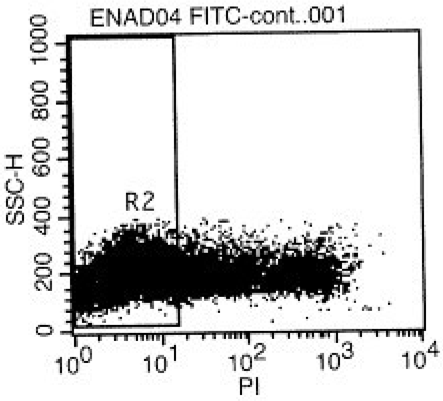

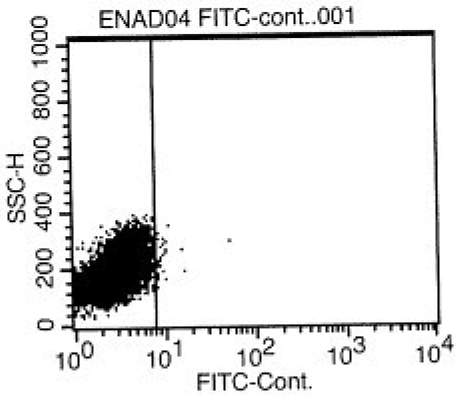

Quad	Events	% Gated
UL	0	0.00
UR	0	0.00
LL	10578	99.59
LR	44	0.41

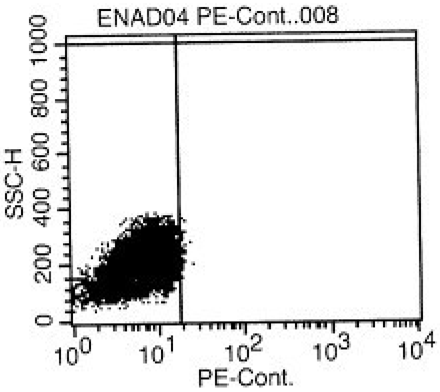

Quad	Events	% Gated
UL	0	0.00
UR	0	0.00
LL	9973	99.72
LR	28	0.28

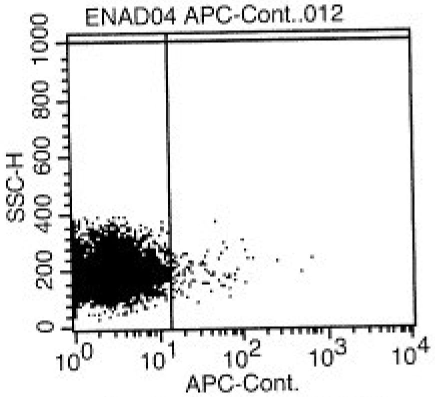

Quad	Events	% Gated
UL	0	0.00
UR	0	0.00
LL	10310	99.06
LR	98	0.94

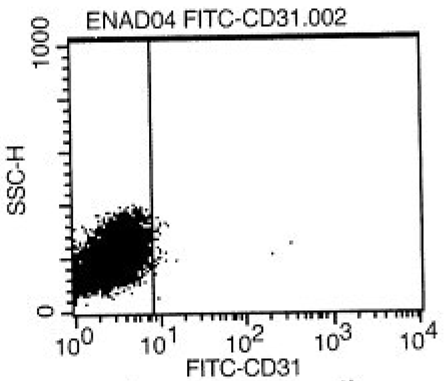

Quad	Events	% Gated
UL	0	0.00
UR	0	0.00
LL	10565	99.57
LR	46	0.43

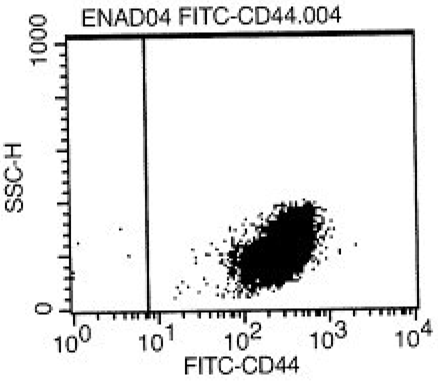

Quad	Events	% Gated
UL	0	0.00
UR	0	0.00
LL	5	0.04
LR	11462	99.96

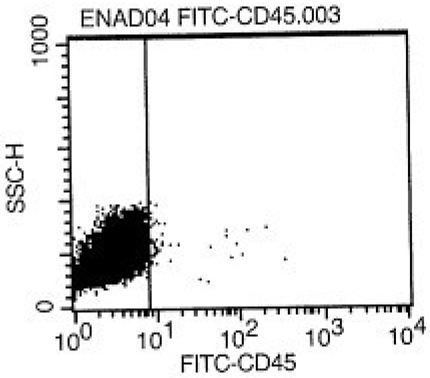

Quad	Events	% Gated
UL	0	0.00
UR	0	0.00
LL	10490	99.34
LR	70	0.66

Sheet2-1 -2

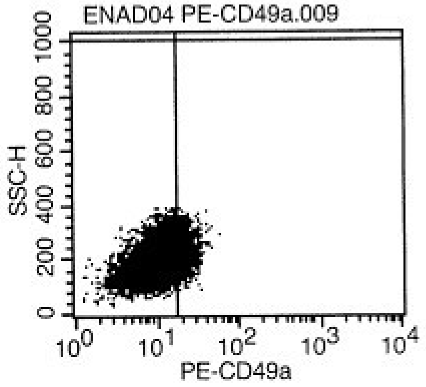

Quad	Events	% Gated
UL	0	0.00
UR	0	0.00
LL	8463	86.91
LR	1275	13.09

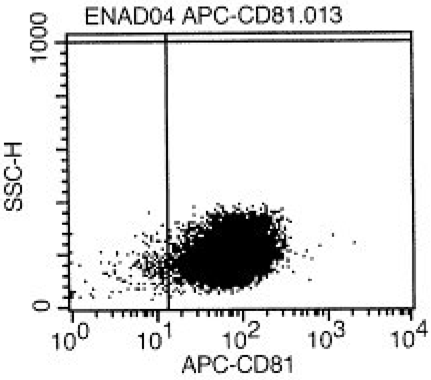

Quad	Events	% Gated
UL	0	0.00
UR	0	0.00
LL	198	1.78
LR	10951	98.22

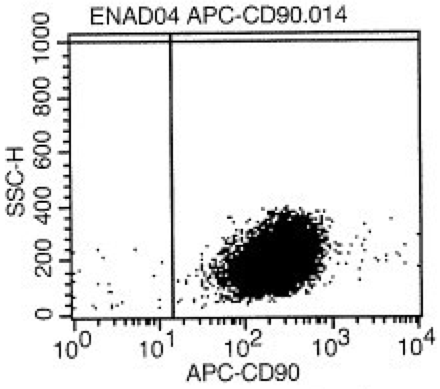

Quad	Events	% Gated
UL	0	0.00
UR	0	0.00
LL	23	0.24
LR	9694	99.76

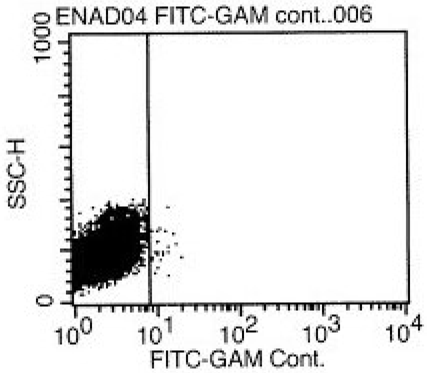

Quad	Events	% Gated
UL	0	0.00
UR	0	0.00
LL	10262	99.73
LR	28	0.27

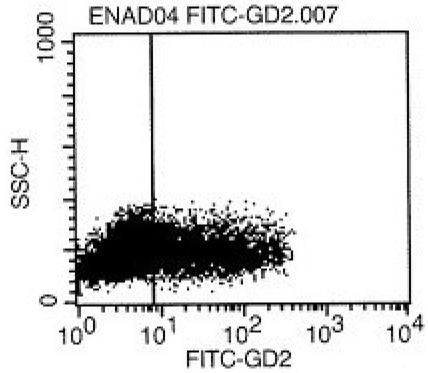

Quad	Events	% Gated
UL	0	0.00
UR	0	0.00
LL	6962	66.40
LR	3523	33.60

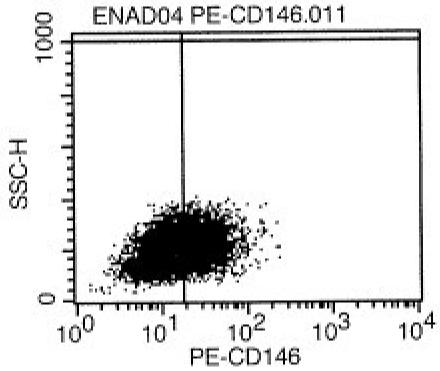

Quad	Events	% Gated
UL	0	0.00
UR	0	0.00
LL	6242	62.44
LR	3755	37.56

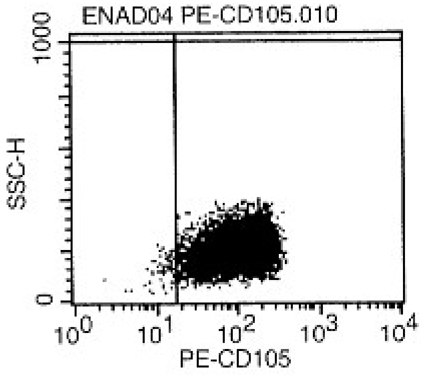

Quad	Events	% Gated
UL	0	0.00
UR	0	0.00
LL	82	0.92
LR	8805	99.08

Sheet2-2 -1

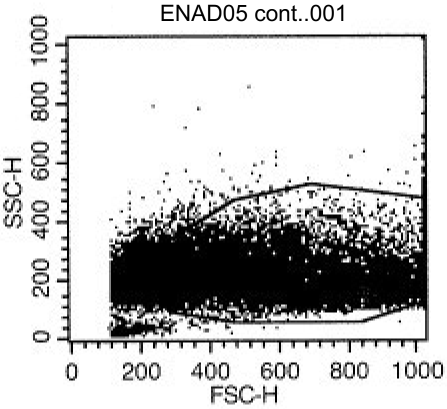

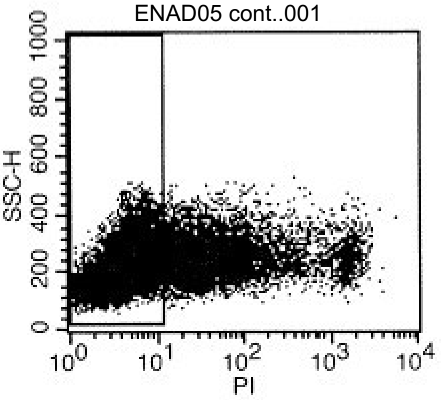

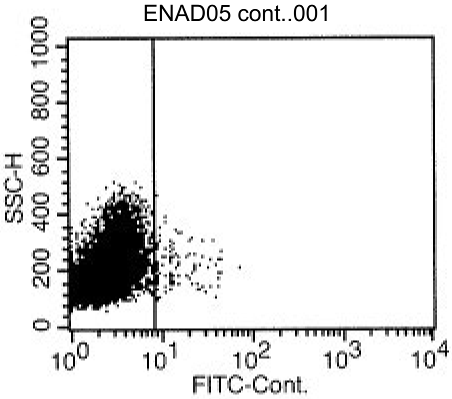

Quad	Events	% Gated
UL	0	0.00
UR	0	0.00
LL	11581	99.10
LR	105	0.90

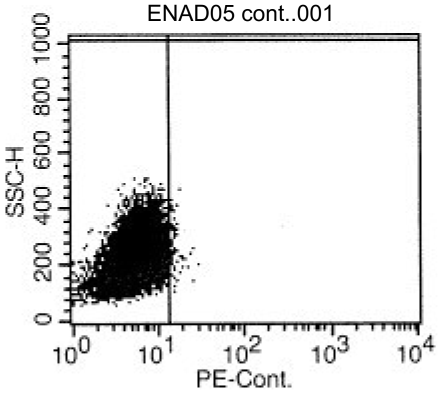

Quad	Events	% Gated
UL	0	0.00
UR	0	0.00
LL	11622	99.45
LR	64	0.55

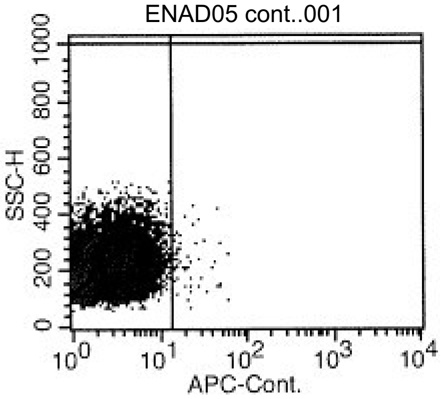

Quad	Events	% Gated
UL	0	0.00
UR	0	0.00
LL	11625	99.48
LR	61	0.52

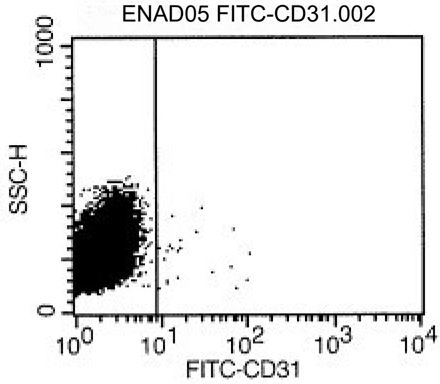

Quad	Events	% Gated
UL	0	0.00
UR	0	0.00
LL	11149	99.79
LR	23	0.21

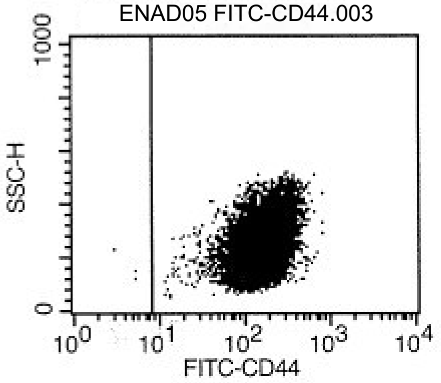

Quad	Events	% Gated
UL	0	0.00
UR	0	0.00
LL	3	0.02
LR	13588	99.98

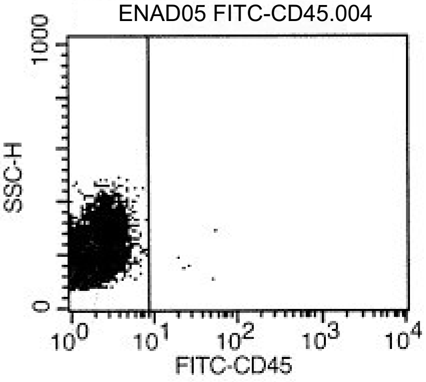

Quad	Events	% Gated
UL	0	0.00
UR	0	0.00
LL	11092	99.95
LR	6	0.05

Sheet2-2 -2

ENAD05 PE-CD49a 006

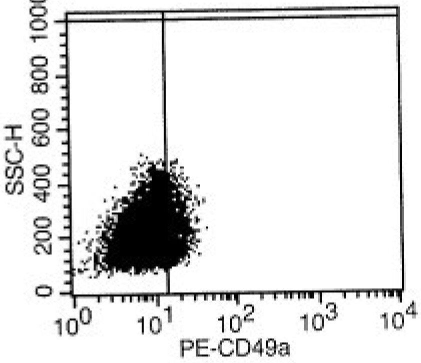

Quad	Events	% Gated
UL	0	0.00
UR	0	0.00
LL	8569	86.69
LR	1316	13.31

ENAD05 PE-CD81.009

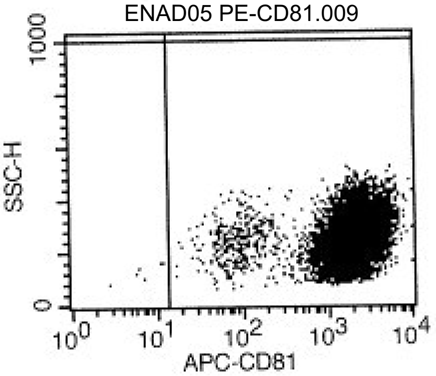

Quad	Events	% Gated
UL	0	0.00
UR	0	0.00
LL	6	0.06
LR	9652	99.94

ENAD05 PE-CD90.010

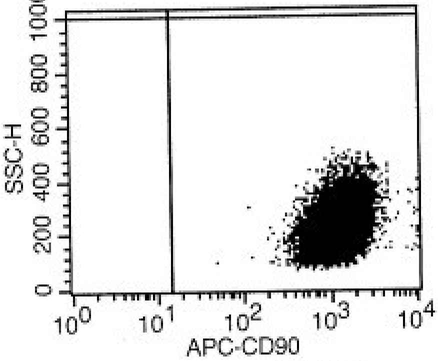

Quad	Events	% Gated
UL	0	0.00
UR	0	0.00
LL	0	0.00
LR	9967	100.00

ENAD05 FITC-GAM Cont..014

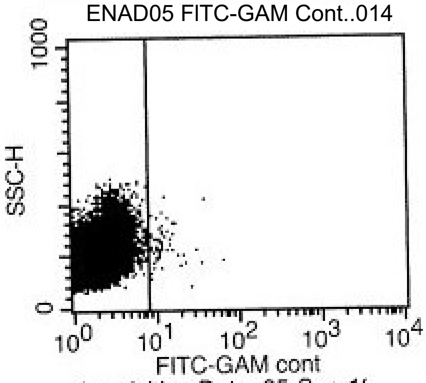

Acquisition Date: 25-Sep-1[illegible]

Quad	Events	% Gated
UL	0	0.00
UR	0	0.00
LL	12678	99.48
LR	66	0.52

ENAD05 FITC-GD2.015

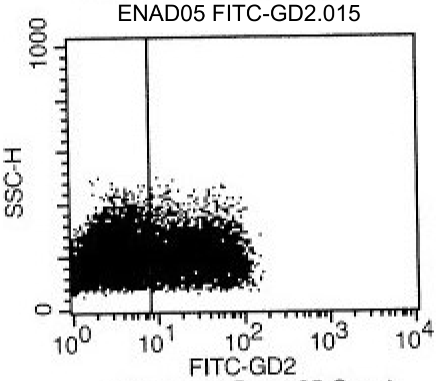

Acquisition Date: 25-Sep-1[illegible]

Quad	Events	% Gated
UL	0	0.00
UR	0	0.00
LL	6540	63.63
LR	3738	36.37

ENAD05 PE-CD146.008

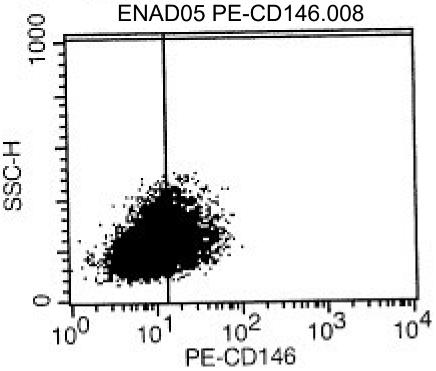

Quad	Events	% Gated
UL	0	0.00
UR	0	0.00
LL	7627	74.94
LR	2551	25.06

ENAD05 PE-GD105.007

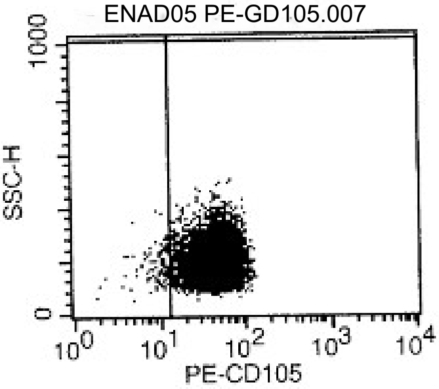

Quad	Events	% Gated
UL	0	0.00
UR	0	0.00
LL	143	2.37
LR	5898	97.63

Sheet2-3 -1

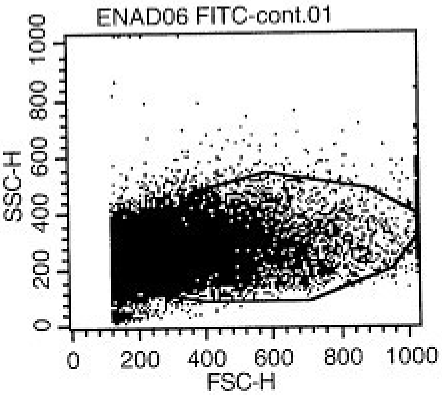

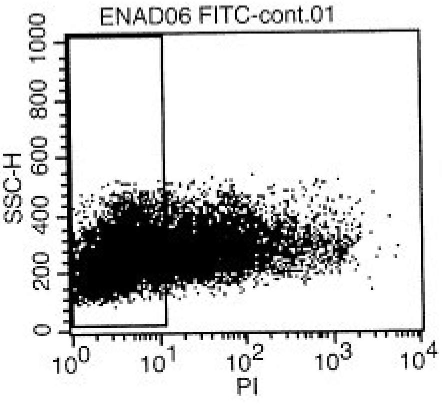

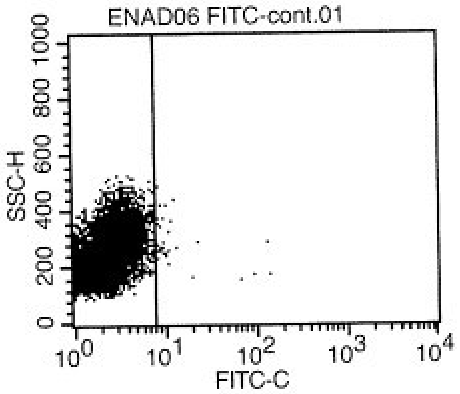

Quad	Events	% Gated
UL	0	0.00
UR	0	0.00
LL	7254	99.40
LR	44	0.60

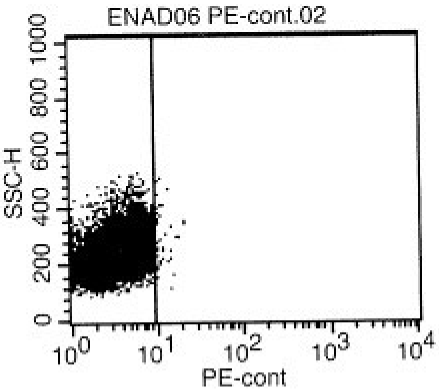

Quad	Events	% Gated
UL	0	0.00
UR	0	0.00
LL	5631	99.31
LR	39	0.69

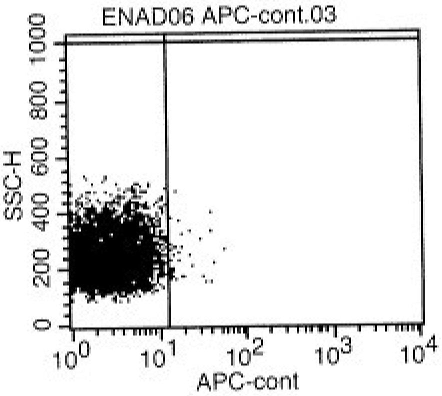

Quad	Events	% Gated
UL	0	0.00
UR	0	0.00
LL	5574	99.31
LR	39	0.69

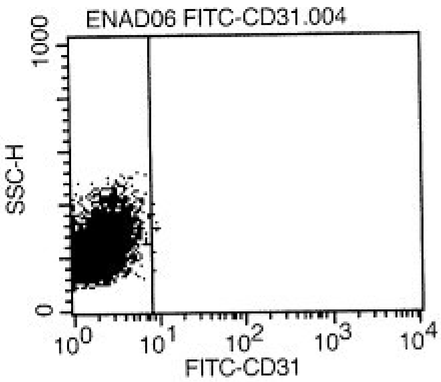

Quad	Events	% Gated
UL	0	0.00
UR	0	0.00
LL	5615	99.84
LR	9	0.16

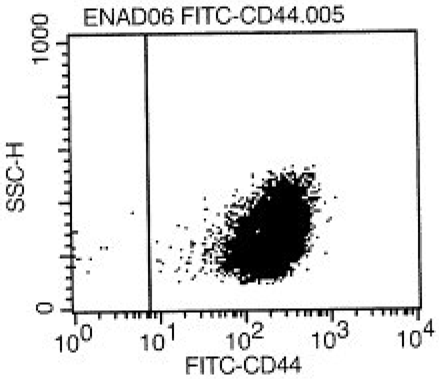

Quad	Events	% Gated
UL	0	0.00
UR	0	0.00
LL	12	0.12
LR	10202	99.88

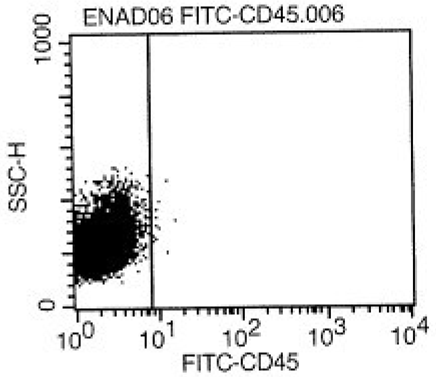

Quad	Events	% Gated
UL	0	0.00
UR	0	0.00
LL	5867	99.68
LR	19	0.32

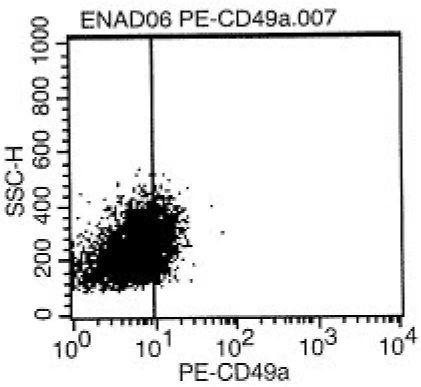

Quad	Events	% Gated
UL	0	0.00
UR	0	0.00
LL	4146	75.15
LR	1371	24.85

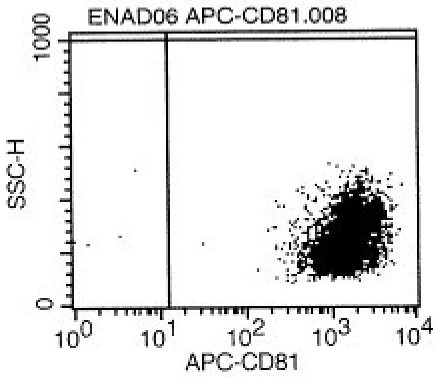

Quad	Events	% Gated
UL	0	0.00
UR	0	0.00
LL	4	0.10
LR	4203	99.90

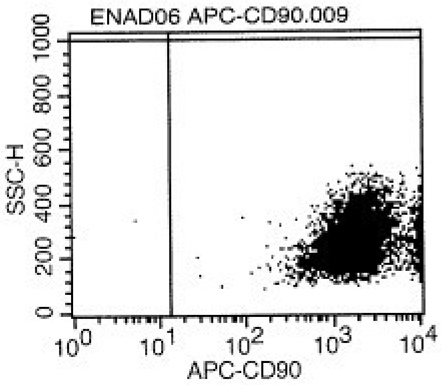

Quad	Events	% Gated
UL	0	0.00
UR	0	0.00
LL	2	0.04
LR	5452	99.96

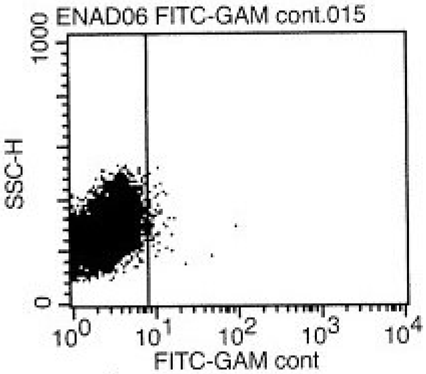

Quad	Events	% Gated
UL	0	0.00
UR	0	0.00
LL	7655	99.12
LR	68	0.88

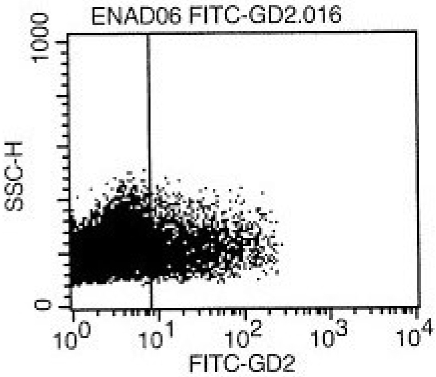

Quad	Events	% Gated
UL	0	0.00
UR	0	0.00
LL	7034	78.58
LR	1917	21.42

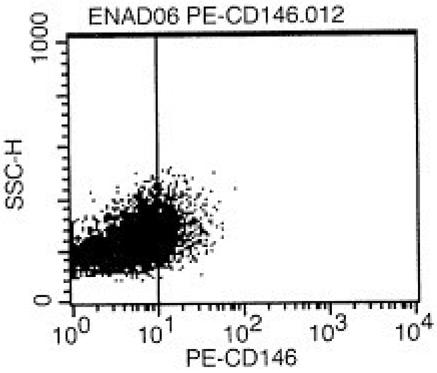

Quad	Events	% Gated
UL	0	0.00
UR	0	0.00
LL	4556	80.10
LR	1132	19.90

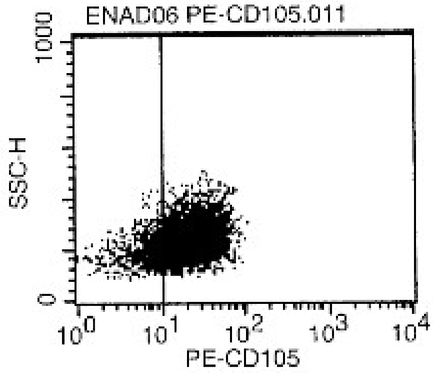

Quad	Events	% Gated
UL	0	0.00
UR	0	0.00
LL	886	20.83
LR	3367	79.17

Sheet2-4 -1

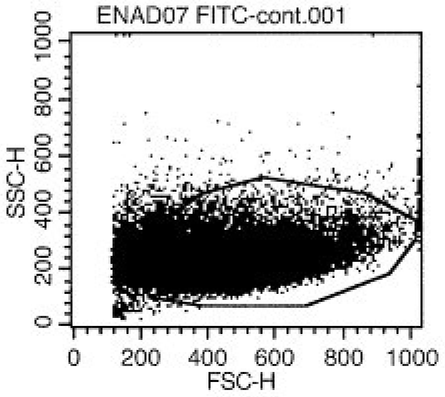

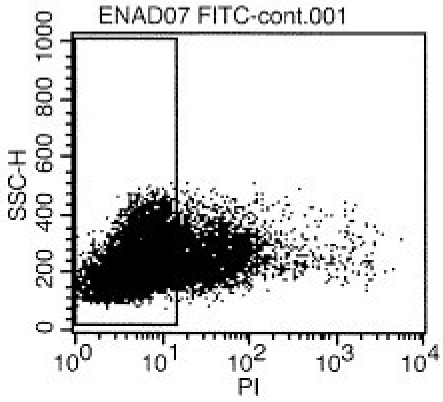

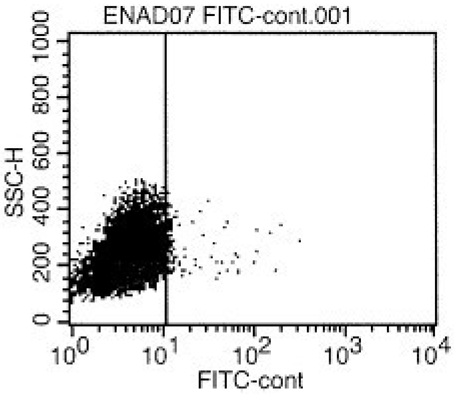

Quad	Events	% Gated
UL	0	0.00
UR	0	0.00
LL	15023	99.19
LR	122	0.81

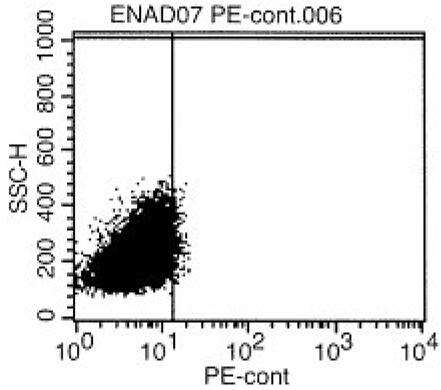

Quad	Events	% Gated
UL	0	0.00
UR	0	0.00
LL	14908	99.06
LR	142	0.94

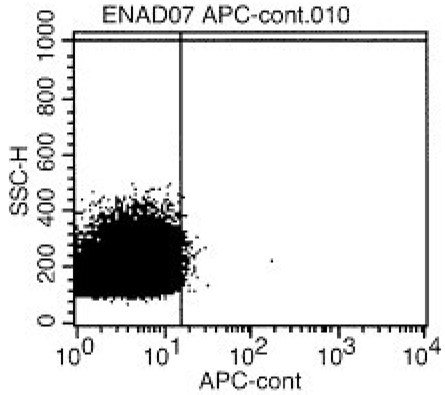

Quad	Events	% Gated
UL	0	0.00
UR	0	0.00
LL	15615	99.31
LR	109	0.69

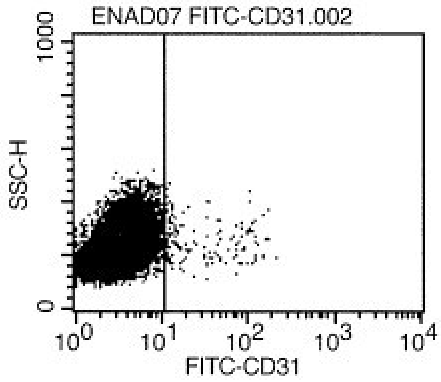

Quad	Events	% Gated
UL	0	0.00
UR	0	0.00
LL	14500	98.98
LR	149	1.02

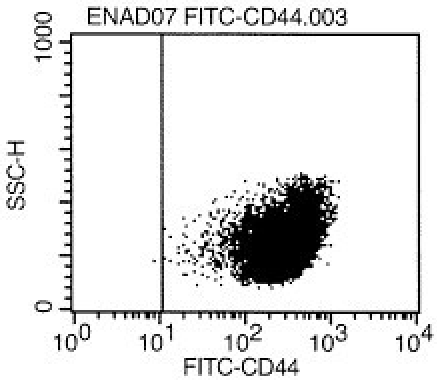

Quad	Events	% Gated
UL	0	0.00
UR	0	0.00
LL	2	0.01
LR	16426	99.99

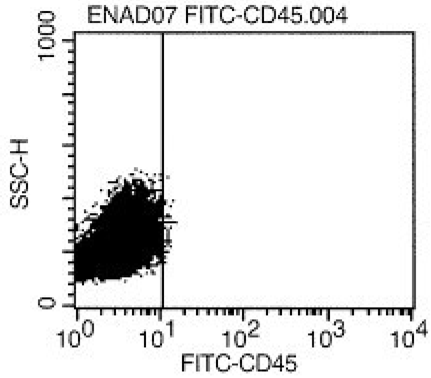

Quad	Events	% Gated
UL	0	0.00
UR	0	0.00
LL	16304	99.75
LR	41	0.25

Sheet2-4 -2

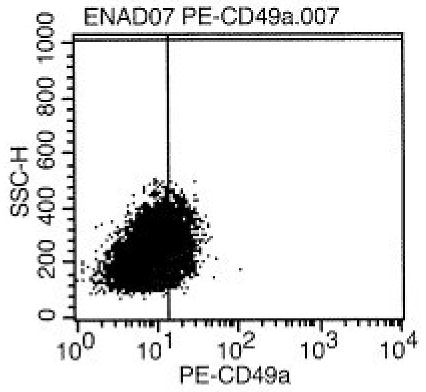

Quad	Events	% Gated
UL	0	0.00
UR	0	0.00
LL	12682	80.72
LR	3029	19.28

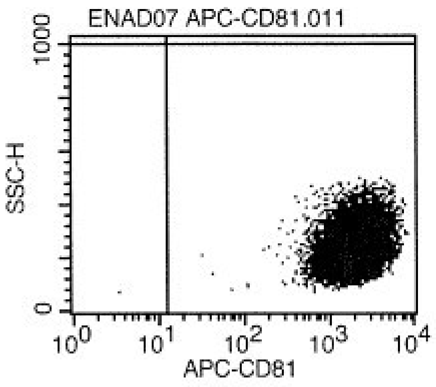

Quad	Events	% Gated
UL	0	0.00
UR	0	0.00
LL	1	0.01
LR	16487	99.99

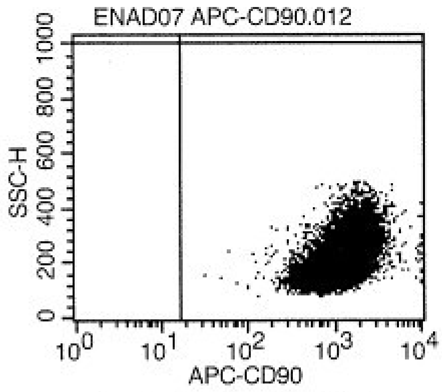

Quad	Events	% Gated
UL	0	0.00
UR	0	0.00
LL	0	0.00
LR	14336	100.00

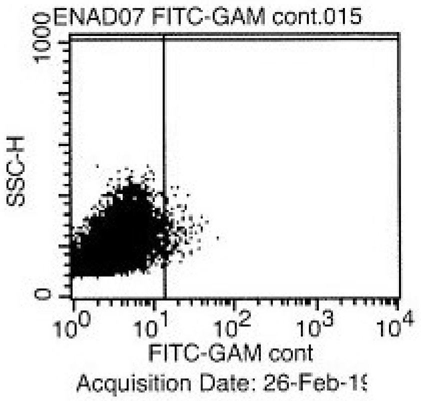

Quad	Events	% Gated
UL	0	0.00
UR	0	0.00
LL	16604	99.17
LR	139	0.83

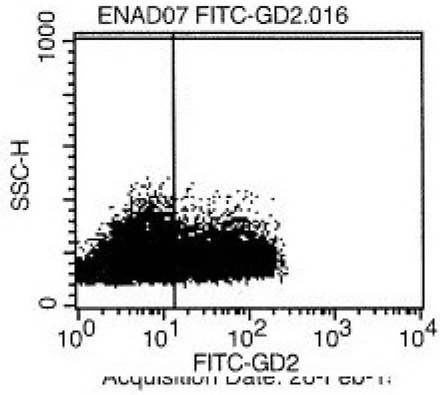

Quad	Events	% Gated
UL	0	0.00
UR	0	0.00
LL	11115	72.96
LR	4119	27.04

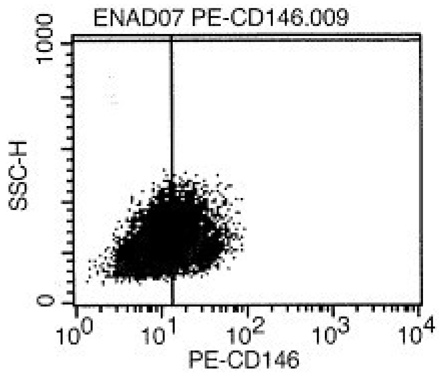

Quad	Events	% Gated
UL	0	0.00
UR	0	0.00
LL	10451	68.62
LR	4779	31.38

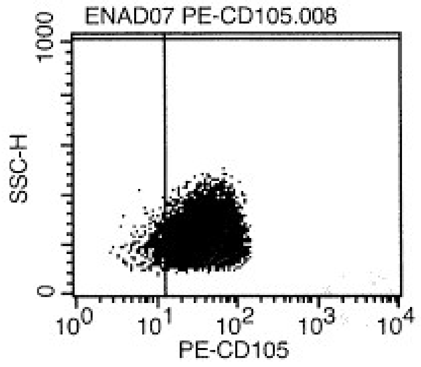

Quad	Events	% Gated
UL	0	0.00
UR	0	0.00
LL	490	3.94
LR	11943	96.06

Sheet3-1 -1

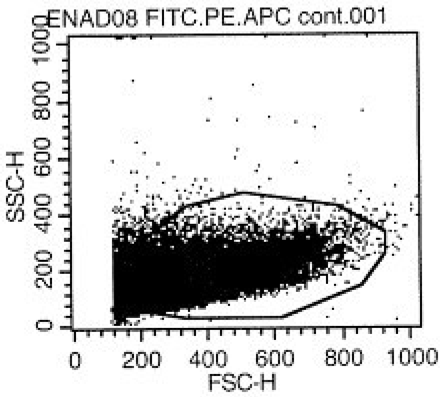

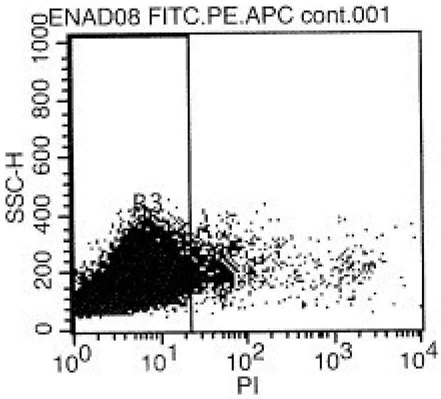

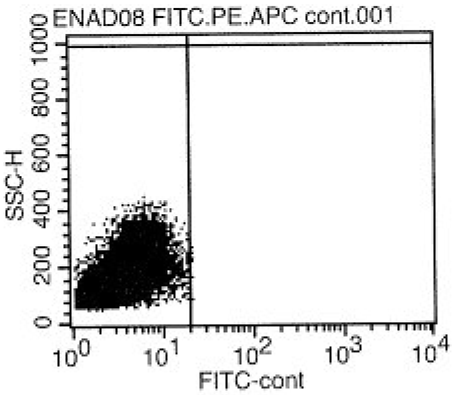

Quad	Events	% Gated
UL	0	0.00
UR	0	0.00
LL	15786	99.85
LR	23	0.15

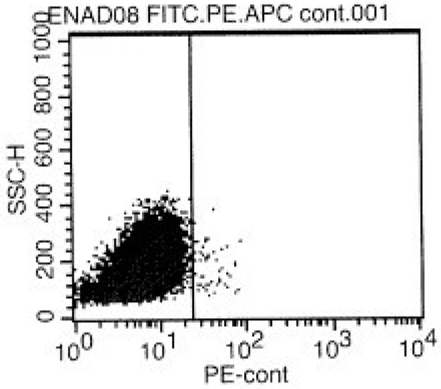

Quad	Events	% Gated
UL	0	0.00
UR	0	0.00
LL	16074	99.67
LR	53	0.33

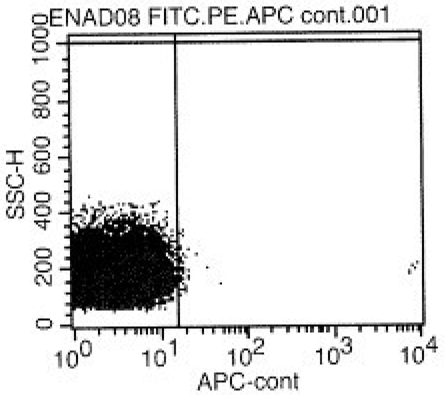

Quad	Events	% Gated
UL	0	0.00
UR	0	0.00
LL	15759	99.68
LR	50	0.32

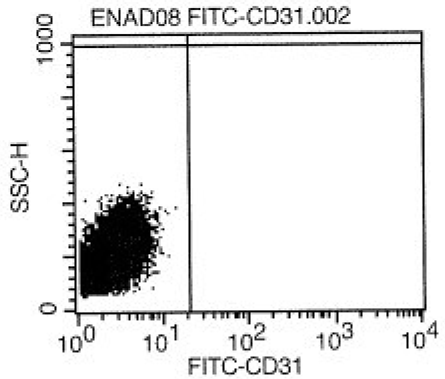

Quad	Events	% Gated
UL	0	0.00
UR	0	0.00
LL	16096	100.00
LR	0	0.00

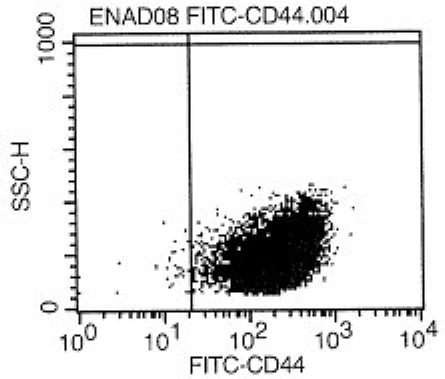

Quad	Events	% Gated
UL	0	0.00
UR	0	0.00
LL	35	0.19
LR	18098	99.81

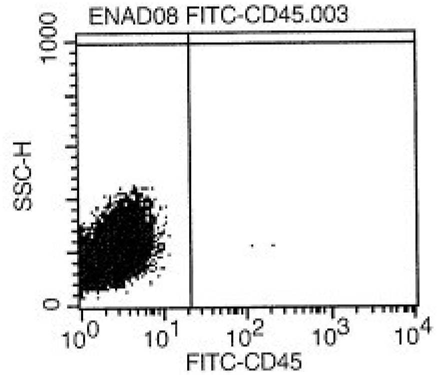

Quad	Events	% Gated
UL	0	0.00
UR	0	0.00
LL	17441	99.99
LR	2	0.01

Sheet3-1 -2

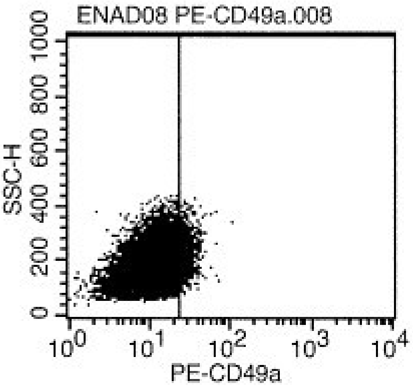

Quad	Events	% Gated
UL	0	0.00
UR	0	0.00
LL	16244	94.76
LR	899	5.24

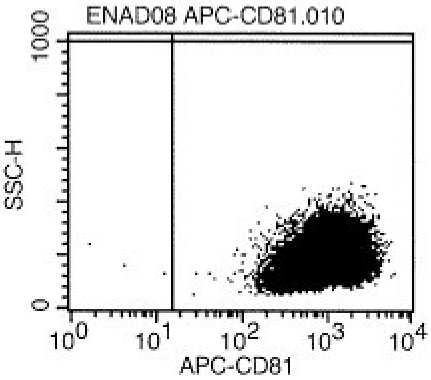

Quad	Events	% Gated
UL	0	0.00
UR	0	0.00
LL	4	0.02
LR	16343	99.98

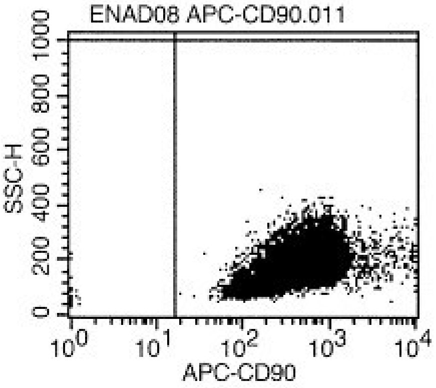

Quad	Events	% Gated
UL	0	0.00
UR	0	0.00
LL	26	0.16
LR	16355	99.84

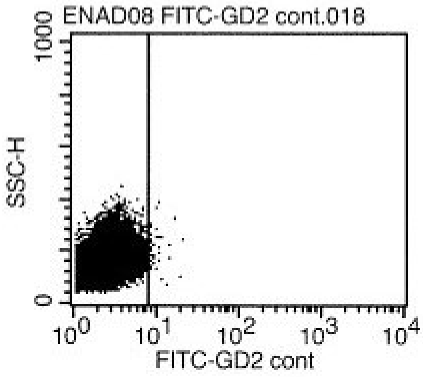

Quad	Events	% Gated
UL	0	0.00
UR	0	0.00
LL	16788	99.73
LR	46	0.27

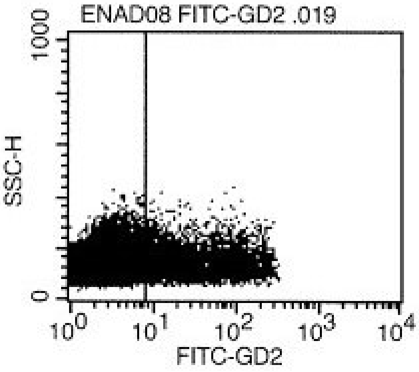

Quad	Events	% Gated
UL	0	0.00
UR	0	0.00
LL	12080	76.99
LR	3611	23.01

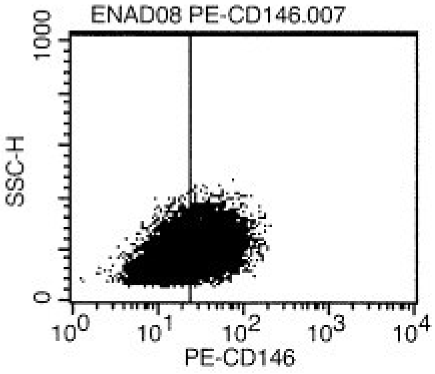

Quad	Events	% Gated
UL	0	0.00
UR	0	0.00
LL	8813	51.23
LR	8390	48.77

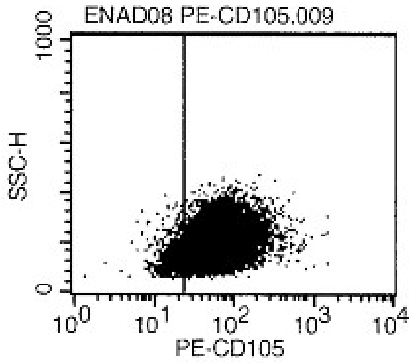

Quad	Events	% Gated
UL	0	0.00
UR	0	0.00
LL	887	5.78
LR	14450	94.22

Sheet3-2 -1

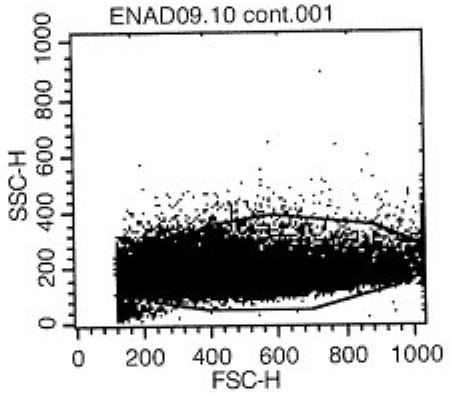

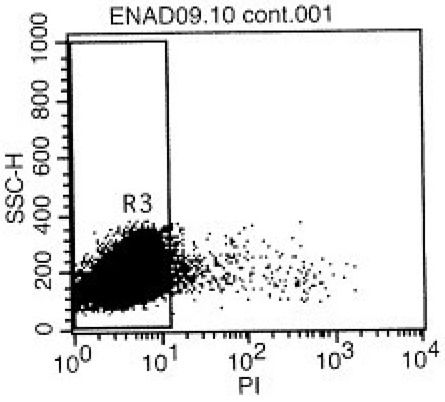

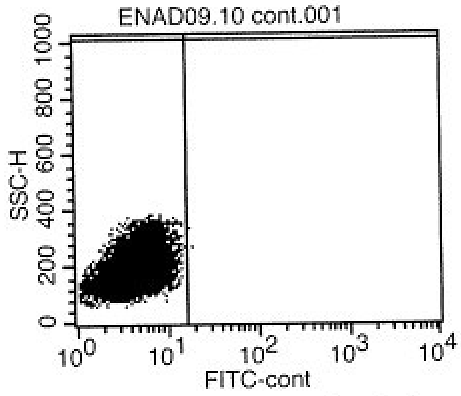

Quad	Events	% Gated
UL	0	0.00
UR	0	0.00
LL	16545	99.98
LR	3	0.02

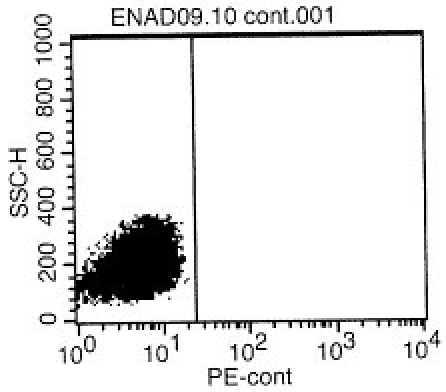

Quad	Events	% Gated
UL	0	0.00
UR	0	0.00
LL	16119	100.00
LR	0	0.00

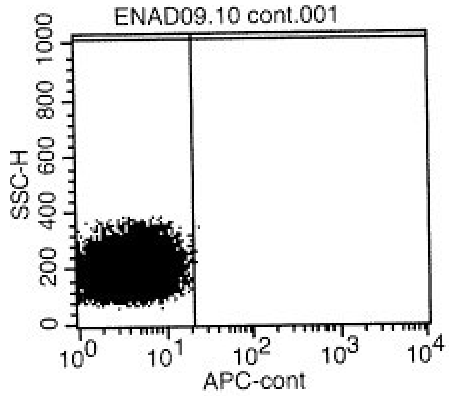

Quad	Events	% Gated
UL	0	0.00
UR	0	0.00
LL	16070	99.97
LR	5	0.03

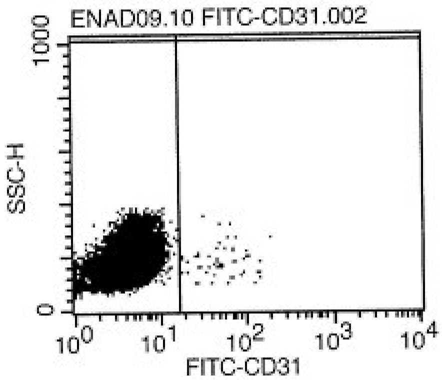

Quad	Events	% Gated
UL	0	0.00
UR	0	0.00
LL	16066	99.66
LR	55	0.34

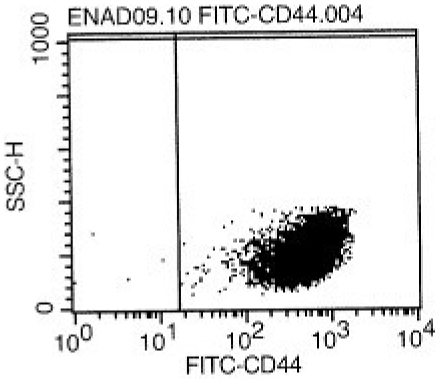

Quad	Events	% Gated
UL	0	0.00
UR	0	0.00
LL	5	0.05
LR	9215	99.95

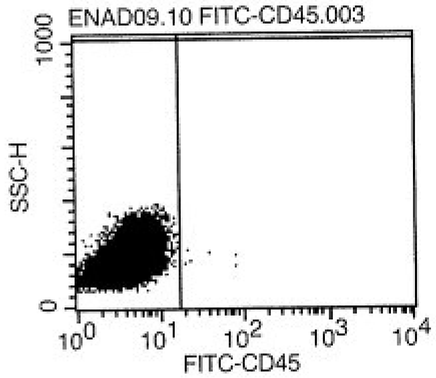

Quad	Events	% Gated
UL	0	0.00
UR	0	0.00
LL	16814	99.96
LR	6	0.04

Sheet3-2 -2

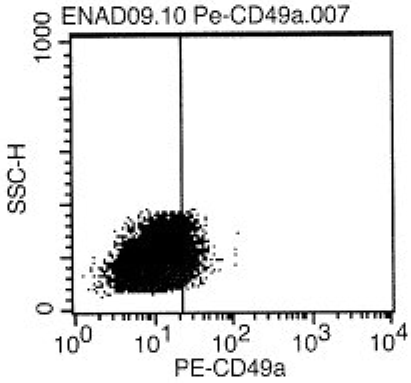

Quad	Events	% Gated
UL	0	0.00
UR	0	0.00
LL	14177	92.48
LR	1153	7.52

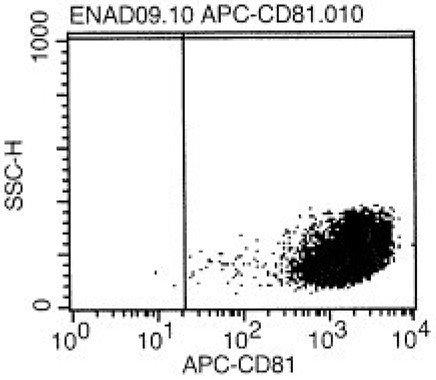

Quad	Events	% Gated
UL	0	0.00
UR	0	0.00
LL	3	0.02
LR	16461	99.98

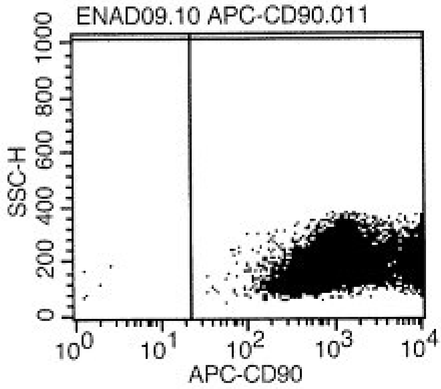

Quad	Events	% Gated
UL	0	0.00
UR	0	0.00
LL	5	0.03
LR	15819	99.97

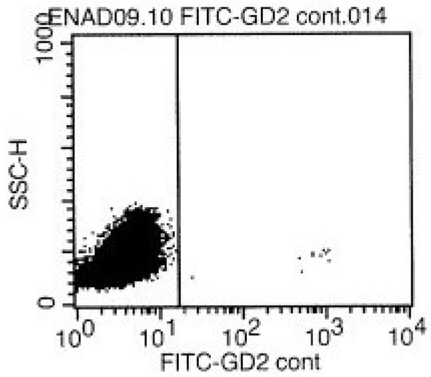

Quad	Events	% Gated
UL	0	0.00
UR	0	0.00
LL	14213	99.93
LR	10	0.07

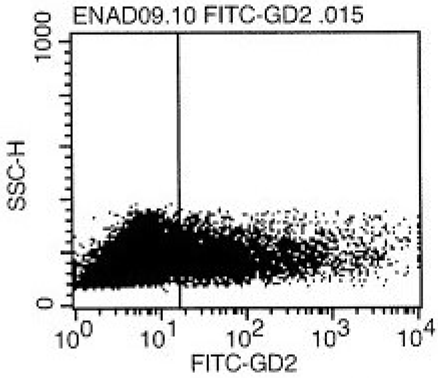

Quad	Events	% Gated
UL	0	0.00
UR	0	0.00
LL	11954	74.48
LR	4097	25.52

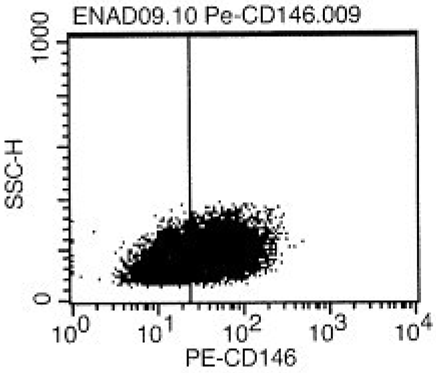

Quad	Events	% Gated
UL	0	0.00
UR	0	0.00
LL	6187	38.69
LR	9805	61.31

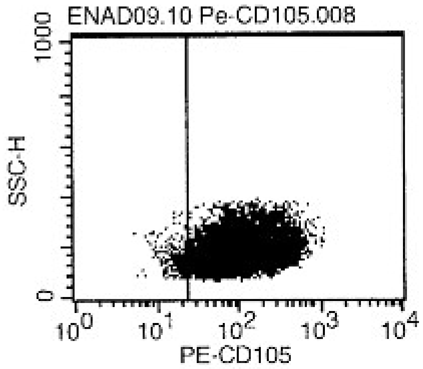

Quad	Events	% Gated
UL	0	0.00
UR	0	0.00
LL	243	1.58
LR	15175	98.42

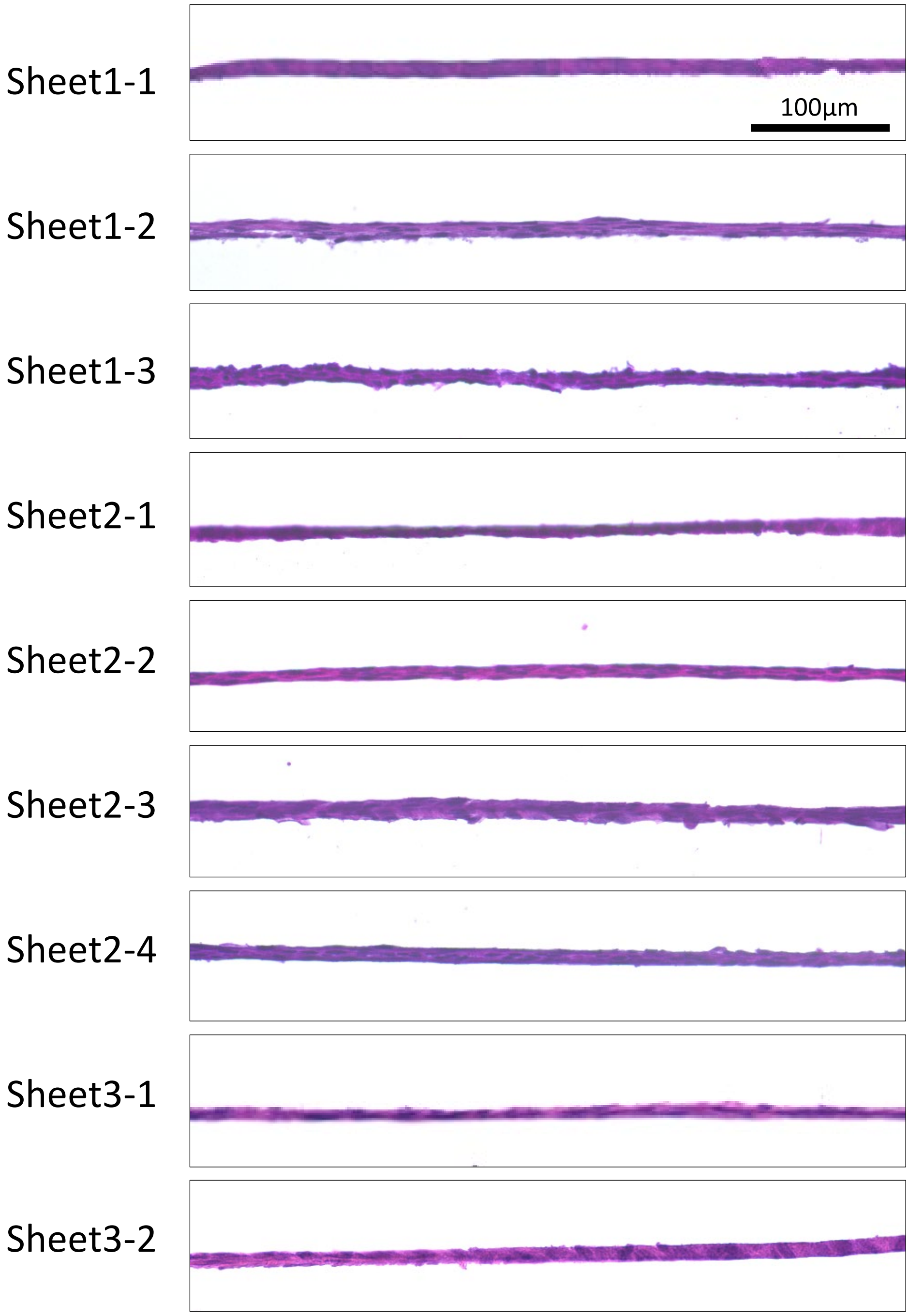

Supplementary Figure 3.
The histological appearance of all PD sheets prior to implantation.
