# Supplementary material for: Polydactyly-derived allogeneic chondrocyte cell-sheet transplantation with high tibial osteotomy as regenerative therapy for knee osteoarthritis
Source: NPJ Regen Med. 2022 Dec 16;7:71. doi: 10.1038/s41536-022-00272-1 (PMC9755241; doi:10.1038/s41536-022-00272-1)
Supplement: Supplementary file 4 — Supplemental Information Files [file 41536_2022_272_MOESM4_ESM.docx]

# SUPPLEMENTARY INFORMATION FILES

The clinical trial protocol. (PDF) Study protocol.

Supplementary Materials. (PDF) Supplementary Materials.

Supplementary Table 1 Source data for Fig. 3a.

Supplementary Table 2 Source data for Fig. 3b.

Supplementary Table 3 Probes used in qPCR.

Supplementary Table 4 Source data for Fig. 3c.

Supplementary Table 5 Source data for Fig. 3d.

Supplementary Table 6 Source data for Fig. 3e.

Supplementary Table 7 Source data for Fig. 3f.

Supplementary Figure 1 Preoperative and postoperative MR images of all cases.

Supplementary Figure 2 Raw data for Fig. 2f.

Supplementary Figure 3 The histological appearance of all PD sheets prior to implantation.

Supplementary Movie. (MP4) Raw data for Fig. 4b–g.
